# Specific *in vivo* detection of V2R-positive metastatic ccRCC using a toxin-based PET radioligand

**DOI:** 10.7150/thno.126311

**Published:** 2026-02-04

**Authors:** Goran Stanajic Petrovic, Khawla Chmeis, Alix Gonand, Ingrid Leguerney, Alexandre Ingels, Dimitri Kereselidze, Benoit Jego, Caroline Denis, Apolline Urman, Marion Chaigneau, Romain Baudat, Antoine Guyot, Pascal Kessler, Mathilde Keck, Denis Servent, Mylène Richard, Nicolas Gilles, Charles Truillet

**Affiliations:** 1 Université Paris Saclay, CEA, Département Médicaments et Technologies pour la Santé (DMTS), SIMoS, 91191 Gif-sur-Yvette, France.; 2 Université Paris-Saclay, Inserm, CNRS, CEA, Laboratoire d'Imagerie Biomédicale Multimodale (BioMaps), Service Hospitalier Frédéric Joliot, 4 Place du Général Leclerc, 91401 Orsay France.

## Abstract

**Background:**

The diagnosis of metastatic clear cell renal cell carcinoma (mccRCC) remains challenging due to the tumor's molecular heterogeneity, often resulting in low sensitivity and a high false-positive rate. In this study, we introduce and validate a new imaging modality for mccRCC based on the first radioligand targeting the type 2 vasopressin receptor (V2R) suitable for positron emission tomography (PET). V2R is ectopically expressed in mccRCC. This imaging approach utilizes [^18^F]F-MQ232, a radiolabeled peptide derived from snake venom which exhibits high *in vivo* selectivity for V2R.

**Methods:**

The V2R-selective peptide MQ232 conjugated with either a cyanine 5 (Cy5) or a fluorine 18 (^18^F) group were chemically synthetized. V2R mRNA was quantified and protein expression assessed by flow cytometry using Cy5-MQ232. The selectivity and tumor targeting ability of the modified MQ232 peptides were assessed using *in vivo* fluorescence imagery in tumor-bearing mice using CHO-V2R tumors with graded expression. Metabolic stability and PET pharmacokinetics of [^18^F]F-MQ232 were assessed in rodents. Specific tumor targeting and imaging contrast were validated *in vivo* using V2R-expressing tumors.

**Results:**

[^18^F]F-MQ232 is a highly relevant radioligand whose tumor uptake directly correlates with V2R expression levels in tissues, demonstrating its specificity to V2R-expressing tumors. Replacing the peptide moiety by an isoform unable to interact with V2R leads to a drastic decrease in the radioligand's tumor uptake, highlighting its origin in a specific, ligand/receptor type interaction between the MQ232 moiety and V2R. PET/CT imaging of Caki-1 xenografted mice demonstrated the ability of [^18^F]F-MQ232 to allow specific detection of the tumor compartment associated with high tumor-to-background contrast. RT-qPCR screening of metastatic and non-metastatic ccRCC biopsies from patients confirms V2R expression.

**Conclusions:**

This work validates the V2R-targeting strategy in mccRCC using [^18^F]F-MQ232 and demonstrates that human mccRCC tissues express V2R, confirming the suitability of this specific imaging technique for metastasis extension assessment.

## Introduction

Kidney cancers represent more than 2% of all cancer-related deaths each year with 156,000 in 2022 alone 1,2. Although the classification of their tumors is continuously evolving 3, renal cell carcinoma (RCC), transitional cell cancer (TCC) and Wilms' tumor (nephroblastoma) constitute the three main types. RCC is the predominant form accounting for approximately 80% of all cases and is mainly represented by the clear cell subtype (ccRCC). ccRCC cases are associated with a high mortality rate 4 due to frequent relapses 5,6, a high probability of metastatic dissemination 7 and an intrinsic resistance to both chemotherapy and radiotherapy 8,9. Several therapeutic strategies, particularly those leveraging immunotherapy, have emerged to tackle metastatic ccRCC (mccRCC) cases but resistance mechanisms compromise long-term disease control 10,11. Early detection, dynamic monitoring, innovative therapies and cancer cell vulnerabilities' discovery are essential strategies to overcome drug resistance in this pathological context 12. Diagnostic options for mccRCC are currently limited, yet it is crucial to obtain a clear and comprehensive assessment including the number, location, and size of the metastases.

mccRCC extension evaluation is typically performed using computed tomography (CT) and single photon emission computed tomography (SPECT)-based bone scintigraphy 13. CT presents several limitations in terms of metastasis and lymph node invasion assessments. Bone scintigraphy allows the detection of lesions developing in the specific site but provides no information on other metastatic locations. This is unfortunate, as the two predominant sites for such lesions are the lungs and lymph nodes, with some metastasis also being found in the liver 14. This limitation has led to the exploration of more sensitive, complementary molecular imaging techniques. Positron emission tomography (PET) is an imaging technique that uses a radioligand, a molecule containing a β^+^ radionuclide such as fluorine-18 ([^18^F]F, t_1/2_ = 110 min) that can be precisely tracked with high sensitivity after injection. However, PET performance critically depends on the properties of the injected radioligand. [^18^F]F-fluorodeoxyglucose (FDG) PET imaging coupled with CT (FDG-PET/CT) is by far the main approach used for cancer diagnosis 15-17. FDG, being an analogue of glucose, reveals glucose metabolism rate differences in the organism: if the targeted lesions do not offer a sufficient difference the technique is then useless. In the diagnosis and evaluation of mccRCC, more than 50% of false-negative results can occur during FDG-PET/CT examinations due to low radioligand uptake by tumor cells, attributed to their weak metabolic activity 15-17. This leads to poor visualization of the primary tumors and an even poorer detection of metastases.

To address this limitation, it is essential to identify a specific, metastases-associated molecular signature. As demonstrated in various cancer cell types 18-20, extensive evidence supports the expression of the type 2 vasopressin receptor (V2R) in both clear cell renal cell carcinoma (ccRCC) and metastatic clear cell renal cell carcinoma (mccRCC) cells 21-23. This G-protein coupled receptor (GPCR) is in a non-pathological situation predominantly expressed in the collecting ducts and distal convoluted tubules of the kidneys, where it plays a critical role in regulating water homeostasis through its endogenous ligand, vasopressin (AVP) 24-26. This organ-specific expression profile of V2R in the kidney makes it a promising biomarker for several cancer types. Its ectopic expression outside renal tissue should be easily detectable, paving the way for the development of a V2R-specific radioligand.

Previous work had already been conducted regarding vasopressin receptors' (VRs)-targeting radioligand development. Tritiated vasopressin ([^3^H]AVP) does not represent a viable option for *in vivo* imaging. A notable radioligand was developed by Gniazdowska *et al.* in 2014 through ⁹⁹ᵐTc labeling 27. This radioisotope is compatible with SPECT, another sensitive molecular imaging modality. However, to our knowledge, no further characterization of ^99m^Tc-AVP *in vivo* has been reported. A major challenge in developing a V2R-specific radiolabeled molecular probe beyond its GPCR nature lies in the existence of closely related receptors, namely V1aR, V1bR and OXTR, which are more widely distributed throughout the murine and human tissues.

In order to develop a unique V2R-specific, PET-compatible imaging tool we exploited the remarkable properties of the MQ232 peptide 28. MQ232 is a 57-residue peptide derived from the natural-occurring form called MQ1 and discovered in the venom of the Eastern green mamba, *Dendroaspis angusticeps* 29. MQ232 is the most selective V2R antagonist yet identified, exhibiting sub-nanomolar affinity for murine and human V2R 28. In healthy mice, biodistribution by PET/CT imaging of [^89^Zr]Zr-MQ232 demonstrated its strong selectivity for the kidney organ 28. Using various sensitive imaging modalities, we also further showed that [^18^F]F-MQ232 enables specific imaging of mccRCC tumor models. Moreover, arginine vasopressin receptor 2 gene (*AVPR2*) expression was clearly confirmed in ccRCC biopsies from various etiologies including metastatic cases, reinforcing the potential of this new imaging modality.

## Material and Methods

Unless otherwise mentioned, all chemicals were from Sigma-Aldrich (Merck, USA). AVP came from Bachem (Bubendorf, Switzerland) and [^3^H]AVP from PerkinElmer (Courtaboeuf, France). Fmoc-amino acids, Fmoc-pseudoprolinedipeptides, and 2-(6-chloro-1-H-benzotriazole-1-yl)-1,1,3,3,tetramethyl-aminium hexafluorophosphate (HCTU) came from Activotec (Cambridge, UK).

All cell culture reagents were from Gibco by Life Technologies (USA) and all animals from Janvier-Labs (France).

### Cell culture

CHO-304 and CHO-3013 cell lines given by Mouillac *et al.* and stably expressing hV2R were cultured in Dulbecco's Modified Eagle Medium (DMEM) supplemented with 10% fetal bovine serum (FBS), 0.1 mM non-essential amino acids (NEAA), 2 mM L-glutamine and 0.4 mg/mL geneticin. CHO-K1 (ATCC CCL-61) were cultured in DMEM supplemented with 10% FBS, 0.1 mM NEAA, 2 mM L-glutamine and 1% penicillin/streptomycin. Renca cells (murine RCC, CRL-2947, ATCC, USA) were cultured in RPMI-1640 medium supplemented with 10% (FBS, 1 mM sodium pyruvate, 0.1 mM NEAA, 2 mM L-glutamine and 1% penicillin/streptomycin. Caki-1 cells (human ccRCC, HTB-46, ATCC, USA) were cultured in McCoy's 5A supplemented with 10% FBS +1% penicillin/streptomycin. All cell lines were tested every two months for mycoplasma infection using the MycoStrip® Detection Kit (Invivogen, France).

### Biopsy preservation conditions

Kidney was removed after the patient underwent radical nephrectomy for renal cancer. Once extracted, the surgical specimen was preserved in a cold tissue preservative solution at 4 °C (Custodiol®, EUSA PHARMA SAS, Lyon, France), and then an 8 mm diameter core was extracted under sterile conditions from the tumor's area of interest. The tumor sample was chosen in agreement with the pathologist, in a non-cystic, non-necrotic area that would not interfere with the analysis of the tumor resection margins. The tumor core was then transported, isolated in the same cooled preservation liquid (Custodiol®), to the animal experimentation laboratory.

### Tumor implantation

Cells at 80% confluence were washed with 37 °C phosphate buffer saline (PBS) and treated with a 0.05% trypsin-ethylenediaminetetraacetic acid (EDTA) solution for 5 minutes at 37 °C. Dissociated cells were homogenized in their respective cooled medium, centrifugated at 4 °C, 380 g for 5 minutes with a Heraeus Megafuge 8R (Thermo Fisher Scientific, USA) and counted with FAST-READ 102 disposable counting slides (Biosigma, Italia) using a ½ dilution with a Trypan Blue 0.4% solution (MilliporeSigma, USA). The required cell quantity was then centrifugated once more and suspended in 75 µL PBS per implantation. 75 µL 4 °C liquid Matrigel® (Corning, USA) is added per implantation, the solution homogenized by flicking and kept on ice at all times. CHO-304 and CHO-3013 cells were implanted on one of the posterior flanks or at the shoulder blade of NMRI-*Foxn1^nu/nu^* mice at 5.10^6^ cells per implantation site. Caki-1 and Renca cell lines were implanted either on one of the posterior flanks of respectively NMRI-*Foxn1^nu/nu^* and BALB/cJ mice at 1.10^6^ cells per site.

For implantation, mice were anesthetized with isoflurane (Aerrane, Baxter, USA), shaved in the case of BALB/cJ and the implantation site disinfected using an alcoholic chlorhexidine solution (Cooper, USA). The 1:1 PBS:Matrigel cell suspension was collected using a refrigerated 1 mL syringe without needle (Terumo, Japan) to avoid cell lysis. A refrigerated 26 G needle (Nipro, Japan) was then added and the prepared syringe kept on ice. 150 µL of the cell suspension were subcutaneously injected, with the injection site secured using blunt-edge tweezers until the suspension solidified, and the mice were placed in heated cages until full recovery.

### Chemistry and radiochemistry

#### Solid phase synthesis of MQ232 and MQ.IMPAIRED

MQ232 is the active, engineered form of the *AVPR2*-targeting peptide as well as the molecular backbone of the radioligand characterized and evaluated here.

MQ.IMPAIRED is a highly similar peptide in which four amino acids have been modified without affecting the physicochemical properties of the initial molecule while resulting in a drastic loss of affinity for V2R with a K*_i_* for hV2R superior to 10 µM compared to the sub-nanomolar affinity of MQ232.

Both peptides were chemically produced by SPPS using a Prelude Synthesizer from Gyros Protein Technologies (USA) and then deprotected, purified and folded as described 28. MQ232 and MQ.IMPAIRED were obtained with a higher than 95% purity, controlled by reverse phase high-performance liquid chromatography (RP-HPLC) using a Waters 600 Controller coupled with a Waters 996 Photodiode Array Detector and a Vydac Protein and Peptide C18 column (Waters, USA) and mass spectrometry. To introduce an azide functionality into both peptides, 6-azidohexanoic acid was added to the resin after automated peptide synthesis and deprotection of the N-terminal amine. Coupling was performed twice for 60 min using 6-azidohexanoic acid (2 equiv.) and HCTU (1.9 equiv.) in the presence of diisopropylethylamine (2 equiv.). 6-azidohexanoic-MQs (N_3_-MQs) were then separated from the resin, purified by RP-HPLC (Waters X-bridge C18 19 × 250 mm, 10 µm column with a 15 mL/min flow rate and a gradient of 0 to 40% of acetonitrile in water over 40 min) and oxidized, giving N_3_-MQ232 and N_3_-MQ.IMPAIRED.

#### Mass spectrometry quality analysis

LC-MS analyses were performed with an Agilent 1100 Series HPLC equipped with a photodiode array detector coupled in-line with an Esquire HCT mass spectrometer (Bruker-Daltonik GmbH, Germany). The mass spectrometer is equipped with an ion trap coupled electrospray ionization unit. Reverse phase liquid chromatographic separation was performed using a C18 analytical column (Agilent Eclipse XDB 4.6 x 150 mm, 5 µm, 80 Å) and a 600 µL/min flow following a linear gradient (0% to 100% acetonitrile in 100% to 0% water containing 0.1% trifluoroacetic acid (TFA)) after a 5-minute elution in equilibrium conditions to perform a desalting of the sample.

Mass spectrometry detection was done in positive mode on a 400 to 2000 m/z range. Data analysis was performed using the DataAnalysis (Bruker-Daltonik GmbH, Germany) software allowing the deconvolution of the multi-protonated profile which enables molecular mass measurement of the primary compound.

#### Labeling of MQ232 and MQ.IMPAIRED with Cyanin 5

A solution of Cy5-dibenzocyclooctyne (Cy5-DBCO) in dimethylformamide (DMF) (8,25 mM, 2 equivalents) was added to a solution of N_3_-MQ232 or N_3_-MQ.IMPAIRED in PBS (2 mg in 300 µL) and the solution was shaken at 300 RPM, at 25 °C for 3 hours in complete darkness. The conjugated toxins were purified by reverse phase liquid chromatography on a Waters X-bridge C18 19 × 250 mm, 10 µm column with a flow rate of 15 mL/min. The gradient was 0 to 40% of acetonitrile in water over 40 min, giving respectively Cy5-MQ232 and Cy5-MQ.IMPAIRED.

#### Labeling of MQ232 and MQ.IMPAIRED with ^19^F

N_3_-MQs were generated and formulated as previously described. A solution of [^19^F]F-DBCO 30 in DMF (10 mg/mL, 5 eq.) was added to N_3_-MQ solution in PBS (300 µg in 150 µL) and the solution was shaken at 300 g, at 25 °C for 3 hours. The conjugated toxins were purified by Minitrap G-25 and analyzed by HPLC.

#### Radiolabeling of MQs

The radiofluorinations of N_3_-MQs were carried out in two steps. The prosthetic group [^18^F]F-DBCO was first synthesized on an AllInOne automate (Trasis, USA) and then conjugated to N_3_-MQs via a strain-promoted azide-alkyne cycloaddition. The radiolabeling of [^18^F]F-DBCO was performed according to a recent publication^30^, with slight modifications (**Figure S1-6**). After purification by preparative HPLC and formulation in 100% acetonitrile, [^18^F]F-DBCO was obtained with a radiochemical yield of 24.5 ± 5.4%, with a typical production of 5.6 ± 1.4 GBq after 70 min and a molar activity of 132.9 ± 50.4 GBq/µmol. A concentrated solution of [^18^F]F-DBCO in acetonitrile (10 µL) was then added to a solution of N_3_-MQ in water (0.1-0.2 mg in 200 µL) and the solution was incubated for 30 minutes at 37 °C. After size-exclusion purification (Minitrap G-25), [¹⁸F]F-MQ232 was obtained in amounts of 70 to 140 µg, with an average activity of 184.6 ± 35.9 MBq at 145 min post end of bombardment (EOB) and a decay-corrected molar activity of 30.6 ± 6.1 GBq/µmol (n = 7). [¹⁸F]F-MQ.IMPAIRED was produced in amounts of 42-78 µg, yielding an average activity of 117.2 ± 8.5 MBq at 167 min post E.O.B. and a decay-corrected molar activity of 37.6 ± 7.6 GBq/µmol (n = 3).

### *In vitro* molecular biology and pharmacology

#### RT-qPCR

Primers were either designed using NEB Tm Calculator (New England Biolaboratories, USA) and NCBI's Primer Design Tool (National Center for Biotechnologies Information, USA), for 18S ribosomal ribonucleic acid (*18SrRNA*) universal primers, *hAVPR2*, murine *AVPR2* (*mAVPR2*) and hamster *B2M* (*hamB2M*), or commercially available for human β-2 microglobulin (*hB2M*) and murine *B2M* (*mB2M*). Their efficiency was determined prior to the study using a 5-point, 5-fold serial dilution approach, and the pair was retained if the calculated efficiency was between 90 and 110%.

Different housekeeping genes (HKG)' primers were obtained and tested on different samples to assess their stability. The two most stable ones, *B2M* and *18SrRNA*, were retained and used for data normalization.

Reverse transcription reactions were either performed on 5.10^6^ cell frozen pellets in the case of *in vitro* cultured cell lines or on 50 mg frozen tissue samples for excised tissues and biopsies. Total RNA extractions were performed using a Precellys homogenizer (Bertin Health and Life Sciences, France) with a 3x30 s, 5'000 g program at 4 °C and the SSIV RT Vilo Master Mix (Thermo Fisher Scientific, USA) on whole RNA quantified in duplicate with a CLARIOstar PLUS plate reader (BMG LABTECH, Germany) equipped with the LVis plate according to the manufacturer's recommendations (20 µL, 100 ng/µL). The reaction volume was then diluted to a concentration of 10 ng/µL and the qPCR reaction carried out on Hard-Shell High Profile 96 well plates (Bio-Rad, USA) using 5 µL of the complimentary DNA (cDNA) corresponding to 50 ng total RNA, both primers at 500 nM and 2X Maxima SYBR Green/Fluorescein qPCR Master Mix (Thermo Fisher Scientific, USA) according to the manufacturer's recommendations. The plate was processed and lectured in a CFX96 real time qPCR (Bio-Rad, USA) and the results analyzed using Excel (Microsoft, USA) and GraphPad Prism V10.0 (Dotmatics, USA).

The *AVPR2*-positive reference sample chosen was total RNA extraction from a healthy kidney (Biochain, Cliniscience, France) and the results are thus shown as fold variations in expression between the analyzed sample and this control.

#### Competition binding assays

Binding experiments were performed by competition between 1 nM [^3^H]AVP and an increasing concentration of the tested ligand in a 100 μL reaction mixture containing a CHO-304 cell membrane suspension known to present hV2R 29. Data were fitted using GraphPad Prism V10.0 (Dotmatics, USA) to a one-site inhibition mass action curve. IC50 values were converted to K*_i_* with 1.1 nM as the [^3^H]AVP K*_d_*.

#### Flow cytometry

80%-confluent cells were washed with warm PBS and dissociated for 12 minutes at 37 °C using Versene. Freshly dissociated cells were homogenized in PBS at 4 °C and counted as previously described. 1 million cell aliquots were sampled, centrifugated for 5 minutes at 380 g, 4 °C, homogenized in 100 µL saturation medium (75 µM bovine serum albumin (BSA) + 2 mM EDTA in DPBS) and distributed in four 1.5 mL Eppendorf tubes per cell line. Four conditions were then set up: a tube without staining to calibrate the flow cytometer, a tube with only viability staining (SYTOX Green, Thermo Fisher Scientific, 1:1000 in saturation medium for 15 minutes in the dark), a tube with viability staining, cell washing and then a 40-minute incubation with 100 nM Cy5-MQ232 in 300 µL saturation medium, and a tube with viability staining, saturation of specific MQ binding sites using 30 µM MQ232 for 20 minutes and then an incubation with 100 nM Cy5-MQ232 for 40 minutes. All incubations were done at room temperature and in the dark. Cells were then washed three times in cold PBS, homogenized in cold PBS and processed on an Attune NxT Flow Cytometer (Thermo Fisher Scientific, USA). Data were analyzed using FlowJo (BDBiosciences, USA) (**Figure S7**).

### *In vitro* imaging

#### Confocal fluorescence microscopy

Freshly dissociated CHO-304, CHO-3013, CHO-K1, Caki-1 and Renca cell lines were seeded onto 8-well Labtek II slides (Nunc, USA) at 3.10^3^ cells per well and cultured for 48 hours at 37 °C with 5% CO_2_ in 300 µL of their respective media. The cells were then washed once with PBS, and 250 µL of fresh culture media supplemented with 0.2 mg/mL BSA and 25 mM 4-(2-hydroxyethyl)-1-piperazineethanesulfonic acid (HEPES) were added. Two staining conditions were performed: one using only Cy5-MQ232 at 100 nM and one consisting of first incubating the cells with an excess of MQ232 and then adding Cy5-MQ232. In the first condition, cells were incubated with fresh medium for 20 minutes at room temperature, Cy5-MQ232 was then added at 100 nM and cells incubated for an additional 40 minutes at room temperature. In the second condition, MQ232 was first added at 30 µM and cells incubated for 20 minutes at room temperature, and then Cy5-MQ232 was added at 100 nM and incubated for an additional 40 minutes.

Cells were then washed 3 times with cold PBS, fixed in a 4% paraformaldehyde (PFA) solution for 15 minutes, neutralized with a 50 mM solution of NH_4_Cl for 5 minutes, washed once in PBS and mounted with ProLong Antifade Diamond mounting medium (Thermo Fisher Scientific, USA). Slides were then left overnight in complete darkness to dry at room temperature and lectured with mounting oil on a Zeiss LSM 700 AxioObserver microscope equipped with a 40x EC Plan-Neofluar objective. Images were processed using ZEN 3.9 (Zeiss, Germany) and ImageJ software.

#### Epifluorescence microscopy

Organs were gathered on freshly euthanized mice, and immediately frozen using -50 °C isopentane. They were then stored at -80 °C until processing. 10 µm-thick slices were obtained with a Leica CM1860 cryostat (Leica Biosystems, Germany), deposited on SuperFrost Plus tissue mounting slides (Epredia, USA) and stored at -20 °C until processing. Slides were washed once in PBS, then incubated for one hour at room temperature with a saturation medium (5% BSA,10 mM polysorbate 80 in PBS). The saturation medium was then removed. Two conditions were then determined in the same way as for cultured cells: one with only Cy5-MQ232 and one with first an excess of MQ232. The first are incubated for 40 minutes with a 100 nM solution of Cy5-MQ232 in saturation medium. The second ones are first incubated for 20 minutes with a 30 µM solution of MQ232 in saturation medium, then removed and replaced with a solution containing 30 µM MQ232 and 100 nM Cy5-MQ232 in saturation medium. All incubations are done at room temperature in total darkness.

Slides were then washed three times in PBS, fixed in PFA and mounted as previously described. The acquisition was performed on a Zeiss AxioObserver Z1 equipped with a 20x Plan-Neofluar objective and an AxioCam MR R3 epifluorescence camera using an 800 ms excitation at 650 nm for Cyanine 5 and 8 ms at 353 nm for DAPI. Light was collected at 673 nm for Cyanine 5 and at 465 nm for 4′,6-diamidino-2-phenylindole (DAPI). Images were processed using Zeiss ZEN 3.9 and ImageJ software.

### *Ex vivo* biodistribution studies

#### Biodistribution of Cy5-MQ232 on healthy mice

20 nmoles/kg (body weight) of Cy5-MQ232 in 100 µL of 0.9% NaCl solution were intravenously (i.v.) injected into healthy NMRI-Foxn1^nu/nu^ mice (*n* = 8, 31 ± 2 g). Subjects were euthanized at t + 1 (*n* = 4) or 4 (*n* = 4) hours. Organs of interest (brain, heart, liver with intact gallbladder, intestine, stomach, left kidney, spleen and thigh muscle) were retrieved, washed with PBS and positioned on the imaging plate of a Newton 7.0 fluorescent imager (100 ms, f/16, light emitted at 640 nm and collected at 650 nm). Images were analyzed using the Kuant Plant software (Vilber Lourmat, Germany). Results obtained from non-injected mice for the dose escalation trial were used to assess the fluorescence background of each organ type. Cohorts are summarized in **Flowchart S1**.

#### Dose-escalation study with Cy5-MQ232 on CHO-304 xenografted mice

0 nmoles/kg (*n* = 3), 20 nmol/kg (*n* = 4) or 60 nmol/kg (*n* = 4) of Cy5-MQ232 in 100 µL saline were i.v. injected at t_0_ in CHO-304 xenografted NMRI-Foxn1*^nu/nu^* mice (30 ± 3 g). Subjects were then kept in the dark, imaged at t_0_+4 hours on a Newton 7.0 imager (Vilber, 100 ms, f/8) and euthanized. The brain, heart, tumor, liver with intact gallbladder, intestines, stomach, left kidney, spleen and thigh muscle were retrieved, washed with PBS and imaged using the same setup (100 ms, f/16) and then immediately frozen in isopentane at -50 °C. Organs were then stored at -80 °C and processed as described in the *Epifluorescence microscopy* section. Cohorts are summarized in **Flowchart S1**.

#### Comparison of Cy5-MQ232 and Cy5-DBCO biodistribution on CHO-304 xenografted mice

60 nmol/kg of Cy5-MQ232 (*n* = 4) or 60 nmol/kg of Cy5-DBCO (*n* = 4) in 100 µL saline were i.v. injected at t_0_ into CHO-304 xenografted NMRI-Foxn1*^nu/nu^* mice (30 ± 3 g). Subjects were then treated in the same manner as in the previous section. Cohorts are summarized in **Flowchart S1**.

### *In vivo* pharmacodynamic study

The study was performed on Sprague Dawley rats acclimated to metabolic cages for three days prior to the experiment and subcutaneously injected with 30 nmol/kg of either MQ232, MQ.IMPAIRED, Cy5-MQ232, Cy5-MQ.IMPAIRED, [^19^F]F-MQ232, [^19^F]F-MQ.IMPAIRED in NaCl 0.9% or NaCl 0.9% alone (vehicle). Rats were then left in metabolic cages for 24 hours and urine was collected and between 0 and 1h (1 h), 1 and 3 h (3 h), 3 and 5 h (5 h) and 5 and 15h (15 h).

### *In vivo* imaging

#### Fluorescence imaging

Mice implanted with CHO-304 (*n* = 6) or CHO-3013 (*n* = 5) cells and exhibiting normal tumor growth were anesthetized and i.v. injected with 20 nmol/kg of Cy5-MQ232 in 100 µL saline.

Subjects were kept in the dark and imaged using a Newton 7.0 fluorescent imager (100 ms, f/8, Vilber Lourmat, Germany) 4 h p.i. Images were analyzed using the Kuant Plant software (Vilber Lourmat, Germany). Regions of interest (ROI) were drawn around the tumors, the averaged image background signal subtracted from the average tumor signal. Basal tumor fluorescence was obtained by imaging the mice before each injection using the same setup and was also subtracted from the tumor signal. Cohorts are summarized in **Flowchart S1**.

#### Comparison of Cy5-MQ232 and Cy5-MQ.IMPAIRED

Mice implanted with CHO-304 cells (*n* = 6, 29 ± 6 g) and showing appropriate tumor growth were anesthetized and i.v. injected with 20 nmol/kg of Cy5-MQ.IMPAIRED on day 1, imaged under anesthesia 4 h p.i. according to the same parameters as described in the previous section. After 2 days of recovery, mice were checked for any residual fluorescence, injected on day 4 with 20 nmol/kg of Cy5-MQ232 and imaged again under the same conditions. A competition-based inhibition study was performed on mice which were injected in the same manner with 20 nmol/kg of Cy5-MQ232 15 minutes after a first injection of 400 nmol/kg of unlabeled MQ232 (n=6). Cohorts are summarized in **Flowchart S1**.

#### PET imaging

[^18^F]F-MQ232 or [^18^F]F-MQ.IMPAIRED in 150 µL saline was i.v.-administered via the tail vein into anesthetized mice (20 nmoles/kg, 28 ± 6 g). Animals were imaged with a Siemens Inveon (Siemens, USA) microPET/CT imager using small-animal-adapted sessions at dedicated post-injection time points. 60-minute dynamic scans were performed for each mouse immediately after injection of the radiolabeled toxin. A 20-minute static acquisition was also performed at t+4 h. All TEP acquisitions were directly followed by a 10-minute CT one to perform attenuation correction. CT images were also used to help draw volumes of interest (VOI).

The Inveon microPET scanner has a spatial resolution of 1.5 mm. All the images were reconstructed using a three-dimensional ordinary Poisson algorithm with ordered subset expectation maximization, followed by an ordinary Poisson ordered subset expectation maximization maximum *a posteriori* algorithm (OP-OSEM3D-MAP) (2 OSEM3D iterations, 18 MAP iterations with 16 MAP subsets). The size of the image matrix was 256 x 256 pixels with 159 slides, resulting in a voxel size of 0.38 x 0.38 x 0.80 mm. VOIs corresponding to regions of significant tracer uptake in each organ of interest (tumors, kidneys, liver, gallbladder, left ventricle, muscle, bone junctions, and brain) were delineated on the images using PMOD software. VOIs for the liver and kidneys were defined using uptake thresholds guided by the corresponding CT images. Image-derived input functions (blood kinetics) of the left ventricle were measured from computed tomography (CT) based attenuation corrected PET images for each mouse. The distribution kinetics of the radiolabeled compound were determined by generating time-activity curves (TACS) from each VOI. The TACs are expressed as a percentage of the injected dose per volume (% ID.cm^-3^). From the TACs, areas under the curves (AUC) between time 0 and the last scan were calculated to define MQ232 uptake using GraphPad Prism V10.0.

Images are shown as maximum intensity projections (MIP), allowing three-dimensional data visualization of merged PET/CT scans by using voxels (volumetric pixels) with the highest intensity. Cohorts are summarized in **Flowchart S1**.

## Results

### New MQ232-based molecular tools for *in vitro* and *in vivo* V2R investigation

**Figure 1:**
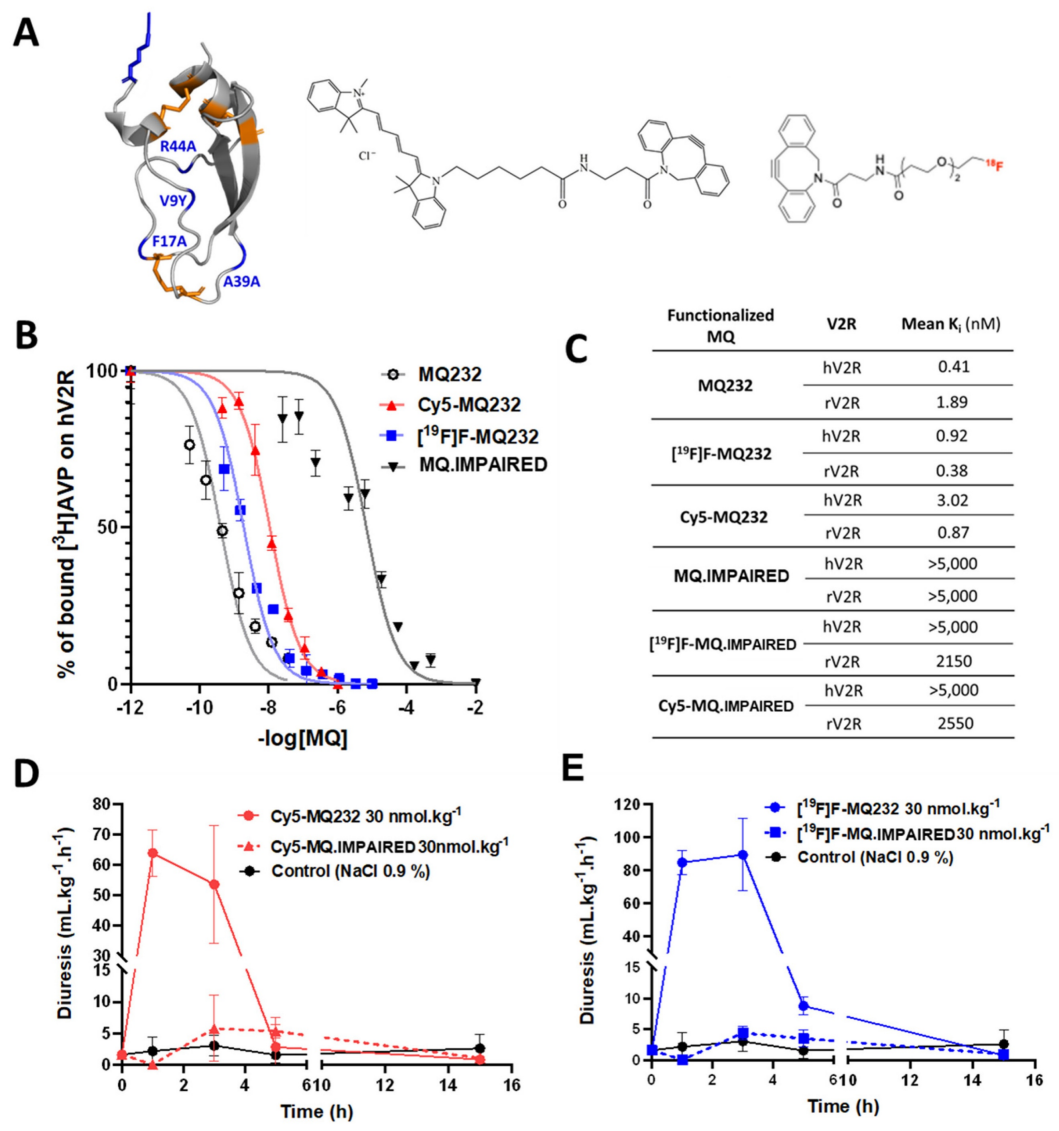
***In vitro* and *in vivo* pharmacology of MQ232 and derivatives.** A. Three-dimensional representation of MQ232 (left, the residues in blue are the ones that were modified to generate the MQ-IMPAIRED). Cy5-dibenzocyclooctyne molecule (middle). [^18^F]F-dibenzocyclooctyne molecule (right). Molecular scale is not respected. B. Competitive binding inhibition of [^3^H]AVP (1 nM) on human V2R by MQ232 (black), [^18^F]F-MQ232 (blue), or Cy5-MQ232 (red) (*n* = 4). C. Summary table of the affinity of the MQ232-derived molecules for human V2R (hV2R) and rat V2R (rV2R). D. Sprague Dawley rat diuresis after s.c. injection of Cy5-MQ232 (30 nmol/kg, solid red curve), Cy5-MQ.IMPAIRED (30 nmol/kg, dashed red curve) or vehicle (NaCl 0.9%, solid black curve). *n* = 3 rats per molecule. Aqueous diuresis was measured before the injection and at t+1 h, t+3 h, t+5 h and t+15 h. E. Sprague Dawley rat aqueous diuresis after s.c. injection of [^19^F]F-MQ232 (30 nmol/kg, solid blue curve), [^19^F]F-MQ.IMPAIRED (30 nmol/kg, dashed blue curve) or vehicle (NaCl 0.9%, solid black curve). *n* = 3 rats per molecule. Aqueous diuresis was measured before the injection and at t+1 h, t+3 h, t+5 h and t+15 h.

To selectively detect and quantify the V2R protein at the cells' surface, four MQ232-derived tools were developed using click chemistry on the N_3_-MQ232 peptide backbone (**Figure 1A, Flowchart S2, Figure S8A-H**). Cy5-MQ232 is an infrared fluorescent molecular probe adapted to *in vitro* and *in vivo* studies demonstrating low nanomolar affinities for V2R (K*_i, hV2R_* = 3 nM). The stable [^19^F]F-MQ232 counterpart of the radioligand [^18^F]F-MQ232 also demonstrates high affinities for V2R (K*_i, hV2R_* = 1 nM, **Figure 1B-C**). *In vivo* V2R blocking using MQ232 in rats increases diuresis, which can in turn be measured to assess V2R activity, our MQ232-based molecules were pharmacodynamically validated using this strategy^28^. Both Cy5-MQ232 (**Figure 1D**) and [^19^F]F-MQ232 (**Figure 1E**) retained their V2R antagonistic abilities *in vivo,* demonstrated by a time-dependent increase in diuresis after subcutaneous (s.c.) injection in healthy rats (**Table S1**). The demonstration of an *in vivo* selectivity between a ligand and its target is very challenging and is usually achieved by inhibition-based competition studies using an excess of unlabeled ligand with a potential toxicity risk at the required doses. To circumvent this drawback we took advantage of our knowledge on MQ232 and its mode of action 31 to generate a deactivated MQ232, termed MQ.IMPAIRED (**Figure S8E-H**). To preserve the peptide's physicochemical characteristics (hydrophobicity and charges), we introduced these four sequence modifications: F17A, V9Y, A39K and R44A. The resulting [^19^F]F-MQ.IMPAIRED and Cy5-MQ.IMPAIRED exhibited major diminution in their affinities for human and rat V2R with K*_i, hV2R_* dwindling from respectively 1 and 3 nM to over 5,000 nM (**Figure 1B-C, Flowchart S2**). Both MQ-IMPAIRED-based molecules failed to induce increased diuresis in rats after s.c. injection (**Figure 1D-E, Table S1**) supporting the *in vivo* inactivity of MQ-IMPAIRED based ligands. MQ.IMPAIRED thus offers a reliable and novel approach for investigating the *in vivo* selectivity of MQ232-based probes.

### V2R is expressed at two different levels in CHO cells used to challenge the MQ232-based probes

**Figure 2:**
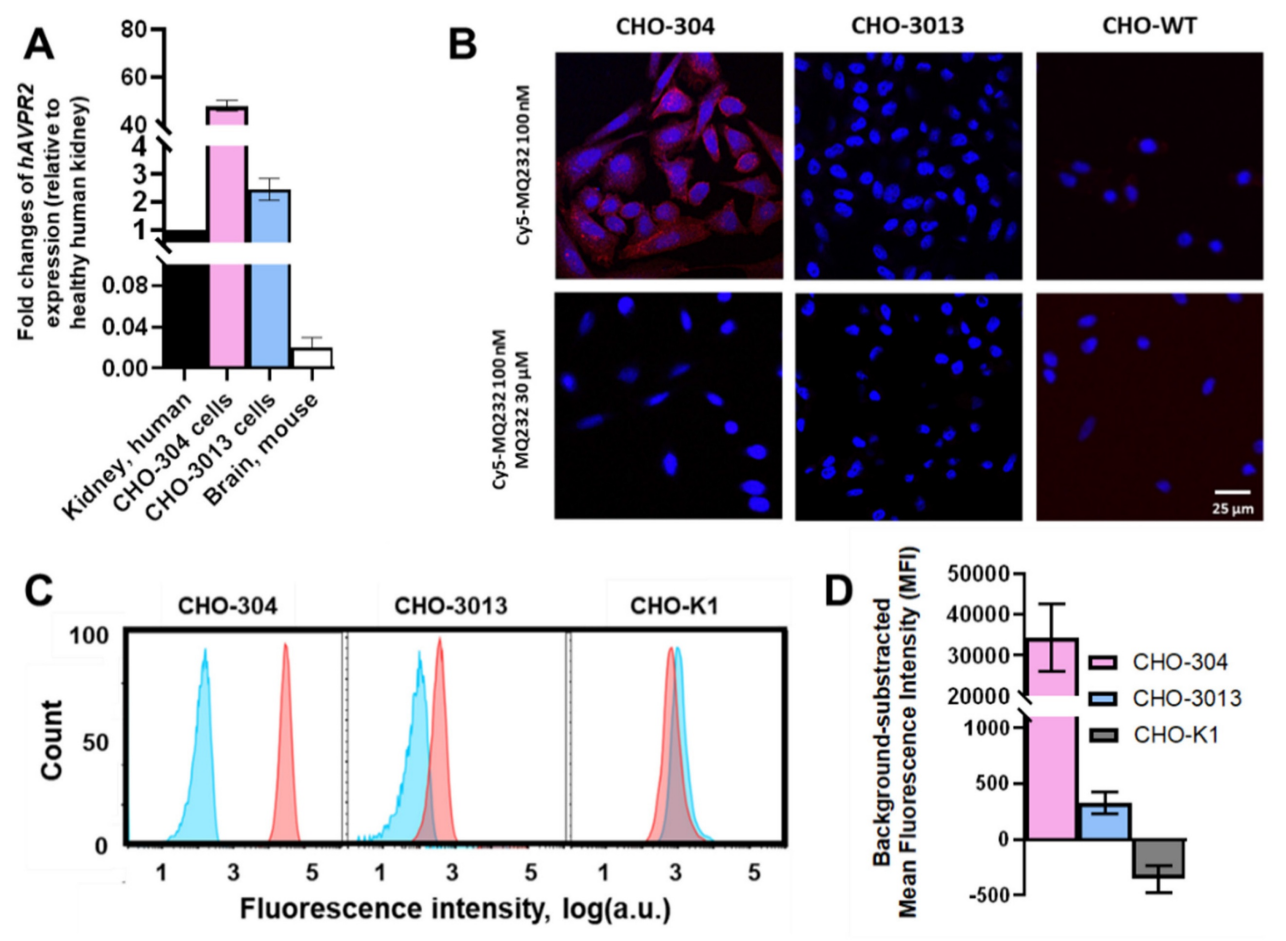
** hV2R genetic and protein expression assessment in engineered CHO cell lines.** A: Quantification results of *AVPR2*-directed RT-qPCR performed on the two *AVPR2*-expressing CHO cell lines compared to reference tissue samples. Results are shown as fold changes between the expression in the investigated samples and the expression in the positive reference, healthy human total kidney. B: Scanning confocal microscopy observation of CHO-304, CHO-3013 and the V2R-negative control CHO-K1 cell lines after Cy5-MQ232 staining. Up: Total staining using 100 nM Cy5-MQ232. Down: Non-specific staining using 100 nM Cy5-MQ232 in presence of 30 µM MQ232. Blue: DAPI (nuclear staining, 10 ms exposure at 352 nm, light gathered at 465 nm). Red: Cy5 (800 ms exposure at 650 nm, light gathered at 673 nm). C: Flow cytometry results obtained on freshly dissociated CHO-304, CHO-3013 and CHO-K1 cells stained using 100 nM Cy5-MQ232 (total signal, red curve) or 100 nM Cy5-MQ232 in presence of 30 µM of MQ232 (non-specific signal, blue curve). Light gathered at 680 nm, 30,000 events per acquisition, two acquisitions per independent experiment, *n* = 3). D: Specific V2R-linked fluorescence signal obtained through the subtraction of the mean fluorescence intensity after non-specific labeling from the mean fluorescence intensity after total labeling.

CHO-304 and CHO-3013, two cell lines stably expressing *hAVPR2* at different expression levels and harboring different levels of V2R protein at their membrane 27,28 were used as V2R expression references for *in vitro* and *in vivo* experiments. Gene expression quantification was performed on the two cell lines using RT-qPCR on total RNA, with results presented as fold changes relative to *hAVPR2* expression in healthy human kidneys. The CHO-304 cell line showed a 60 ± 19-fold higher *hAVPR2* expression than human kidney, while the CHO-3013 cell line exhibited a 3.1 ± 1.2-fold increase, giving a high and a low expression reference (**Figure 2A**, **Table S2-3**).

Cy5-MQ232 was first used for fixed cell staining of the two V2R-positive CHO cell lines (**Figure 2B**). We were able to visualize a specific staining in the case of CHO-304 cells consistent with the *hAVPR2* gene expression but no specific staining in the case of CHO-3013. Since the expression level in CHO-3013 cells was 20 times lower than in CHO-304 cells, we assumed that this technique's sensitivity limit had been reached. We thus switched to flow cytometry using Cy5-MQ232 on dissociated live cells (**Figure 2C**). This strategy allows both a better detection sensitivity and a quantified assessment of the toxin-targetable pool of V2R. A significative signal linked to specific binding of the fluorescent probe to CHO-3013 and CHO-304 cells was detected with a respective mean fluorescent intensity of 330 ± 100 a.u. and 34,000 ± 8,000 a.u. (**Figure 2D**). These results confirm that both cell lines harbor a targetable pool of V2R protein at their surface in an amount linked to the *hAVPR2* expression level previously assessed (**Figure 2A**).

Using these two CHO-based models, the *in vivo* potential of MQ232-based probes was first investigated with Cy5-MQ232. This included proving the molecule's ability to label V2R-positive tumors *in vivo* and then that the observed signal was the sole consequence of a selective interaction between the MQ232 part of the imaging molecule and V2R.

### Tumor uptake of MQ232-based molecules is mediated through *in vivo* selective binding of MQ232 to V2R

**Figure 3:**
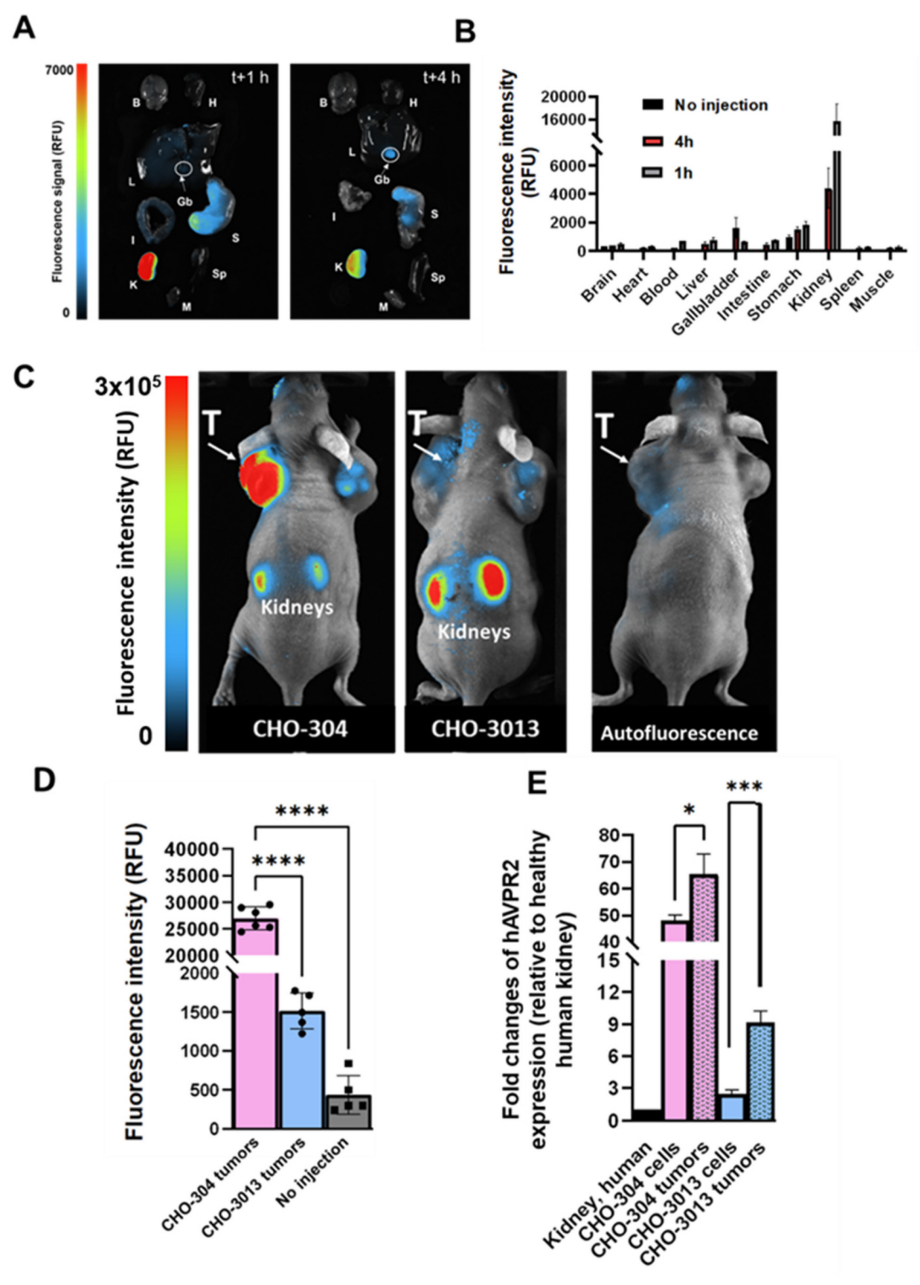
***In vivo* fluorescence imaging in healthy and V2R+ tumor bearing mice.** A: Representative composite images of organs obtained from healthy NMRI-Foxn1^nu/nu^ mice euthanized at t+1 h (*n* = 4, left) or t+4 h (*n* = 4, right) after i.v. injection of 20 nmol/kg Cy5-MQ232. Organs were washed with PBS before the acquisition. (B)Brain (H)Heart (L)Liver (Gb)Gallbladder (I)Intestine (S)Stomach (K)Kidney (Sp)Spleen (M)Muscle. B: Quantification of the fluorescence in the organs of interest. C: Composite images of representative NMRI-Foxn1*^nu/nu^* mice xenografted with either the CHO-304 (middle) or the CHO-3013 cell line (right) 4 hours after the i.v. injection of 20 nmol/kg Cy5-MQ232. A representative image of a non-injected mouse is also displayed (left). The signal observed in the tumor compartment of the representative non-injected mouse is due to the natural fluorescence of hemoglobin at the wavelengths used and is independent of the implanted cell line. (T)Tumor. D: Quantification of the average tumor fluorescence signal 4 hours after the i.v. injection of 20 nmol/kg Cy5-MQ232 in NMRI-Foxn1^nu/nu^ mice xenografted with either the CHO-304 or the CHO-3013 cell line. The control group is composed of the same mice evaluated before injection of the fluorescent compound. No injection controls performed on CHO-304 and CHO-3013 tumors were pooled being independent of the implanted cell line. Statistic test: T test. E: Comparison of *hAVPR2* expression (expressed in fold change compared to healthy human kidney) between CHO-304 and CHO-3013 cells and the corresponding tumors. \

We first assessed the fluorescent probe's behavior as well as its biodistribution in healthy mice by injecting 20 nmol/kg body weight (BW) of Cy5-MQ232 (*n* = 8) in comparison with animals injected with the same volume of NaCl 0.9% (*n* = 3) (**Figure 3A-B**). The organs of interest were retrieved and imaged 1 hour (*n* = 4) or 4 hours (*n* = 4) after injection (**Figure 3A**) and fluorescent probe's uptake was assessed through mean fluorescence intensity quantification (**Figure 3B, Figure S9, Table S4**). The highest fluorescence accumulation is found in the kidney one hour after the injection at 15,600 ± 3,000 RFU, the signal persisting 4 hours post injection (p.i.) with 4,400 ± 1,400 RFU, which is expected as the renal compartment represents both the main excretion site and the major canonic pool of V2R protein. The gallbladder showed Cy5-MQ232 uptake only at t+4 hours with 1,620 ± 720 RFU. This signal is almost four times higher than that of the liver, which was 470 ± 140 RFU at the same time point, suggesting minor hepatic metabolization. Minimal signals are observed in all other investigated organs with no uptake in brain, heart tissue, intestines, muscle tissue and spleen. Cy5-MQ232 displays a strong selectivity for the kidney compartment with non-specific signal contained in other tissues. The most favorable timepoint for specific signal evaluation is at t+4 hours.

Consequently, we tested the ability of the fluorescent probe to label *in vivo* V2R-expressing tumors using CHO-304 (*n* = 6) and CHO-3013 (*n* = 5) cells xenografting in NMRI Foxn1*^nu/nu^* mice. Tumor bearing animals were injected with 20 nmol/kg Cy5-MQ232 and imaged at 4 h p.i. (**Figure 3C-D, Table S5**). Mean fluorescent intensities of 28,000 ± 3,000 RFU and 1520 ± 230 RFU were obtained in CHO-304 and CHO-3013 tumors, respectively. Both labeling are significantly higher than the background signal obtained before injection and quantified at 440 ± 250 RFU for both models (**Figure 3D**). The 18-time fold change between the two models' fluorescent signal intensity correlates well with the 20-fold change in *hAVPR2* expression measured in the two cell lines by RT-qPCR. In addition, *AVPR2* expression in excised tumors obtained after CHO-304 and CHO-3013 cell implantation was assessed, being 65 ± 7 and 9 ± 1 times, respectively, compared to the human kidney, which is slightly higher than for the *in vitro* cultured corresponding cells (**Figure 3E, Table S3,6**).

**Figure 4:**
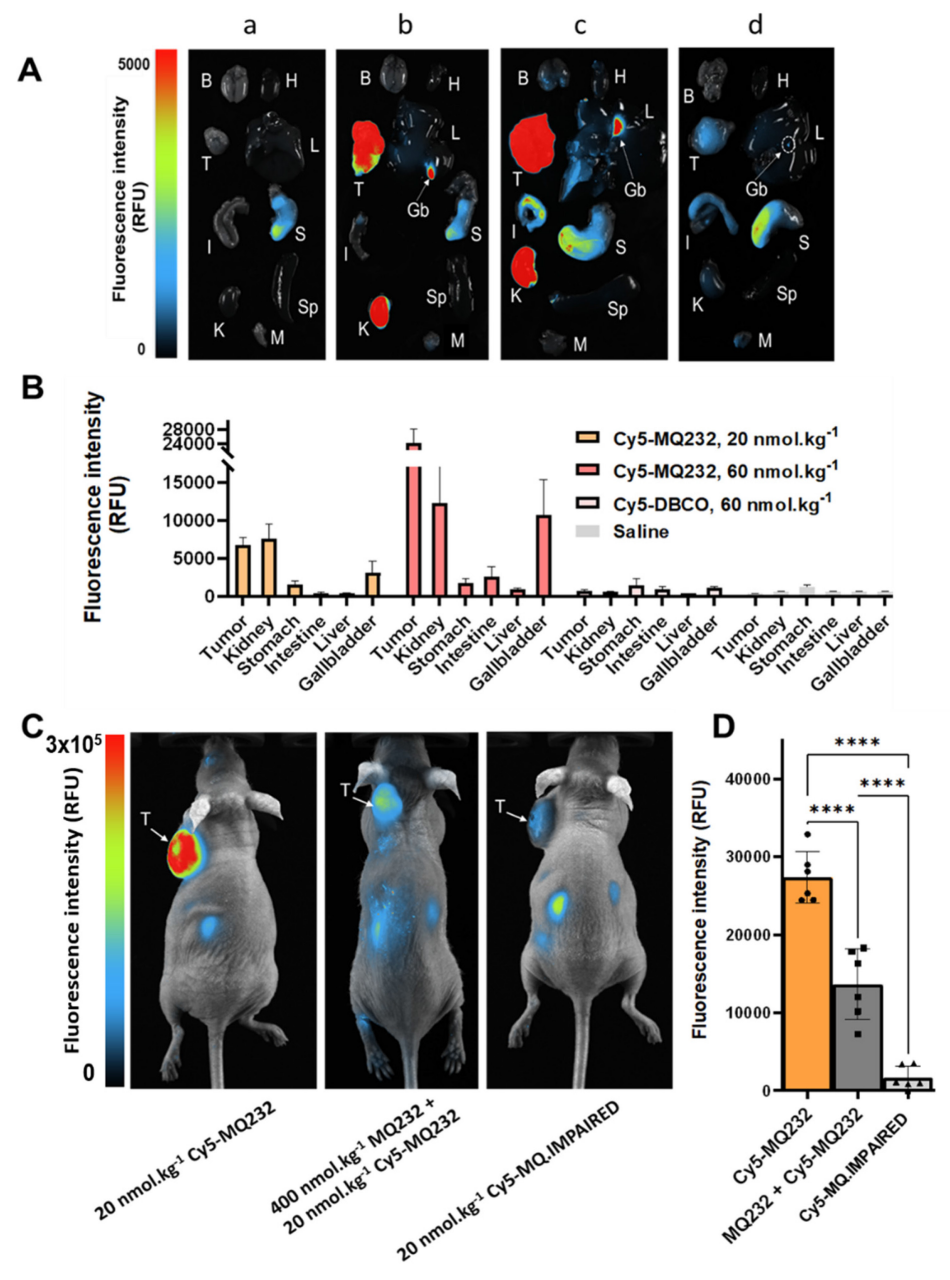
***In vivo* selectivity of MQ232-based probes.** A: Representative composite images of organs obtained on CHO-304 xenografted NMRI-Foxn1^nu/nu^ mice euthanized at t+4 h after i.v. injection of 150 µL NaCl 0.9% (a, *n* = 3), 20 nmol/kg (b, *n* = 4) or 60 nmol/kg (c, *n* = 4) of Cy5-MQ232, or 60 nmol/kg (d, *n* = 3) of Cy5-DBCO. Organs were washed with PBS before the acquisition. (B)Brain (H)Heart (T)Tumor (L)Liver (Gb)Gallbladder (I)Intestine (S)Stomach (K)Kidney (Sp)Spleen (M)Muscle. B: Quantification of the fluorescence in the organs of interest obtained from CHO-304 xenografted NMRI-Foxn1*^nu/nu^* mice euthanized at t+4 h after i.v. injection as described in A. C: Composite images of representative NMRI-Foxn1*^nu/nu^* mice xenografted with the CHO-304 cell line 4 hours after the i.v. injection of 20 nmol/kg Cy5-MQ232 (left), 400 nmol/kg MQ232 then 20 nmol/kg Cy5-MQ232 (middle) or 20 nmol/kg Cy5-MQ.IMPAIRED (right). (T)Tumor. D: Average fluorescence intensity quantification obtained from the tumor compartment of CHO-304 xenografted NMRI-Foxn1^nu/nu^ mice after injection of 20 nmol/kg of Cy5-MQ232, 400 nmol/kg of MQ232 followed by 20 nmol/kg of Cy5-MQ232 or Cy5-MQ.IMPAIRED, via i.v. injection.

A dose escalation assay was then set up to investigate off-target unspecific labeling and the correlation between Cy5-MQ232 injected dose and fluorescence intensity observed in the tumor compartment. CHO-304 tumor bearing mice were injected with either saline (*n* = 3), 20 nmol/kg Cy5-MQ232 (*n* = 4), 60 nmol/kg Cy5-MQ232 (*n* = 4) or 60 nmol/kg of unconjugated Cy5-DBCO (*n* = 3) to examine the contribution of this hydrophobic entity (**Figure 4A, Figure S10, Table S5**). Here again, tumor average fluorescent intensity correlated well with the Cy5-MQ232 dose ranging from 6400 ± 1000 RFU to 23800 ± 3600 RFU while escalating from 20 to 60 nmol/kg (**Figure 4B**). No significant tumor labeling was observed in the Cy5-DBCO injected group. A limited liver uptake was detectable in the mice injected with the highest dose of Cy5-MQ232 with a signal of 500 ± 250 RFU. A dose-dependent labeling of the gallbladder was also observed, quadrupling between the two Cy5-MQ232 doses.

To assess the *in vivo* selectivity of our probe for V2R, a high dose of MQ232 (400 nmol/kg) was injected 15 minutes before the 20 nmol/kg Cy5-MQ232 injection in CHO-304 tumor bearing mice (*n* = 6) (**Figure 4C-D**). This MQ232 quantity was chosen considering the no observed adverse effect level of this peptide 28. A significant 50% loss of tumor fluorescence, quantified at 13,600 ± 4,500 RFU is observed for this group compared to the 27,000 ± 2,100 RFU with Cy5-MQ232 alone (*n* = 6). Nevertheless, tumor fluorescence was not fully inhibited due to the inherent technique limitation or a possible Enhanced Permeability and Retention (EPR) effect. We thus performed the same experiment with 20 nmol/kg of the counterpart Cy5-MQ.IMPAIRED (*n* = 6). Here, an impressive average loss of 97 ± 5% of the tumor signal is observed (**Figure 4C-D, Figure S11**). These results directly demonstrate that the fluorescence signal obtained after Cy5-MQ232 injection arises entirely from the specific interaction between the toxin moiety of the probe and the V2R receptor, with minimal contribution from EPR effects or non-specific interactions involving the tumor microenvironment or the Cy5-DBCO prosthetic group. Taken together, these data robustly demonstrate that Cy5-MQ232 exhibits absolute selectivity for V2R both *in vitro* and *in vivo*.

**Figure 5:**
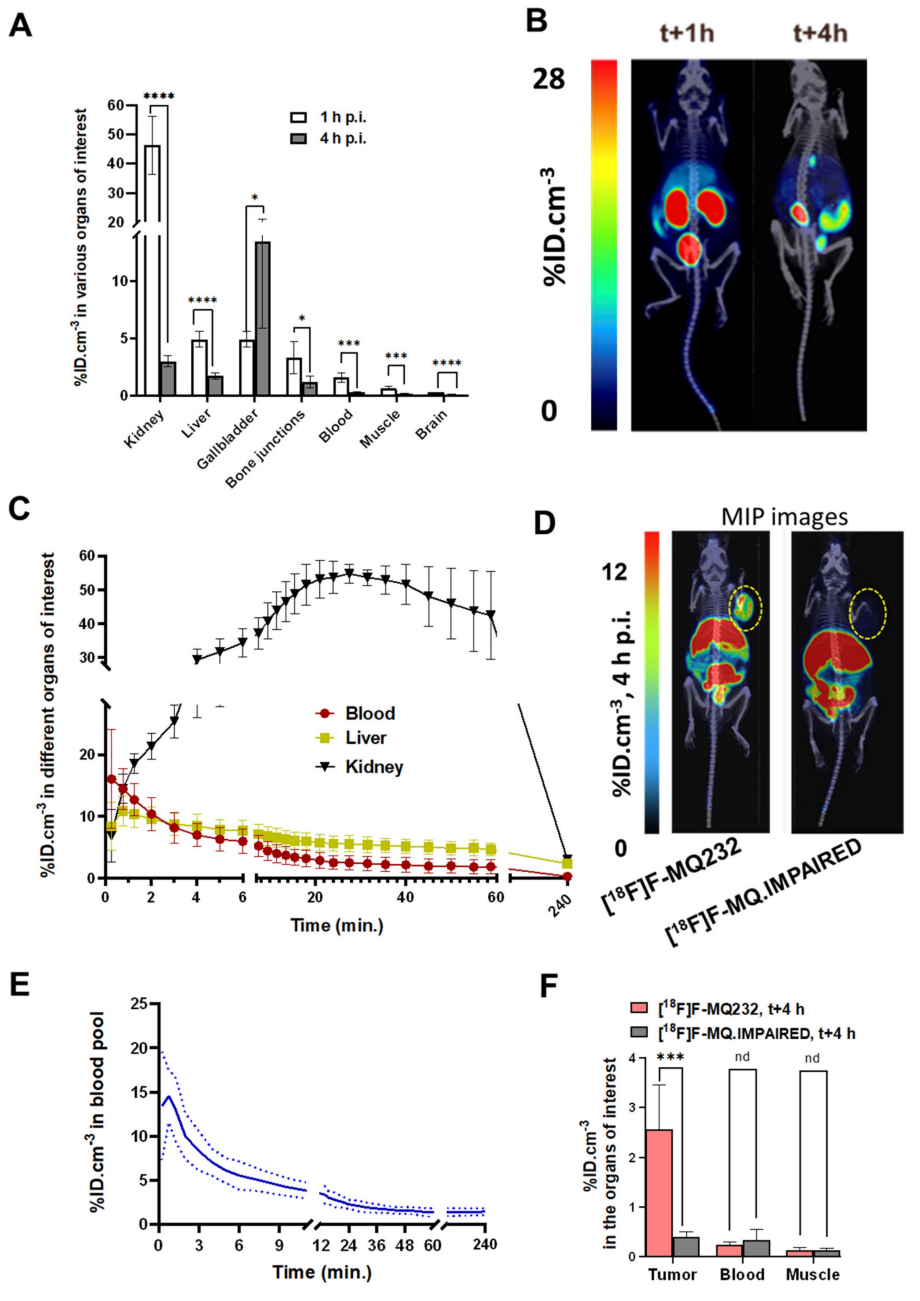
***In vivo* MQ232 pharmacokinetics in healthy and V2R+ tumor bearing mice.** A. PET-acquired biodistribution at t+1 h and t+4 h after the i.v. injection of 20 nmol/kg of [^18^F]F-MQ232 in healthy NMRI-Foxn1*^nu/nu^* mice (3.0 ± 0.7 MBq, 120 ± 28 MBq/kg, *n* = 6). B. Composite images representation of healthy NMRI-Foxn1^nu/nu^ mice after 1 hour and 4 four hours post-intraveneous injection of 20 nmol/kg [^18^F]F-MQ232 (3.0 ± 0.7 MBq, 120 ± 28 MBq/kg, *n* = 6). Images shown are the result of a fusion between the Computed Tomography (CT) image (grey scale) and the PET image shown as Maximum Intensity Projection (MIP, false colors). C. Radiotracer concentration-time profiles in the blood, liver, and kidney compartments of healthy NMRI-Foxn1^nu/nu^ mice following intravenous injection of [¹⁸F]F-MQ232 (20 nmol/kg; 3.0 ± 0.7 MBq, 120 ± 28 MBq/kg; n = 6). A 60-min dynamic acquisition was initiated at the time of injection and followed by a 20-min static acquisition at 4 h post-injection. D. Representative composite images of CHO-304 xenografted NMRI-Foxn1^nu/nu^ mice acquired at 4 h post-injection of either [¹⁸F]F-MQ232 (20 nmol/kg; 6.8 ± 1.1 MBq, 274 ± 43 MBq/kg; n = 6; left, for comparison) or [¹⁸F]F-MQ.IMPAIRED (20 nmol/kg; 5.8 ± 1.8 MBq, 234 ± 71 MBq/kg; n = 6, right). Images shown are the result of a fusion between the Computed Tomography image (grey scale) and the PET image shown as Maximum Intensity Projection (MIP, false colors). E. Blood TACs derived from PET imaging to determine the pharmacokinetic profile of [^18^F]F-MQ232 after i.v. injection in healthy NMRI-Foxn1*^nu/nu^* mice (3.0 ± 0.7 MBq, i.e. 120 ± 28 MBq/kg, *n* = 6). F. Average concentration of the radioligands in tumor, blood and muscle compartments after 4 hours post i.v. injection of 20 nmol/kg for the [^18^F]F-MQ232 (6.8 ± 1.1 MBq, i.e. 274 ± 43 MBq/kg, *n* = 6) or of 20 nmol/kg for the [^18^F]F-MQ.IMPAIRED (5.8 ± 1.8 MBq i.e. 234 ± 71 MBq/kg, *n* = 6). Statistic test: T test. * p-value < 0,05, ** p-value < 0,01, *** p-value < 0,001, **** p-value < 0,0001.

The PET radioligand [^18^F]F-MQ232 was then evaluated to see if it possessed the already predicted properties and to assess its tumor visualization capacities. [^18^F]F-MQ232's biodistribution and pharmacokinetic profiles were evaluated in healthy NMRI-*Foxn1^nu/nu^* mice (*n* = 6) (**Figure 5A-B, Figure S12,13, Table S7**). A lower but significative liver uptake can also be witnessed at t+1 h with an uptake of 4.9 ± 0.7 %ID.cm^-3^ which is halved three hours later. No difference between the liver and gallbladder uptake is observable at t+1 h. However, a strong, heterogenous gallbladder uptake is observed at t+4 h with 13.5 ± 7.6 %ID.cm^-3^. In the liver compartment, [^18^F]F-MQ232 concentration reaches a maximum of 11 ± 2.5 %ID.cm^-3^ within one minute and gradually decreases from that time point to t+4 hours. A rapid mono-exponential clearance from the blood compartment is observed with t_1/2, blood_ = 5.5 ± 1.4 min (**Figure 5C-E**).

CHO-304 tumor imaging was tested by both [^18^F]F-MQ232 (273 ± 43 MBq/kg) and [^18^F]F-MQ.IMPAIRED (234 ± 71 MBq/kg) (**Figure 5D, Figure S12-15, Table S8-9**). [^18^F]F-MQ232 tumor uptake was determined to be 2.6 ± 0.9%ID.cm^-3^ with a tumor-to-muscle ratio (TMR) of 21 ± 8.6 and tumor-to-blood ratio (TBR) of 8.4 ± 2.7 (**Figure 5F, Figure S16**). Injection of [^18^F]F-MQ.IMPAIRED resulted in a tumor uptake and the two calculated ratios at respectively 0.4 ± 0.1%ID.cm^-3^, 1.2 ± 0.3 and 3.0 ± 1.3. All the other uptakes were comparable between [^18^F]F-MQ232 and [^18^F]F-MQ.IMPAIRED and both demonstrate statistically identical blood half-lives (**Table S10-11, Figure S17**).

### [^18^F]F-MQ232 allows the *in vivo* PET detection of clinic-relevant mccRCC xenografted models

**Figure 6:**
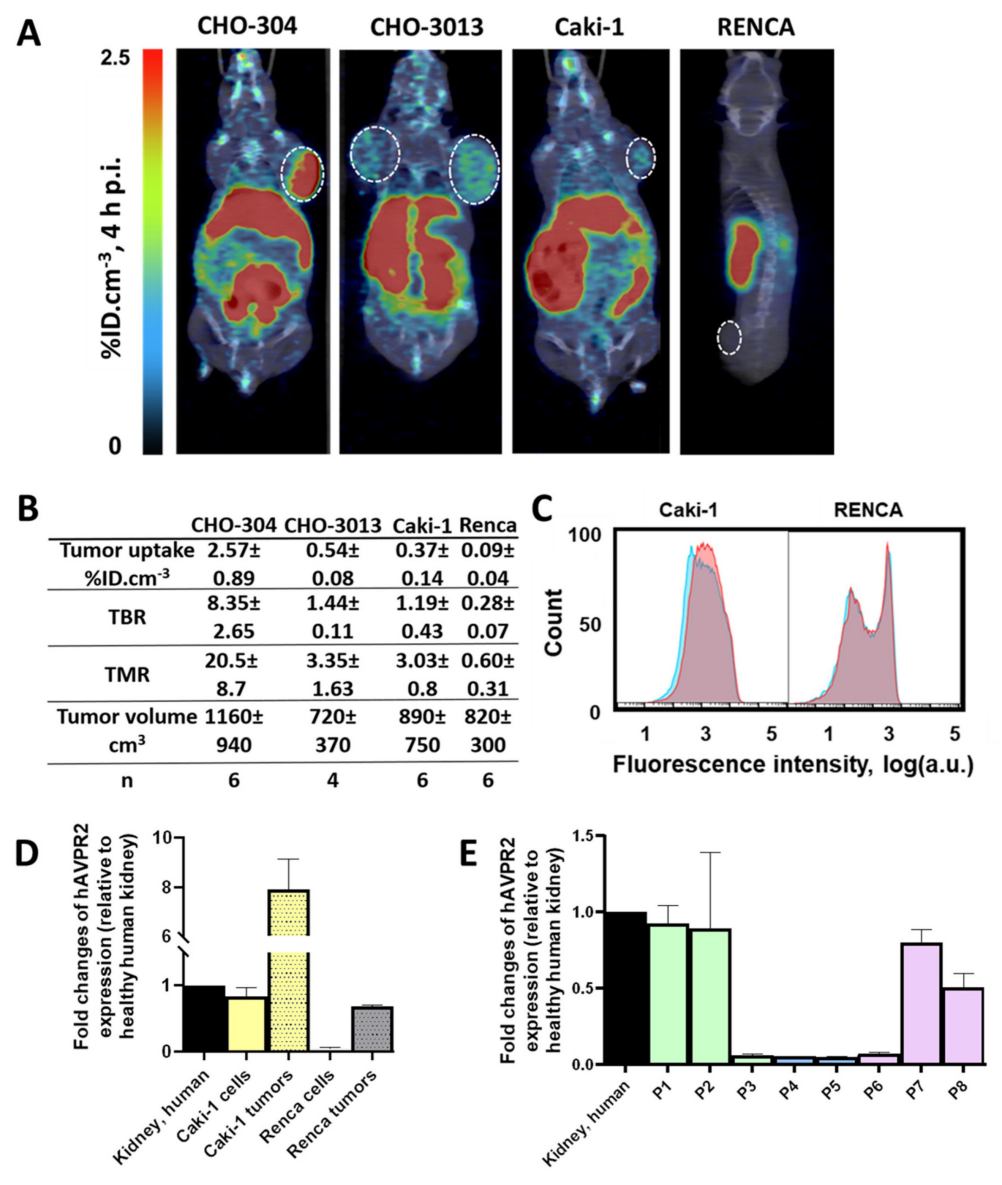
***In vivo* visualization of V2R^+^ tumors in mccRCC representative mice models.** A: Representative composite images taken at 4 h after the i.v. injection of 20 nmol/kg for the [^18^F]F-MQ232 in CHO-304 xenografted NMRI-Foxn1*^nu/nu^* mice (6.8 ± 1.1 MBq, 274 ± 43 MBq/kg), CHO-3013 xenografted NMRI-Foxn1^nu/nu^ mice (3.8 ± 1.2 MBq, 150 ± 47 MBq/kg), Caki-1 xenografted NMRI-Foxn1*^nu/nu^* mice (3.8 ± 1.2 MBq, 152 ± 47 MBq/kg) and Renca grafted BALB/cJ mice (2.5 ± 0.9 MBq, 99 ± 37 MBq/kg). Images shown are the result of a fusion between the Computed Tomography (CT) image (grey scale) and the PET image (false colors). B: Summary table of the main PET imaging indicators obtained after 4 hours of the i.v. injection of 20 nmol/kg for the [^18^F]F-MQ232 in CHO-304 xenografted NMRI-Foxn1^nu/nu^ mice (6.8 ± 1.1 MBq, 274 ± 43 MBq/kg), CHO-3013 xenografted NMRI-Foxn1*^nu/nu^* mice (3.8 ± 1.2 MBq, 150 ± 47 MBq/kg), Caki-1 xenografted NMRI-Foxn1*^nu/nu^* mice (3.8 ± 1.2 MBq, 152 ± 47 MBq/kg) and Renca grafted BALB/cJ mice (2.5 ± 0.9 MBq, 99 ± 37 MBq/kg). C: Flow cytometry results obtained on freshly dissociated Caki-1 and Renca cells stained using 100 nM Cy5-MQ232 (total signal, red curve) or 100 nM Cy5-MQ232 in presence of 30 µM of MQ232 (non-specific signal, blue curve). Light gathered at 680 nm, 30,000 events per acquisition, two acquisitions per independent experiment, *n* = 3). D: Quantification results of *AVPR2*-directed RT-qPCR performed on Caki-1 and Renca cells and tumors resulting from their implantation. Results are shown as fold changes between the expression in the investigated samples and the expression in the positive reference, healthy human total kidney. E: Quantification results of *AVPR2*-directed RT-qPCR performed on 8 ccRCC and mccRCC biopsies (P1 to P8). Three samples came from low grade primary tumors (grade ≤ 2) (P1 to P3, in green), two from high grade primary tumors (grade 3 or 4) (P4 and P5, in blue), and three from distant metastases (P6 to P8, in purple). Results are shown as fold changes between the expression in the investigated samples and the expression in the positive reference, healthy human total kidney.

To assess the imaging capabilities of the radioligand under more physiologically relevant V2R expression conditions, we tested it on CHO-3013 tumor bearing mice (*n* = 6) with an *hAVPR2* expression close to the canonic one (**Fig 2A**). [^18^F]F-MQ232 was injected in these tumor bearing mice (*n* = 4 to 6) and radioactivity quantification was done at t + 4 h p.i. (**Figure S12**). A tumor uptake of 0.54 ± 0.08%ID.cm^-3^ was observed with favorable TMR and TBR of 3.35 ± 1.63 and 1.44 ± 0.11 at t + 4 h, respectively (**Figure 6A-B**).

As our final proof of concept, we evaluated the radioligand in clinically relevant models of mccRCC. Four immortalized human cell lines were known to express the *hAVPR2* gene as well as the V2R protein thanks to a previous study 23: Caki-1, Caki-2, A498 and ACHN. We further investigated the Caki-1 cell line, the only immortalized line derived from human mccRCC 32,33. A second cell line called Renca, originating from a murine RCC, was also investigated as a V2R-negative control to initiate a first evaluation of potential immune system and EPR roles in [^18^F]F-MQ232 tumor uptake in xenografted mice.

Flow cytometry revealed a specific binding-associated fluorescence signal of 630 ± 350 on Caki-1 cells (**Figure 6C**), confirming the presence of a targetable pool of V2R, albeit weaker than that observed in CHO-3013 cells. Renca cells express neither *mAVPR2* nor V2R using the same techniques (**Figure 6C-D, Table S3**). [^18^F]F-MQ232 was injected into corresponding xenografted mice (*n* = 6 each), and radioactivity quantification was performed at t + 4 h p.i. (**Figure 6A-B, Figure S12,18, Table S12**). We obtained a perfect correlation between *AVPR2* expression and tumor uptakes as well as TBR and TMR. Renca tumor uptake with TBR and TMR below unit confirms that no EPR effect is contributing to the signal observed in V2R-positive tumor models, as well as the requirement of V2R expression for tumor labeling. In excised Caki-1 tumors, *hAVPR2* expression increased sevenfold compared to the cells' baseline expression demonstrating the interest of this tumor model for the evaluation of our radioligand. A similar pattern was observed in Renca tumors, where *hAVPR2* expression reached 68% of the level found in human kidney tissue (**Figure 6D, Table S6**).

### Translation to human

We analyzed data from the Human Protein Atlas to assess *AVPR2* expression in human RCC of all etiologies as well as in ccRCC, revealing clear heterogeneity. *AVPR2* displays one of its highest expression levels in kidney tissue under physiological conditions (mean nTPM ≈ 10.7 in normal kidney, compared to < 3 in most other organs), consistent with its canonical role in water homeostasis. Here we performed RT-qPCR analysis on eight human ccRCC biopsy samples to evaluate *AVPR2* expression intensity (**Figure 6E, Table S13**). Three samples were from low-grade primary tumors (grade ≤ 2), two from high-grade primary tumors (grade 3 or 4) and three from distant metastases. We found expression heterogeneity: four samples expressed the gene at a similar level than healthy human kidney and Caki-1 cells while the four others had a near-zero expression of the target gene (**Figure 6E**). We acknowledge that our human tissue cohort is limited and that we only evaluated mRNA expression.

## Discussion and Conclusion

The diagnosis of mccRCC remains notoriously complex, primarily due to the absence of specific imaging tools capable of precisely and comprehensively assessing the lesions' extension, especially in their early stages. This challenge is compounded by the high heterogeneity of these tumors and their often-low metabolic activity, which significantly hinders the imaging capabilities of FDG. As a result, FDG is not recommended for mccRCC imaging. While there are ongoing developments in PET radioligands targeting ccRCC, namely those that target tumor neovasculature (PSMA-based tracers) and carbonic anhydrase IX for primary tumor staging (CAIX-targeting, girentuximab-based tracers), these lack specificity for mccRCC cells 34,35. A major limitation in precise mccRCC diagnosis and imaging lies in the absence of ligands that specifically bind to mccRCC cells regardless of the biological compartment in which they develop or the lesion's stage of progression. To address this, a new ligand specifically targeting mccRCC and compatible with the development of a functional radioligand suitable for PET imaging would be required, enabling effective and early metastases detection.

In this context, V2R emerges as a promising candidate biomarker for mccRCC. V2R, a kidney-specific receptor, is also expressed by a variety of cancer cells including several renal cell carcinoma (RCC) lines, as demonstrated in previous studies 19-21. The Human Protein Atlas database has also provided valuable insight into the expression of *AVPR2* in human RCC, revealing significant heterogeneity even within ccRCC-specific cohorts. Our validation of *AVPR2* expression at the RNA level in 50% of the biopsies tested -representing different grades of ccRCC and mccRCC- suggests a potential role for V2R as a target for diagnostic imaging. Unfortunately, the biopsy preservation conditions were not compatible with Cy5-MQ232 imaging which prevented the validation of V2R expression at the protein level. It is worth noting that a second isoform of V2R (isoform 2), which lacks the seventh transmembrane domain due to a point mutation in the genomic sequence, has been shown to be expressed by some RCC cell lines, including Caki-1. It has been hypothesized that ccRCC tumors and their metastases exhibit stage-correlated upregulation of this isoform 36. These findings warrant further investigation of V2R isoforms in Caki-1 cells and ccRCC biopsies.

Several types of ligands are known to bind to canonical V2R: vasopressin, vaptans, poly- and monoclonal antibodies, and mambaquaretins. Vaptans are small chemical molecules that are difficult to modify without losing their functionality and have not been described as potential PET radioligands over the past two decades of research. GPCR-directed antibodies, such as those targeting V2R, are notoriously challenging to develop and are not suitable for *in vivo* use. In contrast, nature has evolved highly selective peptide toxins, such as mambaquaretins from the venom of mamba snakes, which can rapidly reach their targets *in vivo* with extraordinary selectivity 23,31. MQ232, derived from such a toxin, represents a breakthrough in this field and shows no toxicity at imaging-compatible doses 28.

In our study, we successfully performed *in vivo* imaging of ectopically expressed V2R for the first time. This achievement was made possible by the development of new MQ232-based molecular probes which offer significant potential for V2R investigation. The distinctive biology of GPCRs presents substantial obstacles for research and development, hindering their cancer-related study. Developing high-quality, specific anti-GPCR antibodies represents a major challenge 37. The fluorescent probe Cy5-MQ232 bypasses these obstacles and provides a highly specific tool for the detection of V2R. Used in a similar manner as a fluorescent antibody, it allows a variety of fluorescence-based detection and quantification methods. This approach, which we term “*toxinofluorescence*,” offers a new alternative to traditional immunofluorescence techniques. Moreover, this probe is versatile enough to be applied *in vivo* for whole-body fluorescence imaging in rodent models. To our knowledge, this is the first successful demonstration of a V2R-targeted selective probe allowing imaging of this receptor in a living organism, providing a promising method for preliminary preclinical studies without the use of radioactive compounds.

Following our optical imaging studies, we validated V2R presence by visualizing its ectopic expression in both CHO-derived cells and an mccRCC cell line using PET/CT with the radioligand [^18^F]F-MQ232. [^18^F]F-MQ232 demonstrated its ability to allow specific detection and visualization of V2R expressing tumor compartments, with high tumor uptake observed in the Caki-1-based mccRCC model. The favorable uptake ratios unequivocally confirming tumor localization, combined with the low production cost of this radioligand, strongly support further development of this new imaging modality for both preclinical and, potentially, clinical applications. Moreover, the correlation between V2R levels and [^18^F]F-MQ232 uptake reinforces the potential of this radioligand as a powerful diagnostic tool for mccRCC and other V2R-expressing tumors.

A crucial aspect of *in vivo* radioligand characterization is the validation of ligand-receptor selectivity. In our study and thanks to a deep understanding of the interaction between wild-type mambaquaretins and V2R 31, we addressed this challenge by developing MQ-IMPAIRED, an innovative tool designed to study the *in vivo* selectivity of MQ232 and MQ232-based molecular probes. MQ-IMPAIRED shares the same physicochemical and pharmacokinetic properties as MQ232, enabling us to investigate the MQ232/V2R interaction *in vivo* without requiring large quantities of unlabeled molecules, which could otherwise lead to toxicity-linked issues. This interaction-free peptide approach represents a promising method for studying selectivity in living organisms, and we believe it could be highly beneficial for future applications involving peptide-based imaging probes.

Finally, it is worth noting that thousands of animal toxins targeting membrane proteins with high affinity and selectivity have been described in the literature. These toxin-membrane protein couples present untapped potential for developing new diagnostic tools, similar to the success seen with chlorotoxin, a 36-amino-acid peptide from the venom of *Leiurus quinquestriatus*, the deathstalker scorpion. Chlorotoxin has led to the development of several imaging tools, some of which have already been introduced into clinical practice 38-40. This underscores the vast potential of toxin-based strategies in the development of highly selective diagnostic tools for cancers and other diseases.

While comparative PET imaging using [¹⁸F]FDG or CAIX-targeted tracers could further contextualize our results, such tracers do not adequately reflect the biological heterogeneity and metastatic behavior of ccRCC. [¹⁸F]FDG PET, which measures glucose metabolism, has demonstrated poor sensitivity for renal lesions and a high false-negative rate in metastatic ccRCC due to the tumor's variable glycolytic activity 41-42. Similarly, CAIX-targeted agents such as radiolabeled Girentuximab primarily visualize primary tumor sites where CAIX expression is maintained but fail to detect a large fraction of metastases where CAIX expression is reduced or heterogeneous 43. In contrast, [¹⁸F]F-MQ232 specifically targets the ectopic expression of V2R observed in metastatic ccRCC cells, thereby addressing a key unmet need for accurate identification of disseminated lesions.

In addition to CAIX and PSMA, CD70 has recently emerged as a potential target for molecular imaging and therapy in renal cell carcinoma. CD70 is overexpressed in approximately 50-60% of metastatic ccRCCs, and several studies have investigated radiolabeled anti-CD70 immunoPET strategy 44. In this context, the V2R-targeted strategy described here offers a complementary biological mechanism, focusing on ectopic receptor expression rather than cell-surface antigen targeting.

In our study we observed elevated *AVPR2* mRNA expression in ccRCC samples. However, considering publicly available data from the Human Protein Atlas, it should be emphasized that *AVPR2* does not appear to be uniquely over-expressed in ccRCC, and expression across multiple tumor types is observable, which can enlarge the application of such ligand for several cancer types 45. It highlights its potential as a versatile biomarker for molecular imaging across multiple tumor types, which will be evaluated in a different study.

In conclusion, the successful development and application of V2R-targeting imaging tools, particularly those using nature-derived toxins like MQ232, represent a significant advancement in the field of cancer diagnosis. This approach opens new perspectives for non-invasive and highly specific imaging techniques, offering the potential for better diagnosis and monitoring of mccRCC and other V2R-associated diseases. Furthermore, the exploration of other toxin/membrane protein interactions offers exciting possibilities for the development of new diagnostic and therapeutic modalities that could revolutionize clinical practice in oncology and nephrology.

## Supplementary Material

The Supplementary Information includes detailed chemical synthesis procedures, radiochemistry protocols, RT-qPCR datasets, flow cytometry analyses, *in vivo* imaging quantification, PET pharmacokinetic data, and additional figures and tables supporting the main findings. All datasets are available within the Supplementary Information. Further materials or data can be provided by the corresponding authors upon reasonable request if needed.

## Data Availability

All datasets generated and analyzed in this study are provided in the Supplementary Information. Additional data can be made available by the corresponding authors on reasonable request if needed.

## Abbreviations

3D-OP-OSEM: Three Dimensional-Ordinary Poisson-Ordered Subset Esperance Maximization
ATCC: American Type Culture Collection
a.u.: arbitrary unit
AUC: Area Under the Curve
AVP: Arginine Vasopressin Peptide
*AVPR1A*: Arginine Vasopressin Receptor 1a (gene)
*AVPR1B*: Arginine Vasopressin Receptor 1b (gene)
*AVPR2*: Arginine Vasopressin Receptor 2 (gene)
*B2M*: Beta 2 microglobulin (gene)
Bq: Becquerel
BSA: Bovine Serum Albumin
CA9: Carbonic Anhydrase 9 (protein)
*CAIX*: Carbonic Anhydrase IX (gene)
ccRCC: clear cell Renal Cell Carcinoma
CHO: Chinese Hamster Ovary
Cy5: Cyanine 5
Da: Dalton
DAPI: 4',6-DiAmidino-2-PhénylIndole
DBCO: Dibenzocyclooctyne
dDAVP: Desmopressin
DFO: Deferoxamine
DMEM: Dulbecco's Modified Eagle Medium
DMF: Dimethylformamide
DMSO: Dimethylsulfoxyde
DNA: Desoxyribonucleic acid
DPBS: Dulbecco's Phosphate Buffer Saline
EDTA: Ethylenediaminetetraacetic Acid
EM: Esperance Maximization
EPR: Enhanced Permeability and Retention
FDG: Fluorodeoxyglucose
Fmoc: Fluorenylmethoxycarbonyl
GPCR: G Protein Coupled Receptor
HATU: 1-[Bis(dimethylamino)methylene]-1H-1,2,3-triazolo[4,5-b]pyridinium 3-oxyde hexafluorophosphate
HCTU: O-(1H-6-Chlorobenzotriazole-1-yl)-1,1,3,3-tetramethyluronium hexafluorophosphate
HEPES: 4-(2-hydroxyethyl)-1-piperazine ethane sulfonic acid
RP-HPLC: Reverse Phase High Performance Liquid Chromatography
hV2R: human form of V2R
ID: Injected Dose
IR: Infrared
iTLC: instant Thin Layer Chromatography
i.v.: intravenous
Kd: Dissociation constant
LC-MS: Liquid Chromatography-Mass Spectrometry
nm: nanometer
MAP: Maximum A Posteriori
MBq: Megabecquerel
mccRCC: metastatic ccRCC
MIP: Maximum Intensity Projection
mM: millimolar
MQ: Mambaquaretin
mRNA: messenger RNA
ms: millisecond
NEAA: Non Essential Amino Acid
nM: nanomolar
OP-OSEM: Ordinary Poisson OSEM
OSEM: Ordered Subset Esperance Maximization
OXTR: Oxytocin Receptor (protein)
PBS: Phosphate Buffer Saline
PCR: Polymerase Chain Reaction
PET: Positron Emission Tomography
PFA: Paraformaldehyde
p.i.: post injection
pM: picomolar
PSMA: Prostate Specific Membrane Antigen
qPCR: quantitative PCR
RCC: Renal Cell Carcinoma
RFU: Relative Fluorescence Unit
RNA: Ribonucleic acid
RPM: Rotation Per Minute
RPMI: Roswell Park Memorial Institute
RT: Reverse Transcription
rV2R: rat form of V2R
SPECT: Single Photon Emission Computed Tomography
TAC: Time-Activity Curve
TCC: Transitional Cell Carcinoma
TIPS: Triisopropylsilane
TFA: Trifluoroacetic acid
TNM: Tumor, Node, Metastasis
µg: microgram
µL: microliter
µM: micromolar
V1aR: Vasopressin receptor 1a (protein)
V1bR: Vasopressin receptor 1b (protein)
V2R: Vasopressin receptor 2 (protein)
VOI: Volume Of Interest
