# Supplementary material for: Specific in vivo detection of V2R-positive metastatic ccRCC using a toxin-based PET radioligand
Source: Theranostics. 2026 Feb 4;16(9):4471–88. doi: 10.7150/thno.126311 (PMC12964010; doi:10.7150/thno.126311)
Supplement: Supplementary file 1 — The Supplementary Information includes detailed chemical synthesis procedures, radiochemistry protocols, RT-qPCR datasets, flow cytometry analyses, in vivo imaging quantification, PET pharmacokinetic data, and additional figures and tables supporting the main findings. All datasets are available within the Supplementary Information. Further materials or data can be provided by the corresponding authors upon reasonable request if needed. [file thnov16p4471s1.pdf]

Supplementary Material

1 Supplementary Methods

1.1 Radiochemistry

Reaction with [^{19}F]F-DBCO

In order to check the formation of the expected conjugates F-DBCO-MQ232 and F-DBCO-MQ.IMPAIRED, the strain-promoted azide alkyne cycloaddition was performed with cold [^{19}F]F-DBCO (**Supplementary Figure 1**). A [^{19}F]F-DBCO DMF solution (10 mg/mL, 5 eq.) was added to a solution of N_3-MQ232 or N_3-MQ.IMPAIRED (200 µg) in PBS (150 µL). After 3 hours at 25 °C, the conjugated toxins were purified by Minitrap-G-25 and analyzed by LC-MS.

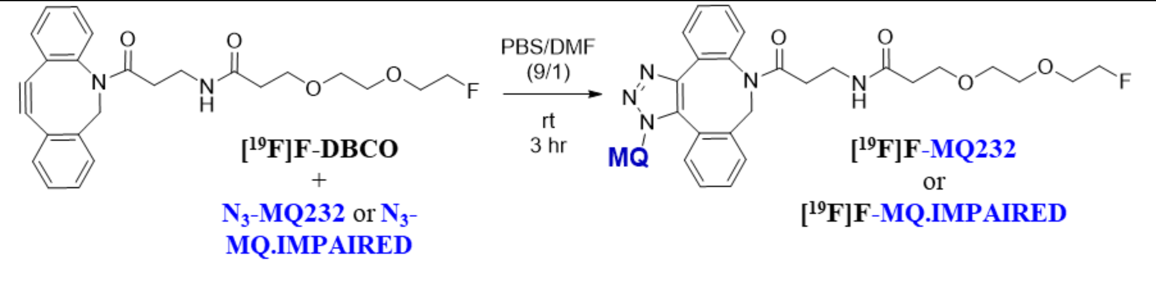

Supplementary Figure 1: Labeling of MQ232 and MQ.IMPAIRED with [^{19}F]F-DBCO

Radiolabeling of MQ232 and MQ.IMPAIRED with [^{18}F]F-DBCO

[^{18}F]F-DBCO was first prepared on an AllInOne automate and then conjugated to N_3-MQ232 and N_3-MQ.IMPAIRED (**SF2**).

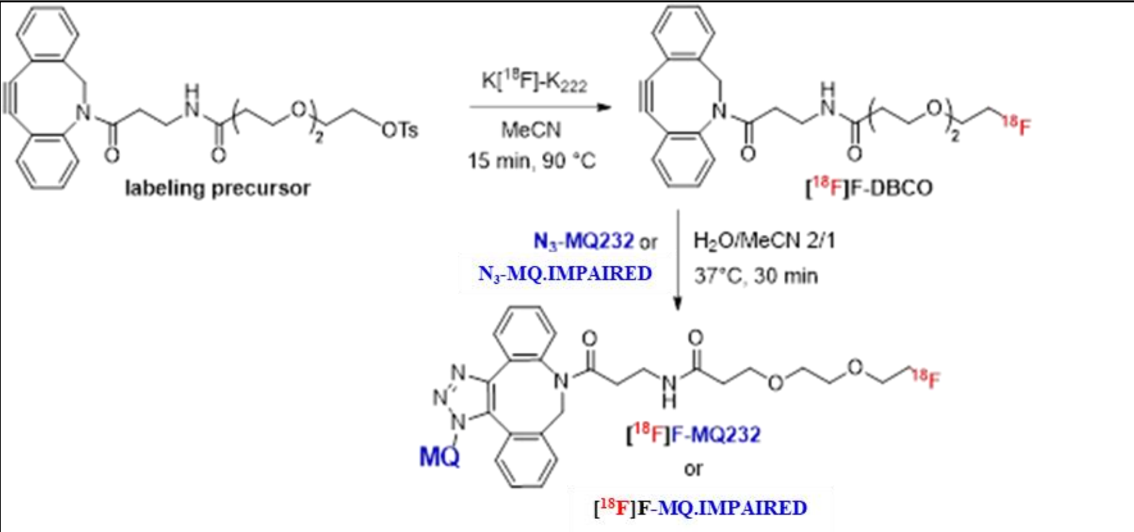

Supplementary Figure 2: Conjugation of MQ232 and MQ.IMPAIRED with [^{18}F]F-DBCO.

1.2 Analytical HPLC

Analytical HPLC analyses were performed on a Waters Alliance (e2695) equipped with a UV spectrophotometer (Waters 2489) and a radioactivity detector (Berthold LB500 fLumo);

analytical column: Symmetry-M® C-18, 50 x 4.6 mm, 5 µm (Waters); eluent A: H_2O containing low-UV PIC® B7 reagent (20 mL by 1000 mL), eluent B: H_2O / CH_3CN: 30:70 (v/v) containing low-UV PIC® B7 reagent (20 mL by 1000 mL), flow rate: 2.0 mL/min; 13 minutes run with the following gradient: 5% eluent B for 1 min, from 5% to 100% eluent B in 10 minutes, from 100% to 5% eluent B in 3 minutes, U.V. detection at λ = 220 nm.

Radiolabeling

Synthesis of the labeling precursor DBCO-OTs and radiosynthesis of the prosthetic group [^{18}F]F-DBCO were adapted from Richard *et al*., 2019. No carrier-added aqueous [^{18}F]F fluoride ion was produced *via* the [^{18}O(p,n)^{18}F] nuclear reaction by the irradiation of [^{18}O]water (2mL) target (> 97%-enriched, CortecNet) on a Cyclone-18/9 cyclotron (18 MeV proton beam, IBA) and was transferred to the hot cell. Target hardware: commercial, 2-mL, two-port, stainless steel target holder, which is equipped with a domed-end niobium cylinder insert. The target to hot cell liquid-transfer system: 60 m PTFE line (0.8 mm internal diameter; 1/16 inch external diameter), 2.0 bar helium drive pressure, transfer time 3-6 min. Typical production of [^{18}F]F fluoride ion at the end of bombardment for a 25 µA, 30 minutes irradiation: 27-30 GBq. The aqueous solution containing [^{18}F]F fluoride anions was automatically transferred to the Trasis All-in-One automate after the end of irradiation. The irradiated water was then passed through an anion exchange cartridge (Sep-Pak® Accell Plus QMA Plus Light cartridge, Waters) to fix [^{18}F]F fluoride anions and remove the enriched water. The [^{18}F]F fluoride anions were eluted from the resin and transferred to the reactor with 300 µL of a solution containing 10 mg of Kryptofix® 222 (K_{222}) in 450 µL of acetonitrile and 100 µL of 33.3 mg/mL K_2CO_3. The K[^{18}F]F-K_{222} complex was prepared by evaporation of the solution under vacuum and under a stream of helium at (i) 80 °C for 15 s, (ii) 115 °C for 115 s, (iii) 125 °C for 180 s, (iv) 110 °C for 80 s and (v) 95 °C for 100 s. After cooling to 40 °C, the labeling precursor was added in 700 µL MeCN and the radiolabeling was carried out at 90 °C for 15 minutes before 2 successive dilutions with water (5 mL and 3.5 mL) and semi-preparative HPLC purification (column: Zorbax SB-C18 5 µm 9.4x250 mm (Agilent); H_2O/MeCN/TFA: 65/35/0.1 (v/v/v); flow rate: 5 mL/min; detection wavelength = 254 nm). The retention time of [^{18}F]F-DBCO is around 30 min (**SF3**). [^{18}F]F-DBCO was collected in a vial containing 25 mL water and the final formulation was performed automatically using a Sep-Pak C18 cartridge (Waters). The purified product was recovered after elution with acetonitrile (1.5 mL) with a 28.6% ± 9.8 (n = 20) radiochemical yield (d.c.) after an average reaction time of 70 minutes. The chemical and radiochemical purities were higher than 95% as attested by quality control analytical HPLC (retention time of [^{18}F]F-DBCO = 7.23 min, retention time of reference F-DBCO = 7.37 min, **SF4**). Synthesis of [^{18}F]F-DBCO was achieved with an average decay corrected molar activity of 112.2 ± 54.9 GBq/µmol (n = 13).

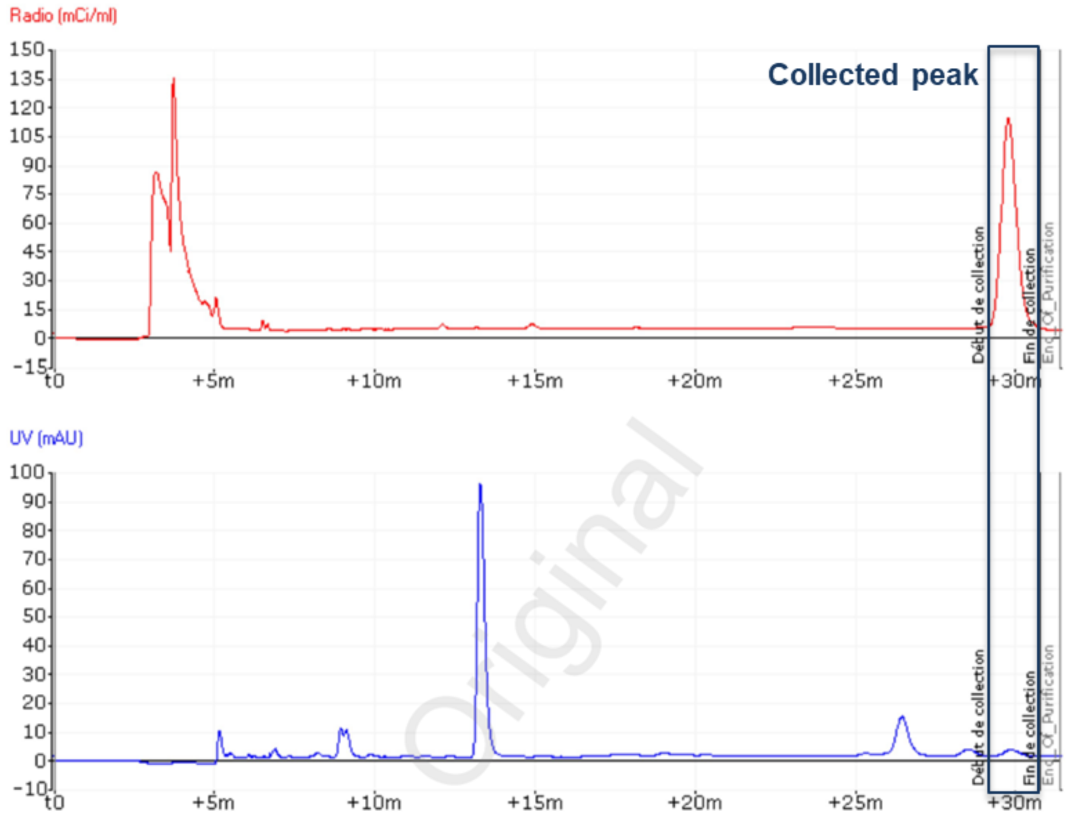

Supplementary Figure 3: Semi preparative HPLC radio and UV chromatograms for [^{18}F]F-DBCO purification

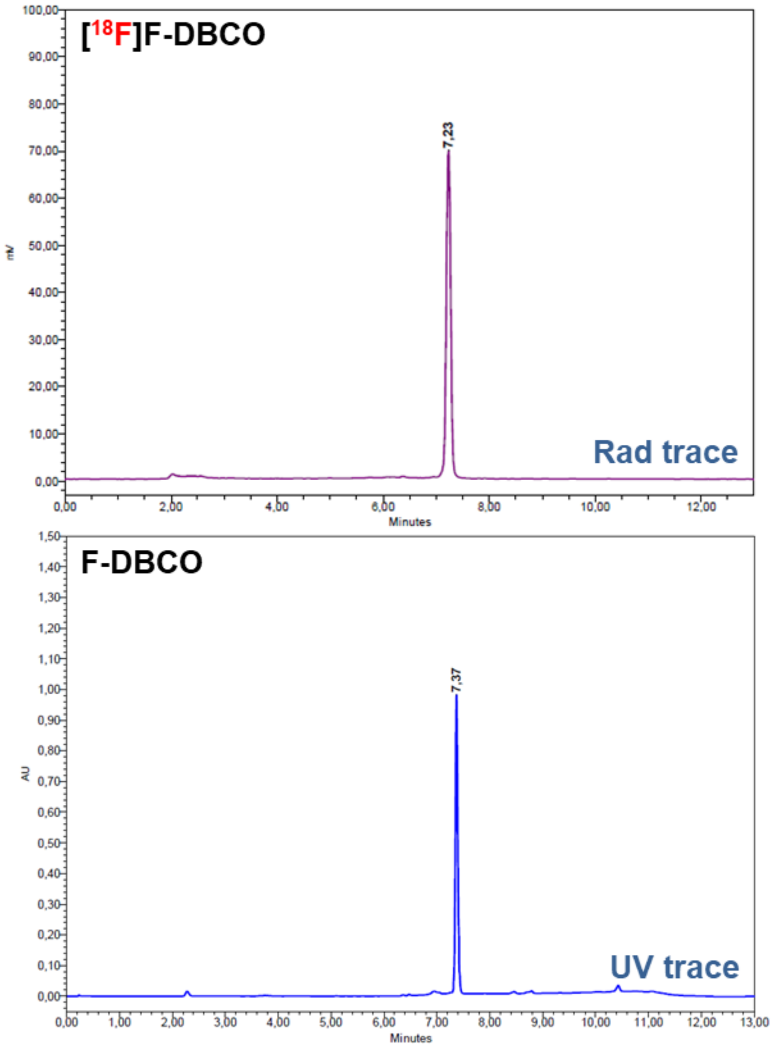

Supplementary Figure 4: Analytical HPLC of purified F-DBCO and [^{18}F]F-DBCO

Radiolabeling of N_3-MQ with [^{18}F]F-DBCO

The radiolabeling was performed manually in a 5-cm-lead shielded glove box. A solution of [^{18}F]F-DBCO in MeCN (1.5-2.0 GBq) was evaporated at 50 °C under a stream of helium for 10 min until volume reached ~ 50 µL. A solution of N_3-MQ232 (100-200 µg in 100 µL water) or N_3-MQ.IMPAIRED (70-100 µg in 100 µL water) was then added to the concentrated [^{18}F]F-DBCO. After incubation at 37 °C for 30 minutes, the crude was purified by size exclusion (minitrap G25, elution in 0.9% NaCl) and the labeled [^{18}F]F-MQ was analyzed by analytical HPLC.

After purification, 184.6 ± 35.9 MBq (n.d.c.) of [^{18}F]F-MQ232 (70-140 µg) was obtained (decay-corrected molar activity: 30.6 ± 6.1 GBq/µmol) (n = 7). The chemical and radiochemical purities were higher than 95% as attested by quality control analytical HPLC (retention time of [^{18}F]F-MQ232 = 5.77 min, retention time of reference [^{19}F]F-MQ232 = 5.78 min, **SF5**).

After purification, between 42-78µg of [^{18}F]F-MQ.IMPAIRED was obtained for an average of 117.2 ± 8.54 MBq (n.d.c.) at 167 min E.O.B. (decay-corrected molar activity: 37.6 ± 7.6 GBq/µmol (n = 3)). The chemical and radiochemical purities were higher than 95% as attested by quality control analytical HPLC (retention time of [^{18}F]F-MQ.IMPAIRED = 5.90 min, **SF6**).

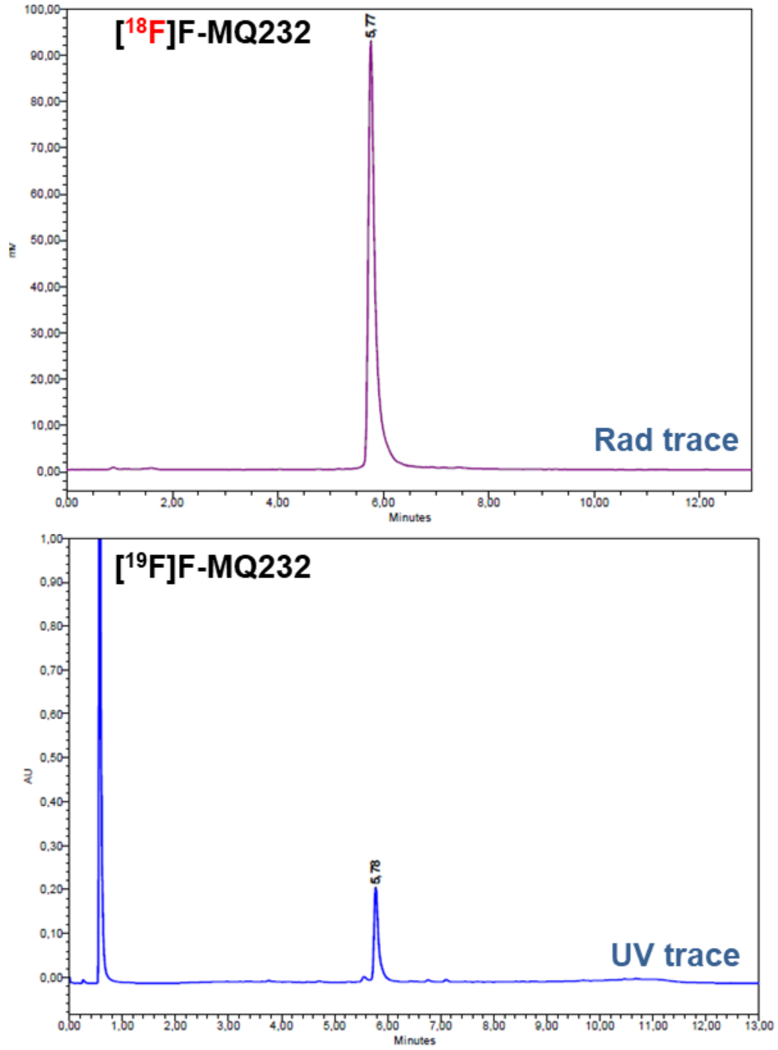

Supplementary Figure 5: Analytical HPLC of purified [^{19}F]F-MQ232 (UV trace) and [^{18}F]F-MQ232 (radioactive trace)

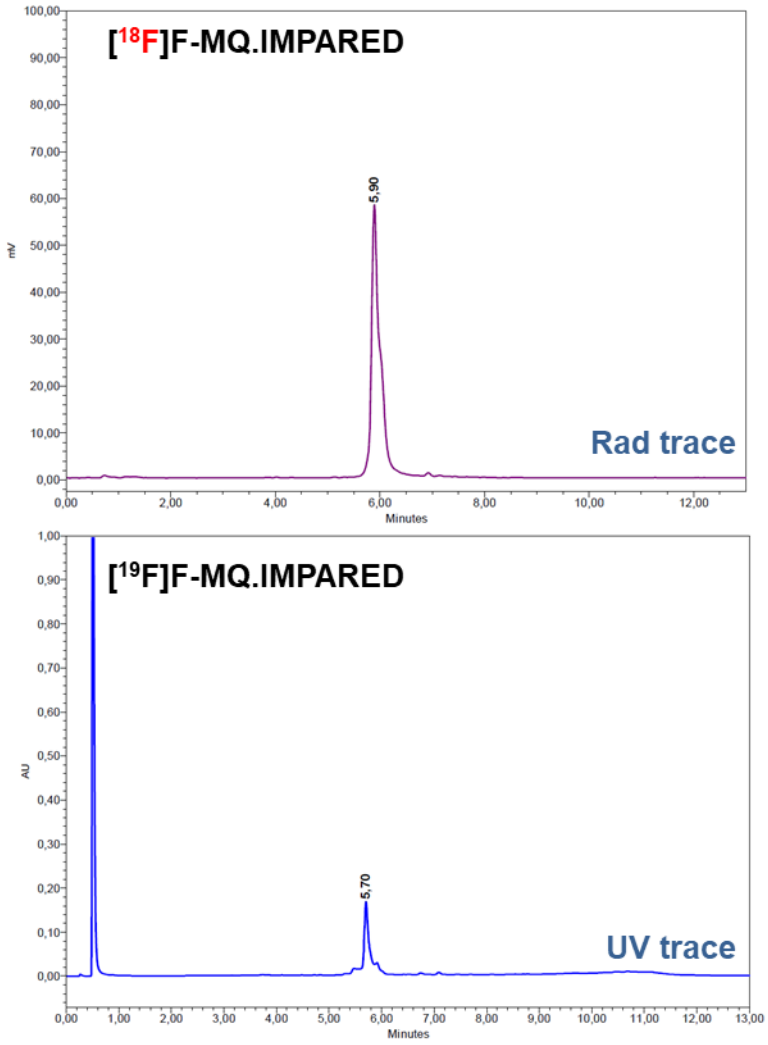

Supplementary Figure 6: Analytical HPLC of purified N_3-MQ.IMPAIRED (UV trace) and [^{18}F]F-MQ.IMPAIRED (radioactive trace)

1.3 In vivo experiment authorizations

Mice PK, PD and imaging experiments:
The animal facility holds an agreement in 2021 for animal experimentation (n°F 91 272 106). All experimental protocols were performed in accordance with 2010/63/EU directive of the European Community as well as the French legislation concerning the protection of laboratory animals, and obtained the authorization APAFIS#32015-2021061612169288.
Mice PET imaging:
Research was approved by a local ethics committee (CETEA, committee n°44) and by the French ministry of agriculture (APAFIS #34522-2022010412087915 v1).

1.4 Data processing and statistical analysis

Quantitative data are shown as means, with error bars indicating the standard error of the mean (SEM). Normality was assessed using the d'Agostino-Pearson test. Comparisons between two groups were performed using the unpaired Student’s t test. Comparisons between more than

two groups were performed using the Kruskal–Wallis test followed by Dunn's test. To compare repeated measurements over time, we used the ANOVA multiple factors followed by Tukey's test. Differences were considered significant if p value was <0.05. Statistical analyses were performed using GraphPad Prism 9.5.1 software. $**** p < 0.0001$, $*** p < 0.001$, $** p < 0.01$, one-way ANOVA followed by Tukey's analysis.

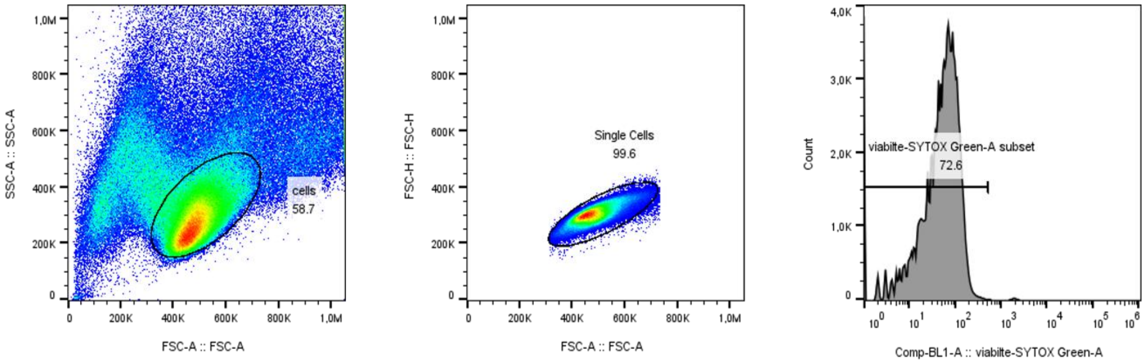

Supplementary Figure 7: Gating strategy used for flow cytometry data analysis

2 Supplementary Figures

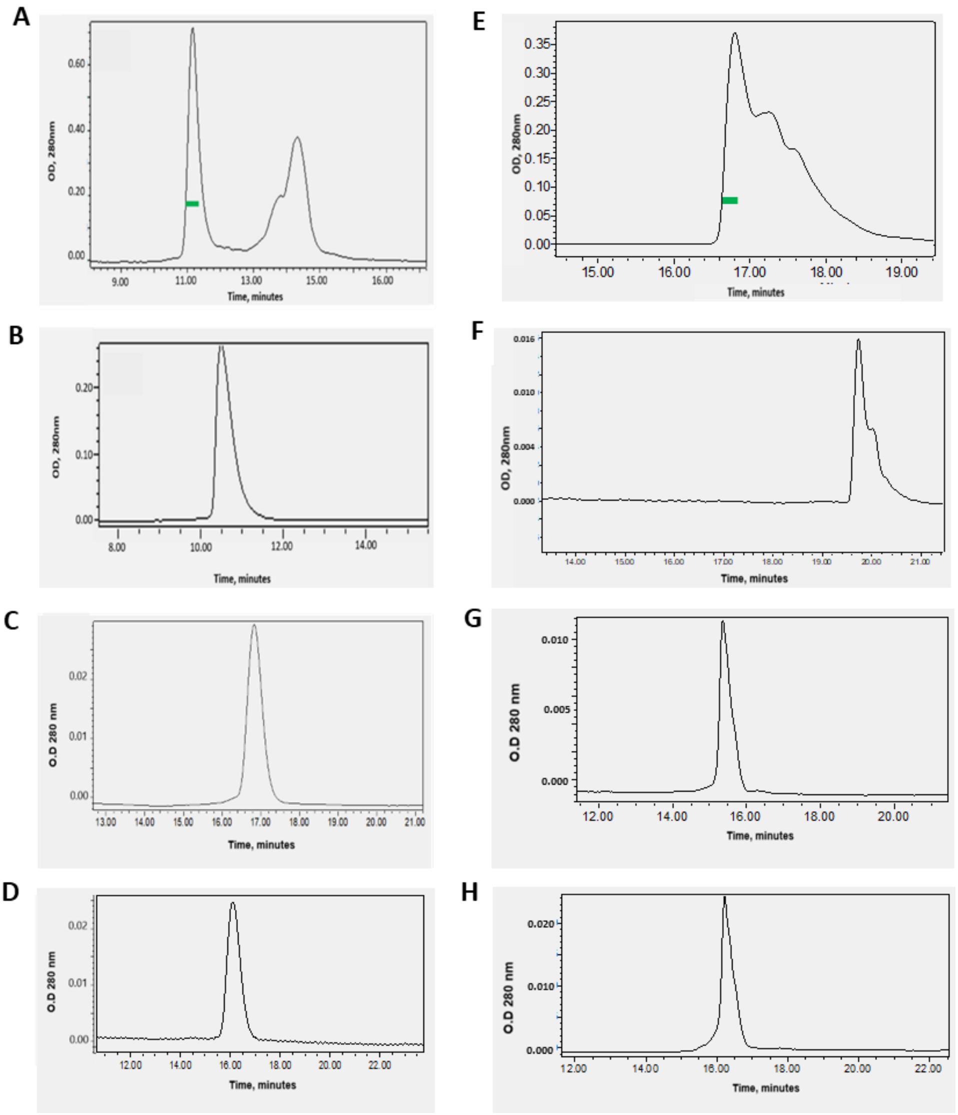

Supplementary Figure 8. Chemical synthesis of MQs. (**A**) MQ232 crude oxidation mixture RP-HPLC profile on a Waters X-bridge C18, 19 × 250 mm, 10 µm column using a flow of 15 mL/min with a 0 to 40% of acetonitrile (ACN) gradient in 40 min. (**B**) Purified and oxidized MQ232 RP-HPLC profile on a Waters Sunfire C18, 10 × 250 mm, 10 µm column using a flow of 5 mL/min and a 15 to 50% CAN gradient in 35 min. (**C**) Analytical RP-HPLC profile of purified Cy5-MQ232 performed on a Waters X-bridge C18, 4.6 × 150 mm, 3.5 µm column using a flow of 1 mL/min and a 20-50% CAN gradient in 30 min. (**D**) Analytical chromatography of purified [^{19}F]F-MQ232 on Waters X-bridge C18, 4.6 × 150 mm, 3.5 µm, same gradient, same flow. (**E**) RP-HPLC profile of crude oxidized MQ.IMPAIRED on a Waters X-bridge Peptide BEH C18, 10 × 250 mm column using a flow of 5 mL/min and a 10 to 40% CAN gradient in 30 min. (**F**) Analytical RP-HPLC profile of oxidized and purified MQ.IMPAIRED on a Waters X-bridge C18, 4.6 × 150 mm, 3.5 µm column using a 1 mL/min flow and the same gradient. (**G**) Analytical RP-HPLC profile of Cy5-MQ.IMPAIRED on a Waters X-bridge C18, 4.6 × 150 mm, 3.5 µm column using a 1 mL/min flow and a 20-50% CAN gradient in 30 min. (**H**) Analytical RP-HPLC profile of purified [^{19}F]F-MQ.IMPAIRED on a Waters X-bridge C18, 4.6 × 150 mm, 3.5 µm column using a 1 mL/min flow and a 20-50% CAN gradient in 30 min.

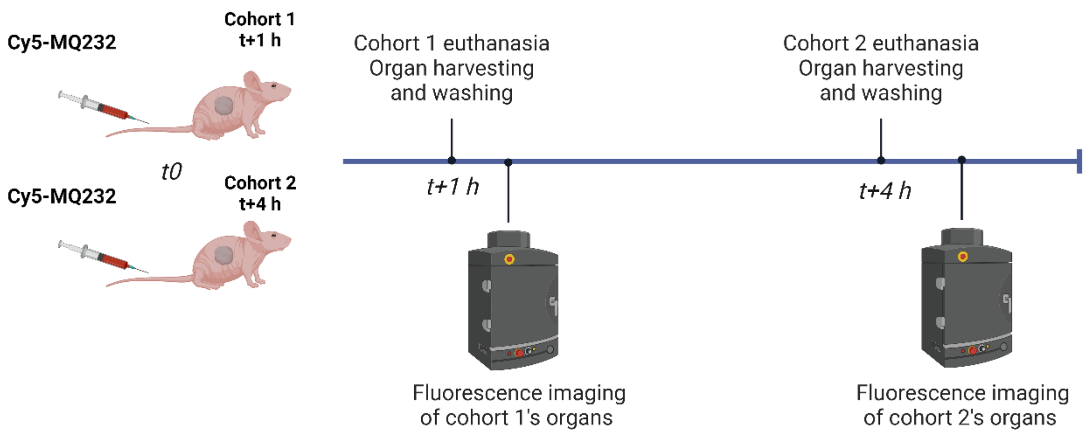

Supplementary Figure 9. Experimental setup for fluorescence imaging of organs at t+1 h and t+4 h after intravenous administration of Cy5-MQ232 (20 nmol/kg).

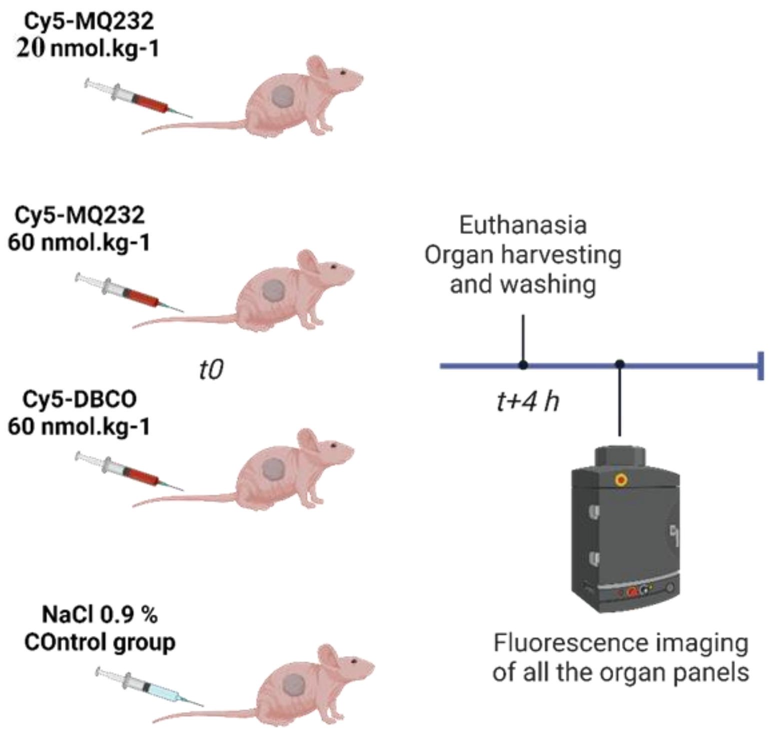

Supplementary Figure 10. Experimental setup for fluorescence imaging of organs at t+4 h after intravenous administration of Cy5-MQ232 (20 or 60 nmol/kg) or Cy5-DBCO (60 nmol/kg) or physiological solution (NaCl 0.9%).

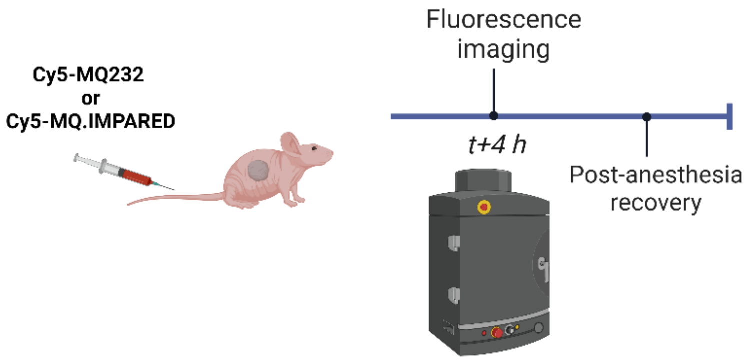

Supplementary Figure 11. Experimental setup for external fluorescence imaging of anesthetized mice intravenously administered with Cy5-MQ232 or Cy5-MQ.IMPAIRED (20 nmol/kg).

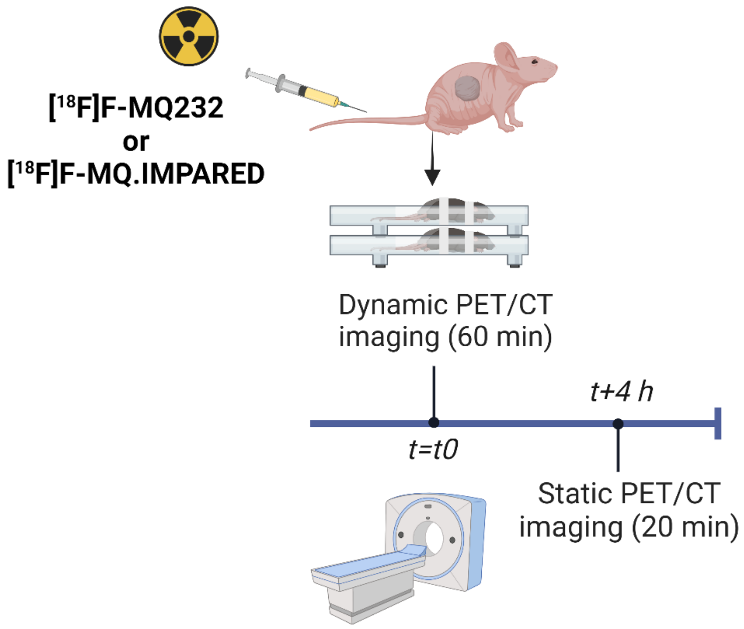

Supplementary Figure 12. Experimental setup for *in vivo* PET/CT imaging in mice intravenously administered with [^{18}F]F-MQ232 or [^{18}F]MQ.IMPAIRED.

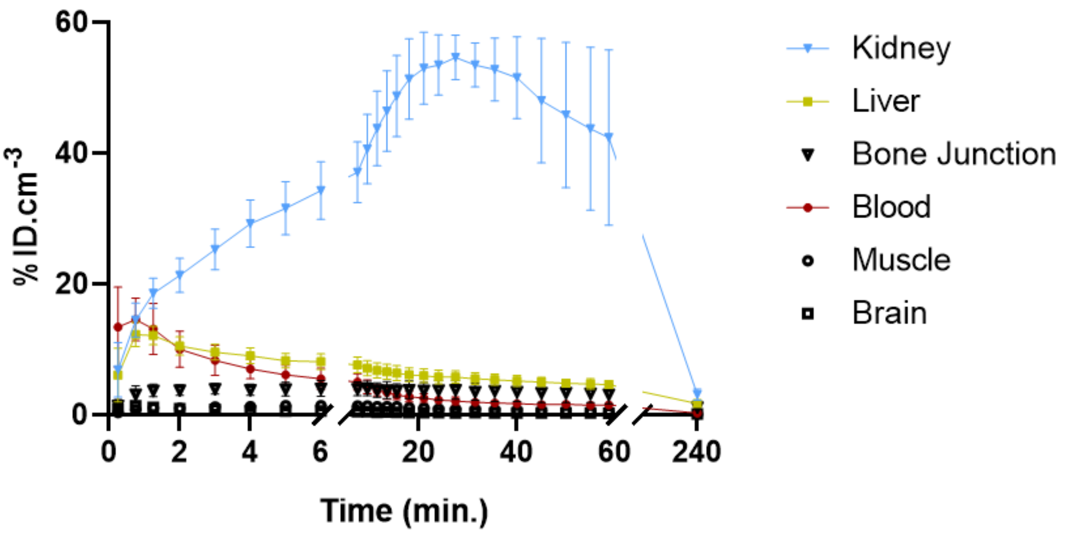

Supplementary Figure 13. Radiotracer's concentration evolution in selected biological compartments of healthy NMRI-Foxn1$^{nu/nu}$ mice after the i.v. injection of 20 nmol/kg [^{18}F]F-MQ232 (3.0 ± 0.7 MBq, 120 ± 28 MBq/kg, *n* = 6, 60-minute dynamic acquisition started at the injection followed by a 20-minute static acquisition at t+4 h).

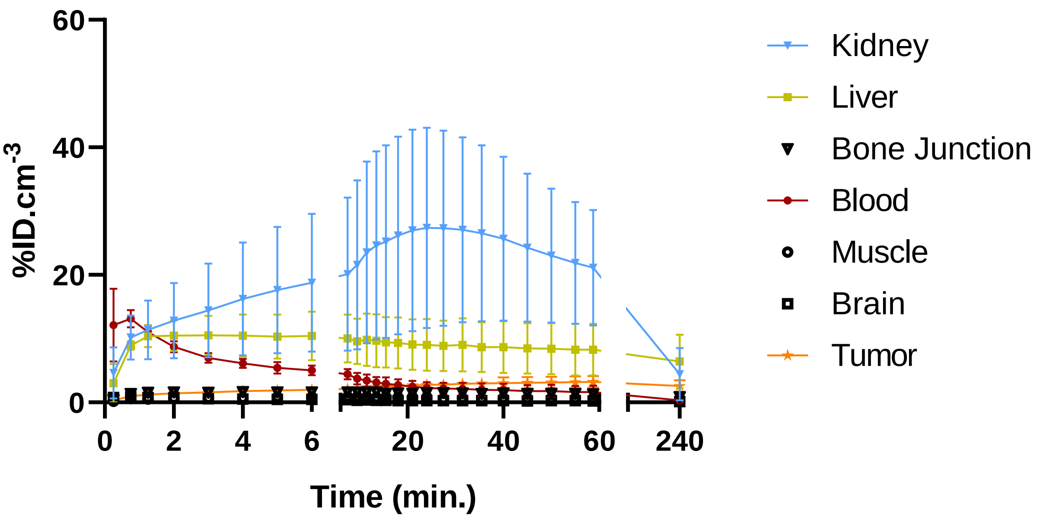

Supplementary Figure 14. Radiotracer's concentration evolution in selected biological compartments of CHO-304 xenografted NMRI-Foxn1$^{nu/nu}$ mice after the i.v. injection of 20 nmol/kg [^{18}F]F-MQ232 (6.8 ± 1.1 MBq, 274 ± 43 MBq/kg, *n* = 6, 60-minute dynamic acquisition started at the injection followed by a 20-minute static acquisition at t+4 h).

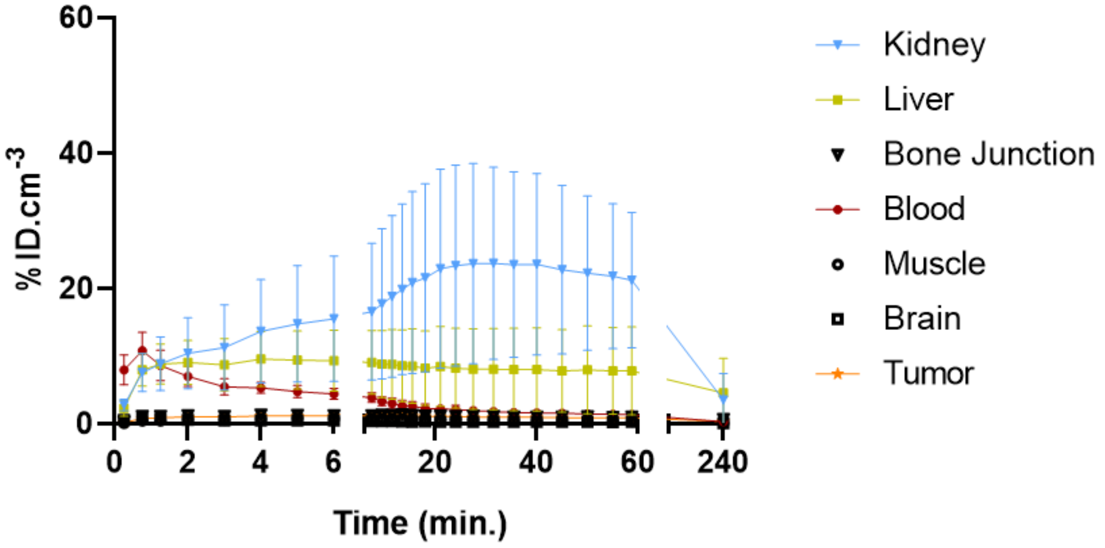

Supplementary Figure 15. Radiotracer's concentration evolution in selected biological compartments of CHO-304 xenografted NMRI-Foxn1$^{nu/nu}$ mice after the i.v. injection of 20 nmol/kg [^{18}F]F-MQ.IMPAIRED (5.8 ± 1.8 MBq, 234 ± 71 MBq/kg, *n* = 6, 60-minute dynamic acquisition started at the injection followed by a 20-minute static acquisition at t+4 h).

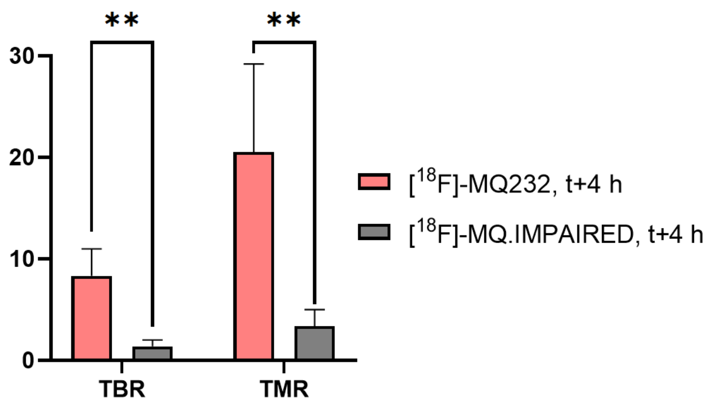

Supplementary Figure 16. Average Tumor-to-Blood (TBR) and Tumor-to-Muscle (TMR) ratios at t+4 h after the i.v. injection of 20 nmol/kg [^{18}F]F-MQ232 (6.8 ± 1.1 MBq, 274 ± 43 MBq/kg, *n* = 6) or of 20 nmol.kg^{-1} [^{18}F]F-MQ.IMPAIRED (5.8 ± 1.8 MBq, 234 ± 71 MBq/kg, *n* = 6). Statistic test: T test. * if p-value < 0,05 ** if p-value < 0,01 *** if p-value < 0,001 **** if p-value < 0,0001

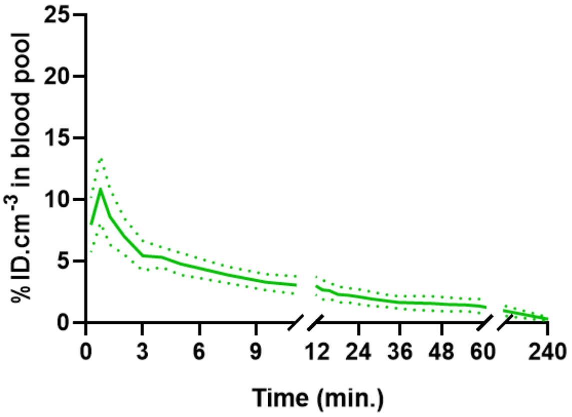

Supplementary Figure 17. Image-derived blood pharmacokinetic profile of [^{18}F]F-MQ.IMPAIRED i.v. injected in healthy NMRI-Foxn1$^{nu/nu}$ mice (2.8 ± 0.8 MBq, 112 ± 32 MBq/kg, *n* = 6)

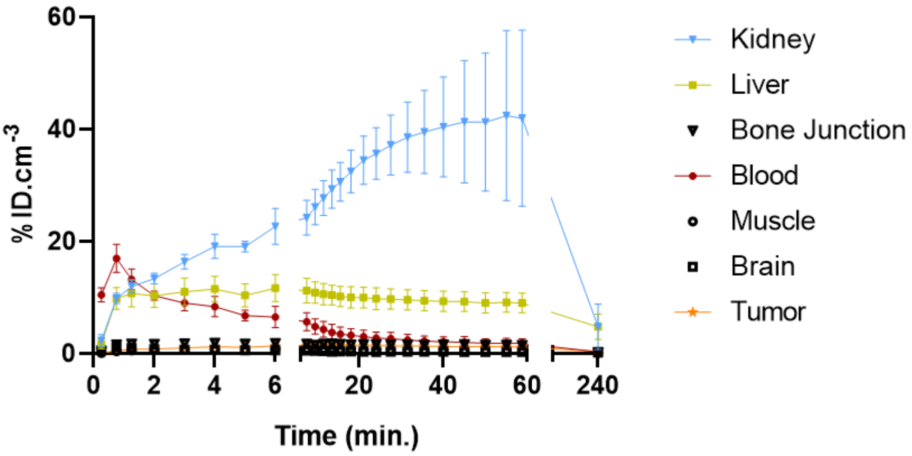

Supplementary Figure 18. Radiotracer's concentration evolution in selected biological compartments of Caki-1 xenografted NMRI-Foxn1$^{nu/nu}$ mice after the i.v. injection of 20 nmol/kg [^{18}F]F-MQ232 (3.8 ± 1.2 MBq, 152 ± 47 MBq/kg, 60-minute dynamic acquisition started at the injection followed by a 20-minute static acquisition at t+4 h).

3 Supplementary Tables

Supplementary Table 1. Diuresis evaluation in rats following the subcutaneous injection of various MQ232-based molecules.

Injected dose (s.c., nmol/kg BW)		30					
Injected molecule		[^{19}F]F-MQ232			Cy5-MQ232		
Animal number		7358	7361	7400	7357	7362	7364
Injected volume (µL)		200	200	190	190	190	190
Parameter	Sampling time						
Weight (g)	H0	203	195	190	186	190	185
Diuresis (mL.h^{-1}.kg^{-1})	Baseline	1.560	1.560	1.560	1.560	1.560	1.560
	H0 to H1	92.798	83.102	78.255	60.249	72.715	58.864
	H1 to H3	111.496	67.867	88.643	49.169	74.792	36.704
	H3 to H5	8.310	7.618	10.388	0.000	8.310	0.000
	H5 to H15	0.973	0.419	0.862	0.579	1.083	0.726

Injected dose (s.c., nmol/kg BW)		30					
Injected molecule		[^{19}F]F-MQ.IMPAIRED			Cy5-MQ.IMPAIRED		
Animal number		7356	7359	7366	7360	7363	7365
Injected volume (µL)		200	200	200	200	190	200
Parameter	Sampling time						
Weight (g)	H0	202	200	195	200	185	195
Diuresis (mL.h^{-1}.kg^{-1})	Baseline	1.560	1.560	1.560	1.560	1.560	1.560
	H0 to H1	0.000	0.000	0.000	0.000	0.000	0.000
	H1 to H3	4.15512	3.463	5.540	0.000	6.925	10.388
	H3 to H5	4.84765	3.463	2.078	5.540	4.155	6.233
	H5 to H15	0.93567	0.899	0.997	0.973	1.034	1.108

Injected molecule		NaCl (0.9%)		
Animal number		7357	7368	7369
Injected volume (µL)		200	200	200
Parameter	Sampling time			
Weight (g)	H0	230	200	200
Diuresis (mL.h^{-1}.kg^{-1})	Baseline	1.560	1.560	1.560
	H0 to H1	1.522	0.217	4.686
	H1 to H3	2.826	4.686	1.522
	H3 to H5	0.217	1.522	2.826
	H5 to H15	4.686	2.826	0.217

Supplementary Table 2. qPCR data obtained on human and murine tissues regarding V2R expression

Sample type	Sample name	Target's species	Mean Ct (V2R)	Mean Ct (Housekeeping gene)
Healthy tissues	Kidney, human	Human V2R	25.31	8.25
	Kidney, mouse	Murine V2R	26.81	8.29
	Cerebrum, mouse	Murine V2R	31.77	9.07

Supplementary Table 3. qPCR data obtained on investigated cell lines regarding V2R expression

Sample type	Sample name	Target's species	Mean Ct (V2R)	Mean Ct (Housekeeping gene)
Cell lines, cultured in vitro	CHO-304	Human V2R	20.44	9.21
	CHO-3013		24.70	9.21
	Caki-1		27.16	10.12
	Renca	Murine V2R	30.84	9.12

Supplementary Table 4. Fluorescence quantification in the organs of healthy NMRI-*Foxn1*$^{nu/nu}$ mice at 1 h and 4 h after the intravenous injection of Cy5-MQ232.

Injected molecule		Cy5-MQ232						
Injected dose (i.v., nmol/kg BW)		20						
Imaging time		t+1 h				t+4 h		
Compartment's mean fluorescence (RFU)	Brain	546	427	508	N.A.	419	342	377
	Heart	296	281	329	402	205	209	226
	Liver	701	571	947	913	296	436	559
	Gallbladder	625	689	N.A.	N.A.	1107	2125	N.A.
	Intestines	759	793	677	811	553	276	448
	Stomach	1914	2127	1575	1798	1719	1245	1570
	Kidney	13677	14042	19120	36057	2821	4961	5457
	Spleen	285	355	283	245	241	264	198
	Muscle	249	269	341	354	221	200	204

Supplementary Table 5. Fluorescence quantification in the organs of CHO-304 xenografted NMRI-*Foxn1*$^{nu/nu}$ mice at t+4 h after intravenous Cy5-MQ232 or Cy5-DBCO or saline (NaCl 0.9%) injection.

Injected molecule		NaCl (0.9%)			Cy5-MQ232			
Injected dose (i.v., nmol/kg BW)					20			
Compartment's mean fluorescence (RFU)	Brain	313	325	302	342	355	327	500
	Heart	240	246	239	230	227	251	256
	Tumor	348	396	362	7402	5719	7763	6097
	Liver	659	685	602	465	499	413	427
	Gallbladder	631	646	600	2789	3355	N.A.	3120
	Intestines	624	627	603	320	354	567	489
	Stomach	1115	1323	1422	2272	1445	1440	1200
	Kidney	600	614	602	5630	9942	6686	8371
	Spleen	231	267	253	270	339	321	309
	Muscle	246	233	228	238	402	312	332

Injected molecule		Cy5-MQ232				Cy5-DBCO		
Injected dose (i.v., nmol/kg BW)		60				60		
Compartment's mean fluorescence (RFU)	Brain	355	343	574	465	313	325	302
	Heart	218	267	257	233	240	246	239
	Tumor	25638	19515	23684	28226	348	396	362
	Liver	887	1092	N.A.	709	659	685	602
	Gallbladder	10587	6453	8540	17199	631	646	600
	Intestines	1014	2318	2860	4183	624	627	603
	Stomach	1339	1302	2501	1948	1115	1323	1422
	Kidney	6959	11666	19103	11376	600	614	602
	Spleen	314	299	318	356	231	267	253
	Muscle	349	321	449	363	246	233	228

Supplementary Table 6. qPCR data obtained on tumors derived from the investigated cell lines regarding V2R expression

Sample type	Sample name	Target's species	Mean Ct (V2R)	Mean Ct (Housekeeping gene)
Tumors from xenografted cell lines	tCHO-304	Human V2R	18.70	7.66
	tCHO-3013		23.12	9.25
	tCaki-1		22.21	8.12
	tRenca	Murine V2R	27.07	9.45

Supplementary Table 7. Averaged PET-obtained activities in healthy NMRI-*Foxn1*$^{nu/nu}$ mice following i.v. injection of 20 nmol/kg [^{18}F]F-MQ232, by organ.

Kidney						
**Time (s)**	**Acquisition length (s)**	**Average Injected Dose (MBq)**	**Averaged Activity (MBq)**	**SD**	**%ID.cm^{-3}**	**SD**
15.00	0.25		0.22	0.17	6.86	4.18
45.00	0.75		0.44	0.15	14.47	2.62
75.00	1.25		0.55	0.13	18.55	2.30
120.00	2		0.64	0.17	21.32	2.59
180.00	3		0.76	0.20	25.29	3.09
240.00	4		0.87	0.22	29.24	3.62
300.00	5		0.95	0.26	31.56	4.04
360.00	6		1.03	0.27	34.28	4.42
450.00	7.5		1.12	0.31	37.09	4.68
570.00	9.5		1.22	0.34	40.63	5.33
690.00	11.5		1.32	0.37	43.76	5.72
810.00	13.5		1.40	0.40	46.42	6.13
930.00	15.5	**3**	1.47	0.41	48.74	6.22
1080.00	18		1.55	0.43	51.32	6.15
1260.00	21		1.60	0.45	52.96	5.51
1440.00	24		1.62	0.45	53.48	4.62
1650.00	27.5		1.64	0.43	54.64	3.40
1890.00	31.5		1.61	0.43	53.48	3.35
2130.00	35.5		1.58	0.43	52.77	4.80
2400.00	40		1.54	0.43	51.50	6.26
2700.00	45		1.43	0.46	48.03	9.50
3000.00	50		1.36	0.47	45.81	11.10
3300.00	55		1.29	0.48	43.72	12.43
3525.00	58.75		1.25	0.49	42.35	13.45
15600.00	240		0.09	0.02	3.06	0.87

Liver						
**Time (s)**	**Acquisition length (s)**	**Average Injected Dose (MBq)**	**Averaged Activity (MBq)**	**SD**	**%ID.cm^{-3}**	**SD**

15.00	0.25		0.20	0.18	6.06	4.12
45.00	0.75		0.38	0.13	12.25	1.85
75.00	1.25		0.37	0.11	12.13	1.40
120.00	2		0.32	0.11	10.52	1.43
180.00	3		0.29	0.10	9.55	1.23
240.00	4		0.28	0.10	9.04	1.20
300.00	5		0.25	0.09	8.28	1.11
360.00	6		0.25	0.09	8.12	1.20
450.00	7.5		0.23	0.08	7.61	1.19
570.00	9.5		0.22	0.08	7.12	1.15
690.00	11.5		0.21	0.08	6.79	1.16
810.00	13.5		0.20	0.08	6.56	1.15
930.00	15.5	**3**	0.20	0.08	6.38	1.14
1080.00	18		0.19	0.07	6.11	1.08
1260.00	21		0.18	0.07	5.96	1.05
1440.00	24		0.18	0.07	5.79	1.05
1650.00	27.5		0.18	0.07	5.75	0.95
1890.00	31.5		0.17	0.07	5.53	0.93
2130.00	35.5		0.17	0.06	5.36	0.88
2400.00	40		0.16	0.06	5.19	0.85
2700.00	45		0.16	0.06	5.05	0.74
3000.00	50		0.15	0.05	4.85	0.64
3300.00	55		0.15	0.06	4.73	0.79
3525.00	58.75		0.14	0.05	4.61	0.60
15600.00	240		0.05	0.02	1.71	0.26

Gallbladder						
**Time (s)**	**Acquisition length (s)**	**Average Injected Dose (MBq)**	**Averaged Activity (MBq)**	**SD**	**%ID.cm^{-3}**	**SD**
15600.00	240	3	0.42	0.33	13.48	7.61

Bone Junction						
**Time (s)**	**Acquisition length (s)**	**Average Injected Dose (MBq)**	**Averaged Activity (MBq)**	**SD**	**%ID.cm^{-3}**	**SD**
15.00	0.25		0.05	0.04	1.41	0.90
45.00	0.75		0.10	0.07	3.11	1.30
75.00	1.25		0.11	0.05	3.70	0.96

120.00	2		0.12	0.05	3.79	0.82
180.00	3		0.12	0.05	3.98	0.81
240.00	4		0.12	0.05	3.83	0.84
300.00	5		0.12	0.05	3.91	1.07
360.00	6		0.12	0.06	4.00	1.22
450.00	7.5		0.12	0.05	3.99	1.07
570.00	9.5		0.12	0.06	4.04	1.11
690.00	11.5		0.12	0.05	3.78	1.09
810.00	13.5		0.12	0.06	3.77	1.32
930.00	15.5	**3**	0.11	0.05	3.70	1.02
1080.00	18		0.11	0.06	3.81	1.35
1260.00	21		0.11	0.06	3.71	1.19
1440.00	24		0.12	0.07	3.76	1.49
1650.00	27.5		0.12	0.07	3.81	1.47
1890.00	31.5		0.11	0.05	3.54	1.32
2130.00	35.5		0.11	0.07	3.67	1.67
2400.00	40		0.11	0.06	3.51	1.49
2700.00	45		0.10	0.06	3.38	1.49
3000.00	50		0.10	0.06	3.36	1.53
3300.00	55		0.10	0.06	3.29	1.44
3525.00	58.75		0.09	0.05	3.13	1.45
15600.00	240		0.03	0.02	1.19	0.56

**Blood**						
**Time (s)**	**Acquisition length (s)**	**Average Injected Dose (MBq)**	**Averaged Activity (MBq)**	**SD**	**%ID.cm^{-3}**	**SD**
15.00	0.25		0.43	0.29	13.43	6.08
45.00	0.75		0.44	0.17	14.56	3.25
75.00	1.25		0.38	0.14	13.10	3.91
120.00	2		0.30	0.13	10.00	2.76
180.00	3		0.25	0.11	8.33	2.33
240.00	4		0.21	0.09	7.01	1.51
300.00	5		0.19	0.08	6.11	1.44
360.00	6		0.17	0.09	5.55	1.62
450.00	7.5		0.15	0.07	5.02	1.28
570.00	9.5		0.13	0.06	4.28	1.06
690.00	11.5		0.11	0.05	3.70	0.87
810.00	13.5		0.10	0.06	3.41	1.04
930.00	15.5	**3**	0.09	0.04	3.02	0.71

1080.00	18		0.09	0.04	2.80	0.83
1260.00	21		0.08	0.04	2.57	0.66
1440.00	24		0.07	0.03	2.27	0.51
1650.00	27.5		0.07	0.03	2.13	0.59
1890.00	31.5		0.06	0.03	1.93	0.55
2130.00	35.5		0.06	0.03	1.82	0.47
2400.00	40		0.05	0.02	1.73	0.40
2700.00	45		0.05	0.02	1.59	0.37
3000.00	50		0.05	0.02	1.60	0.42
3300.00	55		0.04	0.02	1.46	0.45
3525.00	58.75		0.04	0.02	1.50	0.59
15600.00	240		0.01	0.00	0.27	0.12

**Muscle**						
**Time (s)**	**Acquisition length (s)**	**Average Injected Dose (MBq)**	**Averaged Activity (MBq)**	**SD**	**%ID.cm^{-3}**	**SD**
15.00	0.25		0.01	0.01	0.37	0.30
45.00	0.75		0.03	0.02	0.80	0.40
75.00	1.25		0.03	0.01	1.06	0.20
120.00	2		0.03	0.02	1.03	0.37
180.00	3		0.04	0.01	1.22	0.32
240.00	4		0.04	0.01	1.29	0.35
300.00	5		0.04	0.02	1.42	0.38
360.00	6		0.04	0.02	1.42	0.33
450.00	7.5		0.04	0.02	1.37	0.30
570.00	9.5		0.04	0.02	1.38	0.33
690.00	11.5		0.04	0.02	1.26	0.32
810.00	13.5		0.04	0.02	1.25	0.30
930.00	15.5	**3**	0.04	0.01	1.25	0.18
1080.00	18		0.03	0.01	1.15	0.22
1260.00	21		0.03	0.01	1.07	0.26
1440.00	24		0.03	0.01	0.95	0.24
1650.00	27.5		0.03	0.01	0.87	0.31
1890.00	31.5		0.03	0.01	0.85	0.14
2130.00	35.5		0.02	0.01	0.76	0.16
2400.00	40		0.02	0.01	0.71	0.16
2700.00	45		0.02	0.01	0.68	0.22
3000.00	50		0.02	0.01	0.61	0.18
3300.00	55		0.02	0.01	0.53	0.16
3525.00	58.75		0.01	0.01	0.47	0.15

15600.00	240		0.00	0.00	0.13	0.05

**Brain**						
**Time (s)**	**Acquisition length (s)**	**Average Injected Dose (MBq)**	**Averaged Activity (MBq)**	**SD**	**%ID.cm^{-3}**	**SD**
15.00	0.25		0.03	0.01	0.86	0.27
45.00	0.75		0.04	0.01	1.38	0.29
75.00	1.25		0.03	0.01	1.10	0.23
120.00	2		0.03	0.01	0.92	0.12
180.00	3		0.02	0.01	0.80	0.24
240.00	4		0.02	0.01	0.62	0.09
300.00	5		0.02	0.01	0.60	0.19
360.00	6		0.02	0.01	0.55	0.15
450.00	7.5		0.02	0.01	0.49	0.14
570.00	9.5		0.01	0.00	0.49	0.08
690.00	11.5		0.01	0.00	0.38	0.06
810.00	13.5		0.01	0.01	0.42	0.12
930.00	15.5	**3**	0.01	0.00	0.36	0.08
1080.00	18		0.01	0.00	0.34	0.09
1260.00	21		0.01	0.00	0.33	0.08
1440.00	24		0.01	0.01	0.32	0.11
1650.00	27.5		0.01	0.00	0.30	0.05
1890.00	31.5		0.01	0.00	0.34	0.09
2130.00	35.5		0.01	0.00	0.28	0.04
2400.00	40		0.01	0.00	0.27	0.05
2700.00	45		0.01	0.00	0.28	0.08
3000.00	50		0.01	0.00	0.29	0.04
3300.00	55		0.01	0.00	0.25	0.04
3525.00	58.75		0.01	0.00	0.24	0.05
15600.00	240		0.00	0.00	0.10	0.05

Supplemental Table 8. Averaged PET-obtained activities in CHO-304 xenografted NMRI-*Foxn1*$^{nu/nu}$ mice following i.v. injection of 20 nmol/kg [^{18}F]F-MQ232, by organ.

Tumor						
**Time (s)**	**Acquisition length (s)**	**Average Injected Dose (MBq)**	**Averaged Activity (MBq)**	**SD**	**%ID.cm^{-3}**	**SD**
15.00	0.25		0.02	0.02	0.33	0.28
45.00	0.75		0.06	0.04	0.98	0.20
75.00	1.25		0.07	0.05	1.24	0.25
120.00	2		0.08	0.06	1.41	0.35
180.00	3		0.09	0.07	1.58	0.45
240.00	4		0.10	0.08	1.79	0.51
300.00	5		0.11	0.08	1.90	0.56
360.00	6		0.12	0.09	1.99	0.59
450.00	7.5		0.12	0.09	2.11	0.61
570.00	9.5		0.13	0.10	2.20	0.67
690.00	11.5		0.14	0.10	2.35	0.65
810.00	13.5		0.14	0.10	2.43	0.69
930.00	15.5	**6.8**	0.14	0.10	2.49	0.72
1080.00	18		0.15	0.11	2.58	0.74
1260.00	21		0.15	0.11	2.67	0.77
1440.00	24		0.16	0.11	2.75	0.78
1650.00	27.5		0.16	0.11	2.80	0.82
1890.00	31.5		0.16	0.11	2.92	0.83
2130.00	35.5		0.17	0.12	2.95	0.85
2400.00	40		0.17	0.12	3.02	0.89
2700.00	45		0.17	0.12	3.07	0.89
3000.00	50		0.18	0.12	3.14	0.91
3300.00	55		0.18	0.12	3.18	0.92
3525.00	58.75		0.18	0.12	3.20	0.92
15600.00	240		0.13	0.08	2.58	0.90

Kidney						
**Time (s)**	**Acquisition length (s)**	**Average Injected Dose (MBq)**	**Averaged Activity (MBq)**	**SD**	**%ID.cm^{-3}**	**SD**
	0.25		0.33	0.35	4.62	3.99

45.00	0.75		0.61	0.46	10.18	3.47
75.00	1.25		0.69	0.56	11.38	4.59
120.00	2		0.79	0.67	12.82	5.90
180.00	3		0.90	0.79	14.41	7.37
240.00	4		1.02	0.92	16.21	8.84
300.00	5		1.11	1.01	17.60	9.93
360.00	6		1.19	1.09	18.75	10.81
450.00	7.5		1.28	1.20	20.12	12.01
570.00	9.5		1.39	1.31	21.59	13.29
690.00	11.5		1.50	1.41	23.51	14.24
810.00	13.5		1.57	1.46	24.64	14.77
930.00	15.5	**6.8**	1.61	1.49	25.22	15.09
1080.00	18		1.67	1.54	26.18	15.53
1260.00	21		1.72	1.57	26.97	15.80
1440.00	24		1.74	1.57	27.40	15.71
1650.00	27.5		1.73	1.53	27.32	15.28
1890.00	31.5		1.69	1.47	27.08	14.48
2130.00	35.5		1.65	1.41	26.53	13.79
2400.00	40		1.59	1.32	25.66	12.84
2700.00	45		1.48	1.20	24.26	11.62
3000.00	50		1.38	1.08	23.02	10.50
3300.00	55		1.29	0.96	21.88	9.53
3525.00	58.75		1.23	0.88	21.14	9.06
15600.00	240		0.28	0.18	4.44	4.13

**Liver**						
**Time (s)**	**Acquisition length (s)**	**Average Injected Dose (MBq)**	**Averaged Activity (MBq)**	**SD**	**%ID.cm^{-3}**	**SD**
15.00	0.25		0.22	0.25	3.00	3.01
45.00	0.75		0.48	0.27	8.92	0.63
75.00	1.25		0.52	0.21	10.38	1.71
120.00	2		0.51	0.17	10.49	2.42
180.00	3		0.50	0.14	10.53	3.06
240.00	4		0.49	0.13	10.49	3.33
300.00	5		0.48	0.12	10.33	3.47
360.00	6		0.47	0.11	10.45	3.80
450.00	7.5		0.45	0.11	10.04	3.75
570.00	9.5		0.43	0.11	9.56	3.55
690.00	11.5		0.43	0.10	9.81	4.10
810.00	13.5		0.43	0.09	9.67	4.13

930.00	15.5	**6.8**	0.42	0.10	9.40	3.99
1080.00	18		0.41	0.09	9.31	4.01
1260.00	21		0.40	0.10	9.06	3.96
1440.00	24		0.39	0.09	9.04	4.05
1650.00	27.5		0.39	0.09	8.86	3.96
1890.00	31.5		0.39	0.09	9.01	4.18
2130.00	35.5		0.38	0.09	8.65	3.93
2400.00	40		0.38	0.09	8.68	4.07
2700.00	45		0.37	0.09	8.48	3.96
3000.00	50		0.36	0.08	8.41	4.04
3300.00	55		0.35	0.08	8.28	4.03
3525.00	58.75		0.35	0.09	8.25	4.08
15600.00	240		0.25	0.07	6.41	4.21

Gallbladder

Time (s)	Acquisition length (s)	Average Injected Dose (MBq)	Averaged Activity (MBq)	SD	%ID.cc^{-3}	SD
15600.00	240	**6.8**	1.70	0.34	25.44	8.59

Bone Junction

Time (s)	Acquisition length (s)	Average Injected Dose (MBq)	Averaged Activity (MBq)	SD	%ID.cm^{-3}	SD
15.00	0.25		0.03	0.04	0.40	0.43
45.00	0.75		0.08	0.07	1.40	0.54
75.00	1.25		0.09	0.06	1.55	0.38
120.00	2		0.09	0.06	1.62	0.30
180.00	3		0.09	0.06	1.58	0.30
240.00	4		0.09	0.06	1.69	0.39
300.00	5		0.09	0.07	1.65	0.61
360.00	6		0.09	0.06	1.65	0.45
450.00	7.5		0.09	0.06	1.63	0.33
570.00	9.5		0.09	0.06	1.62	0.55
690.00	11.5		0.08	0.06	1.58	0.47
810.00	13.5		0.09	0.05	1.60	0.34
930.00	15.5	**6.8**	0.08	0.06	1.41	0.47
1080.00	18		0.08	0.06	1.49	0.59

1260.00	21		0.08	0.06	1.43	0.50
1440.00	24		0.08	0.06	1.50	0.56
1650.00	27.5		0.08	0.06	1.54	0.62
1890.00	31.5		0.08	0.06	1.48	0.59
2130.00	35.5		0.07	0.06	1.39	0.68
2400.00	40		0.08	0.06	1.48	0.66
2700.00	45		0.07	0.06	1.38	0.66
3000.00	50		0.07	0.07	1.37	0.78
3300.00	55		0.07	0.06	1.37	0.70
3525.00	58.75		0.07	0.06	1.31	0.75
15600.00	240		0.04	0.02	0.86	0.21

**Blood**						
**Time (s)**	**Acquisition length (s)**	**Average Injected Dose (MBq)**	**Averaged Activity (MBq)**	**SD**	**%ID.cm^{-3}**	**SD**
15.00	0.25		0.77	0.64	12.14	5.71
45.00	0.75		0.69	0.35	13.13	1.38
75.00	1.25		0.58	0.29	11.01	1.07
120.00	2		0.46	0.23	8.74	0.86
180.00	3		0.37	0.18	6.97	0.75
240.00	4		0.32	0.16	6.13	0.70
300.00	5		0.28	0.13	5.44	0.90
360.00	6		0.26	0.12	5.00	0.80
450.00	7.5		0.23	0.10	4.44	0.82
570.00	9.5		0.19	0.08	3.73	0.92
690.00	11.5		0.17	0.06	3.42	0.97
810.00	13.5		0.15	0.06	3.12	0.83
930.00	15.5	**6.8**	0.14	0.05	2.93	0.99
1080.00	18		0.13	0.05	2.71	0.90
1260.00	21		0.12	0.04	2.49	0.94
1440.00	24		0.11	0.04	2.30	0.91
1650.00	27.5		0.10	0.03	2.17	0.92
1890.00	31.5		0.10	0.03	2.11	0.96
2130.00	35.5		0.09	0.03	1.97	0.99
2400.00	40		0.09	0.03	1.92	1.02
2700.00	45		0.08	0.03	1.79	0.96
3000.00	50		0.08	0.03	1.77	1.01
3300.00	55		0.07	0.02	1.63	0.96
3525.00	58.75		0.07	0.03	1.64	1.02
15600.00	240		0.02	0.01	0.32	0.09

Muscle						
Time (s)	Acquisition length (s)	Average Injected Dose (MBq)	Averaged Activity (MBq)	SD	%ID.cm^{-3}	SD
15.00	0.25		0.01	0.01	0.16	0.17
45.00	0.75		0.04	0.04	0.61	0.42
75.00	1.25		0.03	0.03	0.52	0.24
120.00	2		0.04	0.02	0.61	0.14
180.00	3		0.04	0.03	0.66	0.28
240.00	4		0.04	0.03	0.67	0.26
300.00	5		0.05	0.04	0.84	0.31
360.00	6		0.04	0.03	0.80	0.17
450.00	7.5		0.05	0.03	0.89	0.18
570.00	9.5		0.05	0.02	0.85	0.16
690.00	11.5		0.04	0.03	0.86	0.20
810.00	13.5		0.04	0.03	0.77	0.24
930.00	15.5	**6.8**	0.04	0.02	0.81	0.37
1080.00	18		0.04	0.02	0.76	0.26
1260.00	21		0.04	0.02	0.83	0.28
1440.00	24		0.04	0.02	0.81	0.35
1650.00	27.5		0.03	0.02	0.58	0.28
1890.00	31.5		0.03	0.01	0.70	0.38
2130.00	35.5		0.03	0.01	0.59	0.24
2400.00	40		0.03	0.01	0.66	0.36
2700.00	45		0.03	0.01	0.61	0.40
3000.00	50		0.03	0.01	0.64	0.42
3300.00	55		0.03	0.01	0.63	0.51
3525.00	58.75		0.03	0.01	0.58	0.45
15600.00	240		0.01	0.00	0.14	0.07

Brain						
Time (s)	Acquisition length (s)	Average Injected Dose (MBq)	Averaged Activity (MBq)	SD	%ID.cm^{-3}	SD
15.00	0.25		0.05	0.04	0.75	0.36
45.00	0.75		0.04	0.02	0.88	0.15
75.00	1.25		0.04	0.02	0.76	0.10
120.00	2		0.04	0.02	0.71	0.10

180.00	3		0.03	0.02	0.59	0.14
240.00	4		0.03	0.01	0.50	0.07
300.00	5		0.02	0.01	0.45	0.12
360.00	6		0.02	0.01	0.45	0.12
450.00	7.5		0.02	0.01	0.41	0.10
570.00	9.5		0.02	0.01	0.35	0.04
690.00	11.5		0.02	0.01	0.36	0.10
810.00	13.5		0.02	0.01	0.32	0.10
930.00	15.5	**6.8**	0.02	0.01	0.31	0.09
1080.00	18		0.01	0.00	0.29	0.08
1260.00	21		0.01	0.01	0.29	0.06
1440.00	24		0.01	0.00	0.26	0.07
1650.00	27.5		0.01	0.01	0.29	0.09
1890.00	31.5		0.01	0.00	0.25	0.09
2130.00	35.5		0.01	0.00	0.25	0.07
2400.00	40		0.01	0.00	0.26	0.09
2700.00	45		0.01	0.00	0.24	0.07
3000.00	50		0.01	0.00	0.26	0.10
3300.00	55		0.01	0.00	0.25	0.10
3525.00	58.75		0.01	0.00	0.23	0.11
15600.00	240		0.01	0.00	0.15	0.05

Supplementary Table 9. Averaged PET-obtained activities in CHO-304 xenografted NMRI-*Foxn1*$^{nu/nu}$ mice following i.v. injection of 20 nmol/kg [^{18}F]F-MQ.IMPAIRED, by organ.

**Tumor**						
**Time (s)**	**Acquisition length (s)**	**Average Injected Dose (MBq)**	**Averaged Activity (MBq)**	**SD**	**%ID.cm^{-3}**	**SD**
15.00	0.25		0.01	0.01	0.23	0.14
45.00	0.75		0.04	0.02	0.76	0.29
75.00	1.25		0.05	0.02	0.92	0.35
120.00	2		0.06	0.02	1.02	0.47
180.00	3		0.06	0.02	1.02	0.47
240.00	4		0.06	0.03	1.17	0.48
300.00	5		0.07	0.03	1.19	0.52
360.00	6		0.07	0.02	1.20	0.51
450.00	7.5		0.07	0.03	1.20	0.50
570.00	9.5		0.07	0.02	1.22	0.53

690.00	11.5		0.06	0.02	1.15	0.48
810.00	13.5		0.06	0.02	1.16	0.46
930.00	15.5	**5.8**	0.06	0.02	1.14	0.44
1080.00	18		0.06	0.02	1.07	0.40
1260.00	21		0.06	0.02	1.08	0.39
1440.00	24		0.06	0.02	1.07	0.39
1650.00	27.5		0.06	0.01	1.02	0.35
1890.00	31.5		0.06	0.01	0.99	0.32
2130.00	35.5		0.05	0.01	0.96	0.31
2400.00	40		0.05	0.01	0.96	0.29
2700.00	45		0.05	0.01	0.92	0.27
3000.00	50		0.05	0.01	0.89	0.24
3300.00	55		0.05	0.01	0.88	0.25
3525.00	58.75		0.05	0.01	0.87	0.23
15600.00	240		0.02	0.00	0.37	0.08

**Kidney**						
**Time (s)**	**Acquisition length (s)**	**Average Injected Dose (MBq)**	**Averaged Activity (MBq)**	**SD**	**%ID.cm^{-3}**	**SD**
	0.25		0.15	0.05	2.79	0.87
45.00	0.75		0.42	0.15	7.59	2.82
75.00	1.25		0.49	0.20	8.89	3.97
120.00	2		0.57	0.26	10.42	5.26
180.00	3		0.61	0.29	11.26	6.31
240.00	4		0.76	0.41	13.67	7.63
300.00	5		0.82	0.45	14.78	8.59
360.00	6		0.86	0.49	15.52	9.25
450.00	7.5		0.92	0.53	16.56	10.11
570.00	9.5		0.98	0.58	17.72	11.09
690.00	11.5		1.04	0.62	18.83	11.97
810.00	13.5		1.10	0.66	19.86	12.63
930.00	15.5	**5.8**	1.15	0.70	20.86	13.47
1080.00	18		1.20	0.73	21.57	13.92
1260.00	21		1.27	0.76	22.92	14.72
1440.00	24		1.30	0.78	23.40	14.87
1650.00	27.5		1.32	0.79	23.69	14.82
1890.00	31.5		1.34	0.81	23.72	14.20
2130.00	35.5		1.33	0.81	23.53	13.69
2400.00	40		1.34	0.80	23.57	13.40

2700.00	45		1.31	0.79	22.79	12.45
3000.00	50		1.29	0.77	22.28	11.39
3300.00	55		1.27	0.75	21.82	10.73
3525.00	58.75		1.25	0.74	21.22	10.01
15600.00	240		0.19	0.16	3.54	3.87

**Liver**						
**Time (s)**	**Acquisition length (s)**	**Average Injected Dose (MBq)**	**Averaged Activity (MBq)**	**SD**	**%ID.cm^{-3}**	**SD**
15.00	0.25		0.12	0.06	2.27	1.16
45.00	0.75		0.44	0.09	7.98	2.40
75.00	1.25		0.48	0.12	8.82	2.94
120.00	2		0.49	0.13	9.08	3.25
180.00	3		0.47	0.16	8.72	3.86
240.00	4		0.52	0.19	9.59	4.09
300.00	5		0.51	0.20	9.42	4.29
360.00	6		0.51	0.21	9.31	4.53
450.00	7.5		0.49	0.22	9.08	4.72
570.00	9.5		0.48	0.24	8.81	4.96
690.00	11.5		0.47	0.25	8.78	5.16
810.00	13.5		0.46	0.26	8.55	5.30
930.00	15.5	**5.8**	0.46	0.27	8.53	5.50
1080.00	18		0.44	0.27	8.22	5.49
1260.00	21		0.46	0.29	8.48	5.89
1440.00	24		0.44	0.29	8.23	5.92
1650.00	27.5		0.44	0.29	8.09	5.97
1890.00	31.5		0.44	0.29	8.09	6.01
2130.00	35.5		0.43	0.30	8.02	6.10
2400.00	40		0.43	0.30	8.00	6.15
2700.00	45		0.42	0.30	7.82	6.10
3000.00	50		0.43	0.32	7.98	6.49
3300.00	55		0.42	0.32	7.83	6.40
3525.00	58.75		0.42	0.32	7.84	6.51
15600.00	240		0.25	0.26	4.57	5.11

**Gallbladder**						
	**Acquisition length (s)**	**Average Injected**	**Averaged Activity (MBq)**			

Time (s)		Dose (MBq)		SD	%ID.cm^{-3}	SD
15600.00	240	**5.8**	1.04	0.96	17.23	15.41

Bone Junction						
**Time (s)**	**Acquisition length (s)**	**Average Injected Dose (MBq)**	**Averaged Activity (MBq)**	**SD**	**%ID.cm^{-3}**	**SD**
15.00	0.25		0.02	0.01	0.39	0.22
45.00	0.75		0.07	0.03	1.18	0.47
75.00	1.25		0.07	0.03	1.20	0.47
120.00	2		0.07	0.02	1.28	0.46
180.00	3		0.07	0.03	1.18	0.47
240.00	4		0.08	0.03	1.35	0.44
300.00	5		0.07	0.03	1.31	0.47
360.00	6		0.07	0.03	1.28	0.48
450.00	7.5		0.07	0.03	1.27	0.43
570.00	9.5		0.07	0.02	1.35	0.48
690.00	11.5		0.07	0.02	1.31	0.36
810.00	13.5		0.08	0.02	1.44	0.38
930.00	15.5	**5.8**	0.07	0.03	1.30	0.37
1080.00	18		0.07	0.03	1.23	0.46
1260.00	21		0.07	0.02	1.24	0.37
1440.00	24		0.07	0.02	1.20	0.26
1650.00	27.5		0.07	0.02	1.24	0.27
1890.00	31.5		0.07	0.02	1.17	0.26
2130.00	35.5		0.06	0.02	1.07	0.30
2400.00	40		0.06	0.02	1.06	0.30
2700.00	45		0.06	0.02	1.09	0.28
3000.00	50		0.06	0.02	1.11	0.18
3300.00	55		0.06	0.02	1.02	0.23
3525.00	58.75		0.06	0.02	1.05	0.21
15600.00	240		0.04	0.00	0.72	0.23

Blood						
**Time (s)**	**Acquisition length (s)**	**Average Injected Dose (MBq)**	**Averaged Activity (MBq)**	**SD**	**%ID.cm^{-3}**	**SD**
15.00	0.25		0.46	0.18	7.95	2.22

45.00	0.75		0.61	0.20	10.82	2.72
75.00	1.25		0.48	0.15	8.61	2.28
120.00	2		0.40	0.10	7.04	1.49
180.00	3		0.30	0.06	5.45	1.22
240.00	4		0.31	0.09	5.33	0.82
300.00	5		0.27	0.08	4.78	0.90
360.00	6		0.25	0.07	4.43	0.79
450.00	7.5		0.22	0.05	3.88	0.68
570.00	9.5		0.19	0.05	3.31	0.66
690.00	11.5		0.17	0.05	3.02	0.73
810.00	13.5		0.15	0.05	2.67	0.79
930.00	15.5	**5.8**	0.15	0.04	2.62	0.66
1080.00	18		0.13	0.04	2.30	0.65
1260.00	21		0.13	0.03	2.24	0.58
1440.00	24		0.12	0.04	2.12	0.62
1650.00	27.5		0.11	0.04	1.95	0.59
1890.00	31.5		0.10	0.03	1.81	0.50
2130.00	35.5		0.10	0.03	1.66	0.50
2400.00	40		0.09	0.03	1.62	0.56
2700.00	45		0.09	0.03	1.57	0.58
3000.00	50		0.09	0.03	1.49	0.57
3300.00	55		0.08	0.03	1.44	0.50
3525.00	58.75		0.08	0.03	1.36	0.56
15600.00	240		0.02	0.01	0.31	0.15

**Muscle**						
**Time (s)**	**Acquisition length (s)**	**Average Injected Dose (MBq)**	**Averaged Activity (MBq)**	**SD**	**%ID.cm^{-3}**	**SD**
15.00	0.25		0.01	0.01	0.10	0.10
45.00	0.75		0.03	0.02	0.48	0.20
75.00	1.25		0.03	0.01	0.48	0.19
120.00	2		0.04	0.02	0.67	0.21
180.00	3		0.04	0.02	0.64	0.35
240.00	4		0.04	0.02	0.74	0.23
300.00	5		0.05	0.02	0.84	0.29
360.00	6		0.04	0.02	0.76	0.34
450.00	7.5		0.04	0.02	0.78	0.29
570.00	9.5		0.04	0.02	0.76	0.33
690.00	11.5		0.04	0.02	0.74	0.27
810.00	13.5		0.04	0.01	0.74	0.26

930.00	15.5	**5.8**	0.04	0.01	0.77	0.21
1080.00	18		0.04	0.02	0.73	0.29
1260.00	21		0.04	0.01	0.62	0.17
1440.00	24		0.04	0.01	0.66	0.20
1650.00	27.5		0.04	0.01	0.63	0.22
1890.00	31.5		0.03	0.01	0.56	0.17
2130.00	35.5		0.03	0.01	0.53	0.16
2400.00	40		0.03	0.01	0.52	0.10
2700.00	45		0.03	0.01	0.52	0.08
3000.00	50		0.03	0.01	0.50	0.11
3300.00	55		0.02	0.01	0.43	0.11
3525.00	58.75		0.03	0.01	0.44	0.09
15600.00	240		0.01	0.00	0.12	0.04

**Brain**						
**Time (s)**	**Acquisition length (s)**	**Average Injected Dose (MBq)**	**Averaged Activity (MBq)**	**SD**	**%ID.cm^{-3}**	**SD**
15.00	0.25		0.03	0.01	0.57	0.21
45.00	0.75		0.04	0.01	0.76	0.23
75.00	1.25		0.04	0.01	0.66	0.20
120.00	2		0.03	0.01	0.59	0.16
180.00	3		0.03	0.00	0.49	0.17
240.00	4		0.03	0.00	0.49	0.15
300.00	5		0.03	0.01	0.47	0.14
360.00	6		0.03	0.00	0.46	0.10
450.00	7.5		0.02	0.00	0.38	0.12
570.00	9.5		0.02	0.00	0.38	0.09
690.00	11.5		0.02	0.00	0.37	0.09
810.00	13.5		0.02	0.00	0.33	0.07
930.00	15.5	**5.8**	0.02	0.00	0.30	0.08
1080.00	18		0.02	0.00	0.31	0.09
1260.00	21		0.02	0.00	0.29	0.09
1440.00	24		0.01	0.00	0.27	0.07
1650.00	27.5		0.01	0.00	0.27	0.08
1890.00	31.5		0.01	0.00	0.26	0.07
2130.00	35.5		0.01	0.00	0.26	0.07
2400.00	40		0.01	0.00	0.26	0.07
2700.00	45		0.01	0.00	0.25	0.07
3000.00	50		0.01	0.00	0.25	0.08
3300.00	55		0.01	0.00	0.24	0.07

3525.00	58.75		0.01	0.00	0.25	0.07
15600.00	240		0.01	0.00	0.09	0.07

Supplementary Table 10. Corrected time-activity curve data for each NMRI-*Foxn1*$^{nu/nu}$ mouse used for plasma half-life determination of [^{18}F]F-MQ232.

The one compartment model was initiated with this data set to provide it with a starting point. Modelling results for each subject are provided as screenshots of the report given by PKIN (PMOD pharmacokinetics analyzing software)

Time (min)	Mouse 1 Time Corrected Blood Activity (kBq)	Mouse 2 Time Corrected Blood Activity (kBq)	Mouse 3 Time Corrected Blood Activity (kBq)	Mouse 4 Time Corrected Blood Activity (kBq)	Mouse 5 Time Corrected Blood Activity (kBq)	Mouse 6 Time Corrected Blood Activity (kBq)
0.75	754.01	476.86	314.63	284.08	346.53	459.94
1.25	644.78	394.05	264.71	282.48	386.18	337.39
2	561.58	302.88	208.58	197.34	252.10	287.54
3	462.99	243.01	169.05	173.76	212.57	238.45
4	383.05	214.31	167.45	158.49	149.85	199.14
5	347.95	176.52	145.47	132.63	126.09	188.57
6	341.57	155.74	132.21	111.29	123.83	153.88
7.5	291.85	154.52	121.61	99.18	115.00	136.17
9.5	241.36	110.01	111.69	88.73	101.79	122.01
11.5	214.25	97.21	108.14	69.95	77.68	113.91
13.5	214.73	89.72	87.58	62.33	74.84	100.53
15.5	174.36	84.32	85.56	54.82	65.57	90.95
18	172.99	60.52	80.98	57.05	57.69	86.91
21	149.60	61.24	75.17	48.00	56.68	80.51
24	119.32	48.17	73.03	48.57	52.72	69.00
27.5	129.18	47.42	66.31	44.19	41.34	65.91
31.5	116.67	41.88	60.79	36.62	38.80	62.78
35.5	109.15	45.76	57.70	34.62	34.69	56.58
40	98.04	40.82	55.92	34.96	34.62	54.44
45	87.64	34.67	50.65	33.27	34.66	49.62
50	91.35	34.92	46.82	31.94	36.73	49.47
55	85.21	30.47	39.44	28.66	38.38	42.51
58.75	86.04	27.22	39.16	25.86	46.75	43.61
240	10.4	6.7	5.9	5.0	9.0	7.3
**$t_{1/2}$ (min)**	7.72	4.38	4.38	5.72	4.24	6.24
**Moy (min)**	5.45					
**SD (min)**	1.39					

Mouse 1

Parameter	Current value	% SE	Conf.low	Conf.high	Unit
Delay	10.0	---	---	---	sec
Begin	10.0	---	---	---	sec
Amplitude 1	627.836504	5.91	551.05463	704.61837	kBq/cc
Halftime 1	7.720813	12.29	5.75822	9.68341	min
Amplitude 2	0.0	---	---	---	kBq/cc
Halftime 2	0.0	---	---	---	min
Amplitude 3	0.0	---	---	---	kBq/cc
Halftime 3	0.0	---	---	---	min

Parameter	Current value
DOF	23.0
SumSquared	199468.814448
ChiSquare	3469022.859963
AIC	378.945502
SC	380.837799
MSC	1.737903
R2	0.850117
Sy.x	93.126565
Runs test p	1.345165E-5
AUC	421868.541035
Iterations	10.0

Mouse 2

Parameter	Current value	% SE	Conf.low	Conf.high	Unit
Delay	0.0	---	---	---	sec
Begin	30.0	---	---	---	sec
Amplitude 1	401.290461	5.6	354.81999	447.76093	kBq/cc
Halftime 1	4.381767	11.57	3.33306	5.43047	min
Amplitude 2	0.0	---	---	---	kBq/cc
Halftime 2	0.0	---	---	---	min
Amplitude 3	0.0	---	---	---	kBq/cc
Halftime 3	0.0	---	---	---	min

Parameter	Current value
DOF	23.0
SumSquared	30253.000815
ChiSquare	526139.144602
AIC	331.793939
SC	333.686236
MSC	2.758585
R2	0.94599
Sy.x	36.267725
Runs test p	0.011525
AUC	-2223.511867
Iterations	8.0

Mouse 3

Parameter	Current value	% SE	Conf.low	Conf.high	Unit
Delay	10.0	---	---	---	sec
Begin	10.0	---	---	---	sec
Amplitude 1	423.008527	6.13	369.22008	476.79697	kBq/cc
Halftime 1	4.382049	11.83	3.3071	5.45699	min
Amplitude 2	0.0	---	---	---	kBq/cc
Halftime 2	0.0	---	---	---	min
Amplitude 3	0.0	---	---	---	kBq/cc
Halftime 3	0.0	---	---	---	min

Parameter	Current value
DOF	22.0
SumSquared	62476.590586
ChiSquare	1135938.01066
AIC	337.114419
SC	338.899098
MSC	1.988168
R2	0.884078
Sy.x	53.290196
Runs test p	0.00188
AUC	161989.399989
Iterations	8.0

Mouse 4

Parameter	Current value	% SE	Conf.low	Conf.high	Unit
Delay	10.0	---	---	---	sec
Begin	10.0	---	---	---	sec
Amplitude 1	261.7695	6.22	228.0755	295.4635	kBq/cc
Halftime 1	5.718909	12.44	4.24713	7.19069	min
Amplitude 2	0.0	---	---	---	kBq/cc
Halftime 2	0.0	---	---	---	min
Amplitude 3	0.0	---	---	---	kBq/cc
Halftime 3	0.0	---	---	---	min

Parameter	Current value
DOF	23.0
SumSquared	22023.373235
ChiSquare	383015.186694
AIC	323.856662
SC	325.748959
MSC	1.745480
R2	0.851249
Sy.x	30.94411
Runs test p	1.551985E-4
AUC	130055.007911
Iterations	9.0

Mouse 5

Parameter	Current value	% SE	Conf.low	Conf.high	Unit
Delay	10.0	---	---	---	sec
Begin	10.0	---	---	---	sec
Amplitude 1	350.693941	7.48	296.4359	404.95198	kBq/cc
Halftime 1	4.237051	14.36	2.97826	5.49584	min
Amplitude 2	0.0	---	---	---	kBq/cc
Halftime 2	0.0	---	---	---	min
Amplitude 3	0.0	---	---	---	kBq/cc
Halftime 3	0.0	---	---	---	min

Parameter	Current value
DOF	23.0
SumSquared	43036.991681
ChiSquare	748469.420547
AIC	340.605555
SC	342.497852
MSC	1.589884
R2	0.826206
Sy.x	43.257064
Runs test p	1.227578E-4
AUC	129247.787149
Iterations	7.0

Mouse 6

Parameter	Current value	% SE	Conf.low	Conf.high	Unit
Delay	10.0	---	---	---	sec
Begin	10.0	---	---	---	sec
Amplitude 1	358.083147	7.4	303.25225	412.91405	kBq/cc
Halftime 1	6.239482	14.97	4.30738	8.17159	min
Amplitude 2	0.0	---	---	---	kBq/cc
Halftime 2	0.0	---	---	---	min
Amplitude 3	0.0	---	---	---	kBq/cc
Halftime 3	0.0	---	---	---	min

Parameter	Current value
DOF	23.0
SumSquared	44099.006018
ChiSquare	766939.235087
AIC	341.214986
SC	343.107283
MSC	1.850519
R2	0.866081
Sy.x	43.787533
Runs test p	1.345165E-5
AUC	194326.52185
Iterations	10.0

Supplementary Table 11. Corrected time-activity curve data for each NMRI-*Foxn1*$^{nu/nu}$ mouse used for plasma half-life determination of [^{18}F]F-MQ.IMPAIRED.

The one compartment model was initiated with this data set to provide it with a starting point. Modelling results for each subject are provided as screenshots of the report given by PKIN (PMOD pharmacokinetics analyzing software)

Time (min)	Mouse 1 Time Corrected Blood Activity (kBq)	Mouse 2 Time Corrected Blood Activity (kBq)	Mouse 3 Time Corrected Blood Activity (kBq)	Mouse 4 Time Corrected Blood Activity (kBq)	Mouse 5 Time Corrected Blood Activity (kBq)	Mouse 6 Time Corrected Blood Activity (kBq)
0.75	587.403024	428.517489	539.271604	1005.49584	599.34058	523.513236
1.25	468.49267	335.212058	416.011912	762.376499	479.82122	445.460833
2	389.759752	316.634872	361.976303	593.192391	375.333986	343.506595
3	336.050155	243.594089	276.591111	415.896667	270.494128	283.209461
4	314.518695	257.861046	303.212787	472.752384	235.490632	248.962953
5	299.115274	216.020002	260.399069	411.648229	226.873639	218.472414
6	286.860258	214.628719	249.382282	369.619802	189.154679	207.035644
7.5	248.362044	190.31026	219.07339	307.697232	170.198818	184.094399
9.5	228.639756	147.400828	198.970972	257.195659	140.087105	157.141906
11.5	225.493021	144.570467	172.852518	234.933135	116.747522	136.875871
13.5	216.134877	110.989564	163.484227	203.009517	97.130614	125.282667
15.5	203.261796	125.4676	163.48756	188.930549	101.834304	112.892367
18	185.641429	103.41172	142.350445	166.512999	84.788111	102.385925
21	173.639154	112.082702	136.223245	158.097932	86.200952	97.98415
24	174.768442	103.434962	138.832364	146.205433	78.521044	84.04077
27.5	160.832642	101.609872	122.146467	139.815225	66.067413	77.852572
31.5	144.739032	94.504538	123.720263	127.131857	64.473112	69.955435
35.5	136.03241	89.524831	113.797356	110.507023	57.590291	63.578785
40	143.23993	72.801269	111.970324	107.664245	59.272892	62.508894
45	141.337509	79.242761	107.066619	97.273132	55.273785	56.902499
50	132.968922	84.66647	104.231587	93.127544	43.631136	52.742028
55	124.418209	78.056423	106.23112	86.357945	50.059907	49.132503
58.75	126.839753	72.647146	95.058193	90.779523	37.797807	48.866203
240	27.6436334	23.4899521	17.4954468	16.9443694	7.71737772	10.8807883
$t_{1/2}$ (min)	19.47	12.79	15.12	5.89	4.18	6.66
Moy (min)	10.69					
SD (min)	6.05					

Mouse 1	Mouse 2

Parameter	Current value	% SE	Conf.low	Conf.high	Unit
Delay	0.0	---	---	---	sec
Begin	30.0	---	---	---	sec
Amplitude 1	392.132773	6.45	339.67089	444.59465	kBq/cc
Halftime 1	19.4767	15.99	13.01553	25.93787	min
Amplitude 2	0.0	---	---	---	kBq/cc
Halftime 2	0.0	---	---	---	min
Amplitude 3	0.0	---	---	---	kBq/cc
Halftime 3	0.0	---	---	---	min
Parameter	Current value				
DOF	22.0				
SumSquared	84274.757391				
ChiSquare	1532268.316206				
AIC	344.29739				
SC	346.082069				
MSC	1.419869				
R2	0.795367				
Sy.x	61.892413				
Runs test p	4.84354E-5				
AUC	585200.819336				
Iterations	7.0				

Parameter	Current value	% SE	Conf.low	Conf.high	Unit
Delay	0.0	---	---	---	sec
Begin	30.0	---	---	---	sec
Amplitude 1	311.726707	6.37	270.62492	352.82849	kBq/cc
Halftime 1	12.786817	14.56	8.93429	16.63934	min
Amplitude 2	0.0	---	---	---	kBq/cc
Halftime 2	0.0	---	---	---	min
Amplitude 3	0.0	---	---	---	kBq/cc
Halftime 3	0.0	---	---	---	min
Parameter	Current value				
DOF	23.0				
SumSquared	44042.938036				
ChiSquare	765964.139754				
AIC	341.18318				
SC	343.075477				
MSC	1.629363				
R2	0.832933				
Sy.x	43.759689				
Runs test p	4.424189E-5				
AUC	353568.682479				
Iterations	12.0				

Mousse 3

Parameter	Current value	% SE	Conf.low	Conf.high	Unit
Delay	10.0	---	---	---	sec
Begin	10.0	---	---	---	sec
Amplitude 1	369.278191	6.98	315.91123	422.64515	kBq/cc
Halftime 1	15.120248	16.02	10.10888	20.13162	min
Amplitude 2	0.0	---	---	---	kBq/cc
Halftime 2	0.0	---	---	---	min
Amplitude 3	0.0	---	---	---	kBq/cc
Halftime 3	0.0	---	---	---	min
Parameter	Current value				
DOF	23.0				
SumSquared	76296.523201				
ChiSquare	1326896.055675				
AIC	354.919739				
SC	356.812036				
MSC	1.413727				
R2	0.792729				
Sy.x	57.595487				
Runs test p	1.325914E-5				
AUC	484307.004684				
Iterations	11.0				

Mouse 4

Parameter	Current value	% SE	Conf.low	Conf.high	Unit
Delay	10.0	---	---	---	sec
Begin	10.0	---	---	---	sec
Amplitude 1	785.053188	7.52	662.88778	907.2186	kBq/cc
Halftime 1	5.891067	15.1	4.05102	7.73112	min
Amplitude 2	0.0	---	---	---	kBq/cc
Halftime 2	0.0	---	---	---	min
Amplitude 3	0.0	---	---	---	kBq/cc
Halftime 3	0.0	---	---	---	min
Parameter	Current value				
DOF	23.0				
SumSquared	207878.011746				
ChiSquare	3615269.769502				
AIC	379.97784				
SC	381.870137				
MSC	1.856472				
R2	0.866876				
Sy.x	95.069314				
Runs test p	1.345165E-5				
AUC	402236.233962				
Iterations	10.0				

Mouse 5

Parameter	Current value	% SE	Conf.low	Conf.high	Unit
Delay	10.0	---	---	---	sec
Begin	10.0	---	---	---	sec
Amplitude 1	521.413179	7.18	443.9128	598.91356	kBq/cc
Halftime 1	4.184444	13.77	2.9922	5.37668	min
Amplitude 2	0.0	---	---	---	kBq/cc
Halftime 2	0.0	---	---	---	min
Amplitude 3	0.0	---	---	---	kBq/cc
Halftime 3	0.0	---	---	---	min
Parameter	Current value				
DOF	23.0				
SumSquared	78735.357671				
ChiSquare	1369310.568185				
AIC	355.706363				
SC	357.59866				
MSC	1.802687				
R2	0.85952				
Sy.x	58.508772				
Runs test p	1.227578E-4				
AUC	189884.947684				
Iterations	7.0				

Mouse 6

Parameter	Current value	% SE	Conf.low	Conf.high	Unit
Delay	10.0	---	---	---	sec
Begin	10.0	---	---	---	sec
Amplitude 1	427.777255	6.57	369.59371	485.9608	kBq/cc
Halftime 1	6.664448	13.41	4.81563	8.51327	min
Amplitude 2	0.0	---	---	---	kBq/cc
Halftime 2	0.0	---	---	---	min
Amplitude 3	0.0	---	---	---	kBq/cc
Halftime 3	0.0	---	---	---	min
Parameter	Current value				
DOF	23.0				
SumSquared	68541.105391				
ChiSquare	1192019.224199				
AIC	352.239896				
SC	354.132193				
MSC	1.643992				
R2	0.83536				
Sy.x	54.589816				
Runs test p	1.551985E-4				
AUC	247559.753806				
Iterations	10.0				

Supplementary Table 12. Averaged PET-obtained activities in Caki-1 xenografted NMRI-*Foxn1*$^{nu/nu}$ mice following i.v. injection of 20 nmol/kg [^{18}F]F-MQ232, by organ.

Tumor						
**Time (s)**	**Acquisition length (s)**	**Average Injected Dose (MBq)**	**Averaged Activity (MBq)**	**SD**	**%ID.cm^{-3}**	**SD**
15.00	0.25	**3.8**	0.00	0.00	0.09	0.05
45.00	0.75		0.02	0.01	0.54	0.16
75.00	1.25		0.03	0.02	0.77	0.25
120.00	2		0.03	0.02	0.83	0.22
180.00	3		0.04	0.02	1.02	0.30
240.00	4		0.05	0.03	1.23	0.43
300.00	5		0.04	0.02	1.15	0.31
360.00	6		0.05	0.03	1.36	0.48
450.00	7.5		0.05	0.03	1.38	0.49
570.00	9.5		0.05	0.03	1.41	0.50
690.00	11.5		0.05	0.03	1.38	0.48
810.00	13.5		0.05	0.03	1.40	0.49
930.00	15.5		0.05	0.03	1.41	0.52
1080.00	18		0.05	0.03	1.41	0.52
1260.00	21		0.05	0.03	1.40	0.52
1440.00	24		0.05	0.03	1.38	0.52
1650.00	27.5		0.05	0.03	1.37	0.54
1890.00	31.5		0.05	0.03	1.33	0.52
2130.00	35.5		0.05	0.03	1.32	0.52
2400.00	40		0.05	0.03	1.27	0.52
2700.00	45		0.05	0.03	1.24	0.51
3000.00	50		0.05	0.03	1.21	0.50
3300.00	55		0.05	0.03	1.21	0.53
3525.00	58.75		0.05	0.03	1.20	0.51
15600.00	240		0.02	0.01	0.38	0.16

Kidney						
**Time (s)**	**Acquisition length (s)**	**Average Injected Dose (MBq)**	**Averaged Activity (MBq)**	**SD**	**%ID.cm^{-3}**	**SD**

	0.25		0.08	0.03	2.32	1.05
45.00	0.75		0.35	0.11	9.84	0.93
75.00	1.25		0.43	0.15	11.97	1.13
120.00	2		0.48	0.14	13.38	0.96
180.00	3		0.59	0.21	16.35	1.34
240.00	4		0.70	0.27	19.14	2.16
300.00	5		0.68	0.21	19.09	0.98
360.00	6		0.83	0.34	22.68	3.20
450.00	7.5		0.89	0.36	24.22	3.12
570.00	9.5		0.95	0.38	26.14	3.17
690.00	11.5		1.01	0.39	27.74	3.12
810.00	13.5		1.07	0.42	29.32	3.40
930.00	15.5	**3.8**	1.12	0.43	30.64	3.47
1080.00	18		1.18	0.47	32.49	3.83
1260.00	21		1.26	0.50	34.49	4.31
1440.00	24		1.30	0.52	35.68	4.59
1650.00	27.5		1.36	0.57	37.16	5.36
1890.00	31.5		1.42	0.61	38.57	6.25
2130.00	35.5		1.46	0.67	39.50	7.43
2400.00	40		1.51	0.73	40.44	8.93
2700.00	45		1.55	0.81	41.34	10.93
3000.00	50		1.56	0.85	41.26	12.30
3300.00	55		1.62	0.97	42.41	15.16
3525.00	58.75		1.61	0.98	41.96	15.73
	240		0.16	0.10	4.78	4.04

**Liver**						
**Time (s)**	**Acquisition length (s)**	**Average Injected Dose (MBq)**	**Averaged Activity (MBq)**	**SD**	**%ID.cm^{-3}**	**SD**
15.00	0.25		0.06	0.02	1.71	0.93
45.00	0.75		0.36	0.14	9.81	1.97
75.00	1.25		0.39	0.16	10.71	2.39
120.00	2		0.37	0.14	10.34	2.13
180.00	3		0.40	0.17	11.02	2.46
240.00	4		0.42	0.18	11.54	2.29
300.00	5		0.37	0.14	10.36	2.05
360.00	6		0.43	0.19	11.65	2.41
450.00	7.5		0.41	0.17	11.21	2.22
570.00	9.5		0.40	0.16	10.86	1.90
690.00	11.5		0.39	0.15	10.56	1.91

810.00	13.5		0.38	0.15	10.44	1.75
930.00	15.5	**3.8**	0.37	0.14	10.15	1.81
1080.00	18		0.36	0.14	10.01	1.94
1260.00	21		0.36	0.14	9.98	1.88
1440.00	24		0.35	0.14	9.78	1.94
1650.00	27.5		0.35	0.13	9.72	1.80
1890.00	31.5		0.35	0.13	9.56	1.83
2130.00	35.5		0.34	0.13	9.44	1.80
2400.00	40		0.34	0.12	9.33	1.82
2700.00	45		0.33	0.12	9.26	1.76
3000.00	50		0.32	0.11	9.04	1.82
3300.00	55		0.33	0.12	9.11	1.77
3525.00	58.75		0.33	0.12	9.02	1.78
15600.00	240		0.17	0.05	4.80	2.27

**Gallbladder**						
**Time (s)**	**Acquisition length (s)**	**Average Injected Dose (MBq)**	**Averaged Activity (MBq)**	**SD**	**%ID.cc^{-3}**	**SD**
15600.00	240	**3.8**	0.48	0.21	12.77	3.84

**Bone Junction**						
**Time (s)**	**Acquisition length (s)**	**Average Injected Dose (MBq)**	**Averaged Activity (MBq)**	**SD**	**%ID.cm^{-3}**	**SD**
15.00	0.25		0.01	0.01	0.44	0.37
45.00	0.75		0.06	0.03	1.60	0.35
75.00	1.25		0.07	0.03	1.79	0.32
120.00	2		0.06	0.02	1.70	0.36
180.00	3		0.07	0.03	1.80	0.51
240.00	4		0.07	0.04	1.94	0.52
300.00	5		0.06	0.03	1.78	0.28
360.00	6		0.07	0.04	1.86	0.48
450.00	7.5		0.07	0.04	1.79	0.49
570.00	9.5		0.06	0.04	1.59	0.60
690.00	11.5		0.06	0.03	1.75	0.50
810.00	13.5		0.07	0.03	1.81	0.45

930.00	15.5	**3.8**	0.06	0.03	1.73	0.55
1080.00	18		0.06	0.03	1.62	0.40
1260.00	21		0.06	0.03	1.67	0.51
1440.00	24		0.06	0.03	1.68	0.53
1650.00	27.5		0.06	0.03	1.60	0.55
1890.00	31.5		0.06	0.03	1.65	0.47
2130.00	35.5		0.06	0.03	1.58	0.54
2400.00	40		0.06	0.03	1.64	0.57
2700.00	45		0.06	0.03	1.66	0.55
3000.00	50		0.06	0.03	1.57	0.46
3300.00	55		0.06	0.03	1.55	0.53
3525.00	58.75		0.06	0.03	1.53	0.45
15600.00	240		0.02	0.00	0.29	0.45

**Blood**						
**Time (s)**	**Acquisition length (s)**	**Average Injected Dose (MBq)**	**Averaged Activity (MBq)**	**SD**	**%ID.cm^{-3}**	**SD**
15.00	0.25		0.38	0.14	10.46	1.25
45.00	0.75		0.62	0.27	16.98	2.50
75.00	1.25		0.49	0.20	13.21	1.87
120.00	2		0.37	0.13	10.31	0.81
180.00	3		0.33	0.14	9.02	1.43
240.00	4		0.31	0.15	8.36	1.80
300.00	5		0.25	0.10	6.76	0.95
360.00	6		0.25	0.14	6.54	1.93
450.00	7.5		0.21	0.12	5.69	1.63
570.00	9.5		0.18	0.10	4.82	1.34
690.00	11.5		0.16	0.09	4.32	1.38
810.00	13.5		0.14	0.09	3.75	1.46
930.00	15.5	**3.8**	0.13	0.08	3.46	1.27
1080.00	18		0.13	0.08	3.26	1.17
1260.00	21		0.12	0.07	3.00	1.18
1440.00	24		0.11	0.07	2.80	1.08
1650.00	27.5		0.10	0.07	2.67	1.08
1890.00	31.5		0.09	0.06	2.44	0.99
2130.00	35.5		0.09	0.06	2.23	0.91
2400.00	40		0.08	0.05	2.15	0.90
2700.00	45		0.08	0.06	2.02	1.01
3000.00	50		0.07	0.05	1.82	0.81
3300.00	55		0.07	0.05	1.86	0.85

3525.00	58.75		0.07	0.05	1.81	0.81
15600.00	240		0.01	0.01	0.31	0.10

Muscle						
**Time (s)**	**Acquisition length (s)**	**Average Injected Dose (MBq)**	**Averaged Activity (MBq)**	**SD**	**%ID.cm^{-3}**	**SD**
15.00	0.25		0.00	0.00	0.11	0.09
45.00	0.75		0.02	0.01	0.42	0.16
75.00	1.25		0.02	0.01	0.55	0.27
120.00	2		0.02	0.01	0.64	0.21
180.00	3		0.03	0.01	0.69	0.11
240.00	4		0.03	0.01	0.75	0.20
300.00	5		0.03	0.01	0.69	0.10
360.00	6		0.03	0.01	0.83	0.10
450.00	7.5		0.03	0.01	0.81	0.14
570.00	9.5		0.03	0.02	0.86	0.26
690.00	11.5		0.03	0.01	0.82	0.18
810.00	13.5		0.03	0.02	0.84	0.29
930.00	15.5	**3.8**	0.03	0.01	0.74	0.15
1080.00	18		0.03	0.01	0.76	0.19
1260.00	21		0.03	0.01	0.68	0.16
1440.00	24		0.03	0.01	0.71	0.21
1650.00	27.5		0.02	0.01	0.66	0.17
1890.00	31.5		0.02	0.01	0.62	0.15
2130.00	35.5		0.02	0.01	0.60	0.17
2400.00	40		0.03	0.02	0.66	0.27
2700.00	45		0.02	0.01	0.57	0.22
3000.00	50		0.02	0.01	0.57	0.20
3300.00	55		0.02	0.01	0.55	0.19
3525.00	58.75		0.02	0.01	0.60	0.24
15600.00	240		0.00	0.00	0.12	0.05

Brain						
**Time (s)**	**Acquisition length (s)**	**Average Injected Dose (MBq)**	**Averaged Activity (MBq)**	**SD**	**%ID.cm^{-3}**	**SD**
15.00	0.25		0.02	0.01	0.58	0.04
45.00	0.75		0.04	0.02	1.11	0.33

75.00	1.25		0.04	0.02	0.99	0.23
120.00	2		0.03	0.01	0.77	0.15
180.00	3		0.02	0.01	0.66	0.11
240.00	4		0.03	0.01	0.69	0.14
300.00	5		0.02	0.01	0.57	0.07
360.00	6		0.02	0.01	0.56	0.20
450.00	7.5		0.02	0.01	0.53	0.16
570.00	9.5		0.02	0.01	0.48	0.08
690.00	11.5		0.02	0.01	0.43	0.22
810.00	13.5		0.01	0.01	0.34	0.08
930.00	15.5	**3.8**	0.01	0.01	0.36	0.12
1080.00	18		0.01	0.01	0.38	0.11
1260.00	21		0.01	0.01	0.35	0.14
1440.00	24		0.01	0.01	0.28	0.10
1650.00	27.5		0.01	0.01	0.31	0.09
1890.00	31.5		0.01	0.01	0.31	0.09
2130.00	35.5		0.01	0.01	0.31	0.10
2400.00	40		0.01	0.01	0.33	0.08
2700.00	45		0.01	0.01	0.30	0.08
3000.00	50		0.01	0.01	0.29	0.11
3300.00	55		0.01	0.01	0.30	0.09
3525.00	58.75		0.01	0.01	0.30	0.08
15600.00	240		0.01	0.00	0.18	0.05

Supplementary Table 13. qPCR data obtained on human ccRCC biopsies regarding hV2R expression

Sample type	Sample name	Sample’s origin	Target's species	Mean Ct (V2R)	Mean Ct (Housekeeping gene)
Human ccRCC biopsy	P1	Grade ≤ 2	Human V2R	27.00	8.73
	P2	Grade ≤ 2		27.19	9.45
	P3	Grade ≤ 2		31.00	7.57
	P4	Grade 3 or 4		30.34	8.95
	P5	Grade 3 or 4		30.43	9.01
	P6	Metastases		28.73	7.80
	P7	Metastases		27.20	9.81
	P8	Metastases		27.88	7.97

4 Supplementary Flowcharts

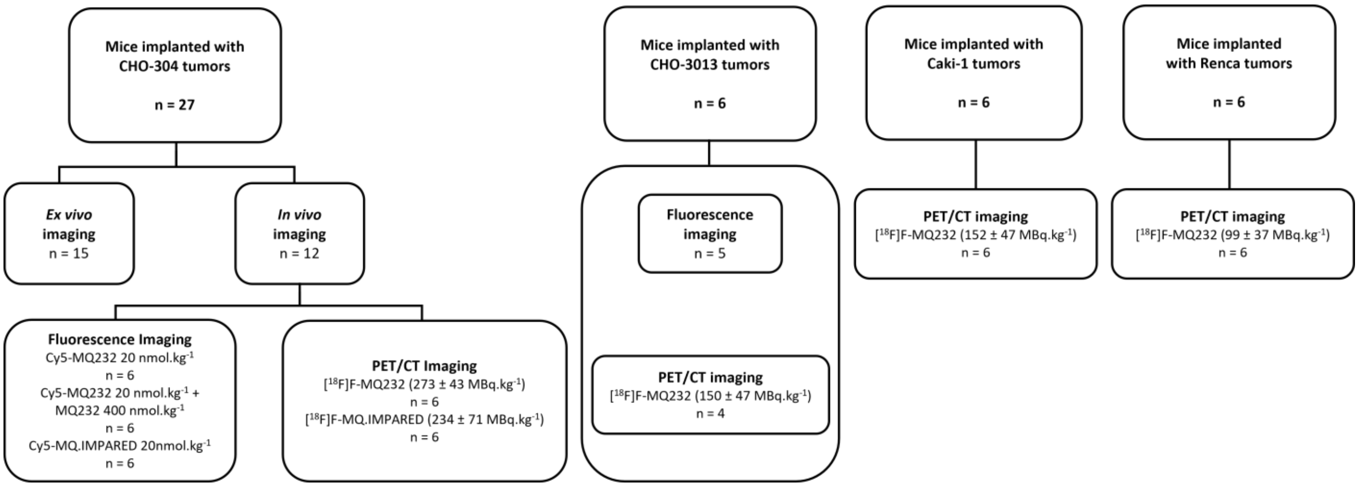

Supplementary Flowchart 1: Study flowchart including the number of mice used for this study

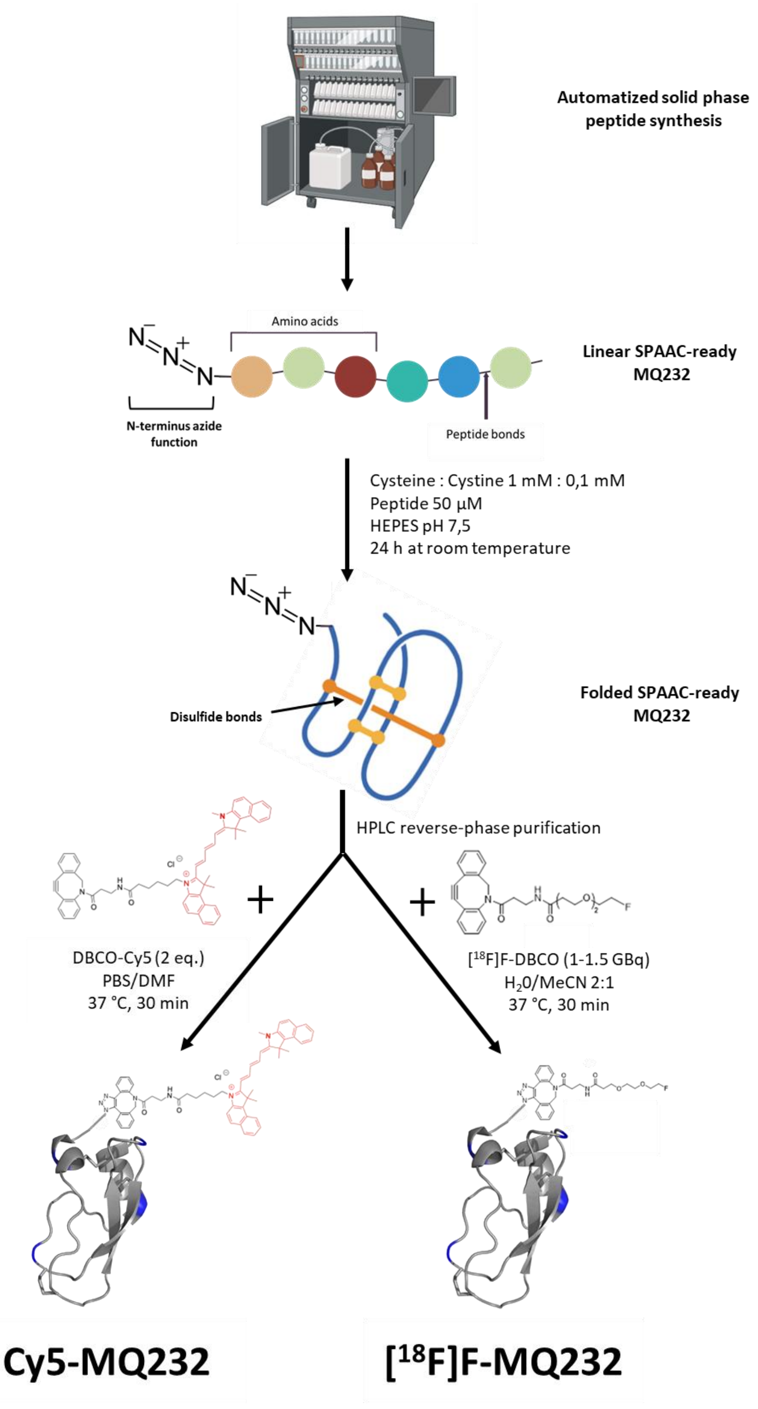

Supplementary Flowchart 2: Chemical steps toward MQ232-based molecular probes synthesis (the synthesis of the MQ-IMPAIRED-based molecular probes follow the same steps).
